# Photochemical and thermochemical pathways to S_2_ and polysulfur formation in the atmosphere of Venus

**DOI:** 10.1038/s41467-022-32170-x

**Published:** 2022-07-30

**Authors:** Antonio Francés-Monerris, Javier Carmona-García, Tarek Trabelsi, Alfonso Saiz-Lopez, James R. Lyons, Joseph S. Francisco, Daniel Roca-Sanjuán

**Affiliations:** 1 Departament de Química Física, Universitat de València, 46100 Burjassot, Spain; 2 Institut de Ciència Molecular, Universitat de València, 46071 València, Spain; 3 Department of Atmospheric Chemistry and Climate, Institute of Physical Chemistry Rocasolano, CSIC, 28006 Madrid, Spain; 4 Department of Earth and Environmental Sciences and Department of Chemistry, University of Pennsylvania, Philadelphia, PA 19104 USA; 5 Planetary Science Institute, Tucson, AZ USA

**Keywords:** Atmospheric chemistry, Photochemistry, Computational chemistry

## Abstract

Polysulfur species have been proposed to be the unknown near-UV absorber in the atmosphere of Venus. Recent work argues that photolysis of one of the (SO)_2_ isomers, *cis*-OSSO, directly yields S_2_ with a branching ratio of about 10%. If correct, this pathway dominates polysulfur formation by several orders of magnitude, and by addition reactions yields significant quantities of S_3_, S_4_, and S_8_. We report here the results of high-level ab-initio quantum-chemistry computations that demonstrate that S_2_ is not a product in *cis*-OSSO photolysis. Instead, we establish a novel mechanism in which S_2_ is formed in a two-step process. Firstly, the intermediate S_2_O is produced by the coupling between the S and Cl atmospheric chemistries (in particular, SO reaction with ClS) and in a lesser extension by O-abstraction reactions from *cis*-OSSO. Secondly, S_2_O reacts with SO. This modified chemistry yields S_2_ and subsequent polysulfur abundances comparable to the photolytic *cis*-OSSO mechanism through a more plausible pathway. Ab initio quantification of the photodissociations at play fills a critical data void in current atmospheric models of Venus.

## Abstract (web summary)

Polysulfur compounds have been ascribed as the unknown near-UV absorbers in Venusian atmosphere and play a key role in the sulfur chemical cycle of this planet. Here, authors establish their production from (SO)_2_ on the grounds of quantifications of photochemical and thermal pathways involved in the sulfur chemical cycle of the planet.

## Introduction

Spacecraft missions and Earth-based observations have reported images of the Venusian atmosphere at different wavelengths with the aim of characterizing the cloud morphology and the exotic chemical processes that take place in this planet. At visible wavelengths, the planet is bland and smooth, whereas recordings at near-ultraviolet light (around 365 nm) reveal intriguing “dark” and “light” areas indicating highly active UV absorption in the top clouds^1–8^.

The identity of the “unknown UV absorber(s)” in Venus represents an important enigma in the current field of research on planetary atmospheres. Even though several particulate and gaseous candidates have been proposed, the nature of the unidentified absorber remains an unsolved problem as no satisfactory match has been found yet in terms of atmospheric abundance and spectral properties. Since sulfur is abundant in the Venusian atmosphere, it is not surprising that many candidates are sulfur-based compounds^1,3–5,9–15^. It has been proposed that ^1^(SO)_2_ isomers (mostly *cis*- and *trans*-OSSO) generated from the association reaction of ^3^SO (Eq. 1),

1 $${}^{3}{{{{{\rm{SO}}}}}}+{}^{3}{{{{{\rm{SO}}}}}}\to {}^{1}{({{{{{\rm{SO}}}}}})}_{2}$$

could be the UV absorber since the calculated absorption spectra match previous estimates of the spectral properties of the unknown UV absorber^1,9,16^. The short photochemical lifetimes of ^1^(SO)_2_ (between 2 and 5 s) derived from photolysis rates^16^ imply a short existence of these species during daytime, nevertheless, the chemical equilibrium with ^3^SO will be continuously replenishing ^1^(SO)_2_. Since both ^3^SO association (Eq. 1) and photochemical dissociation to ^3^SO + ^3^SO are swift processes, the global ^1^(SO)_2_ lifetime is controlled by ^3^SO concentrations^1,16–19^. These can vary greatly, in agreement with observations^4,20^. Since the lifetimes of the UV dark features range from minutes to hours, to an excess of 12 h^20^, a long-lived absorber is unlikely since short lived features would then not be possible. Therefore, the UV absorber concentration is likely tied to something other than direct sunlight, which matches the ^1^(SO)_2_ behavior dependent on the ^3^SO levels. Recent literature has brought into question the level of contribution of the two ^1^OSSO isomers to the UV absorption^17–19,21^, pointing to a too low ^3^SO concentration at lower altitudes of the Venus middle atmosphere (ca. 60 km). However, there is still significant uncertainty in these ^3^SO data, and ^1^OSSO role as the UV absorber has not been fully refuted yet.

Using data from the *Venus Express* mission and a photochemical model, Pinto et al.^19^ have recently assessed in detail the role of ^3^SO dimers as key intermediates in the production of polysulfur (S_*n*_) and polysulfur oxides (S_*n*_O). Polysulfur species are presented by the authors as solid candidates accounting for the enigmatic UV absorption on Venus, apart from being of relevance in other processes such as the production of S_*n*_O or key to explain the ^1^SO_2_ inversion layer observed in the Venusian atmosphere at altitudes above 90 km^12,13,21^. The model considers the photodissociation of the most predominant isomer of ^1^(SO)_2_, *cis*-OSSO, into ^3^S_2_ and ^3^O_2_ as a source of diatomic sulfur (Eq. 2):

2 $${}^{1}cis-{{{{{\rm{OSSO}}}}}}+{{{{{\rm{h{\nu}}}}}}}\to {}^{3}{{{{{{\rm{S}}}}}}}_{2}+{}^{3}{{{{{{\rm{O}}}}}}}_{2}$$

while subsequent self-propagation polymerizations of ^3^S_2_ give access to S_*n*_.

The photoreaction shown in Eq. 2 is proposed based on recent results reported by Wu et al.^11^ who studied the photoconversions of several ^1^(SO)_2_ isomers condensed on a cryogenic N_2_-matrix from gaseous ^3^SO. ^3^S_2_ in Wu’s experiment initially came from dissociation of the ethylene episulfoxide used as source of ^3^SO. Yellow light irradiation (579 nm) slightly decreased the peak at 287 nm attributed to ^3^S_2_. Further irradiation by 365 nm light depleted the 375 nm band with a simultaneous increase of the 287 nm band. The 375 nm band was assigned to *cis-*OSSO; however, recently Frandsen et al.^9^ have suggested that the *cis* isomer only accounts for the part of the band at wavelengths shorter than 360 nm, while the other part at lower energies is likely to be originated by *trans*-OSSO based on their simulated spectra for the *trans* isomer^9^. Regarding the increase of the 287 nm band, it was ascribed to the production of ^3^S_2_ due to the characteristic vibrational structure shown by the absorption band, motivating in this way the proposal of photoreaction (Eq. 2) by Pinto et al.^19^. Nevertheless, Wu et al. also pointed to other decomposition products (*cyclic*-OS(=O)S, SO_2_, C_2_H_4_, SO) as contributors to the 287 nm strong absorption and irradiation times lasted up to several tens of minutes^11^. Furthermore, extrapolation of the observations of photochemical experiments in matrix environments with long irradiation times to atmospheric conditions is not straightforward. Comparable experiments (low-temperature and solid matrixes) focused on HOSO radical photochemistry have given rise to different photodissociation channels after laser irradiation (H + OSO^22^ or HO + SO^23,24^). Hence, in spite of the recent advances, a convincing mechanism for the generation of S_*n*_ and S_*n*_O from ^1^(SO)_2_ isomers is still missing.

In this work, we identify the main photochemical routes of ^1^(SO)_2_ isomers by accurately simulating the gas-phase conditions of the Venus atmosphere through non-adiabatic molecular dynamics (NAMD)^25–31^, a well-established state-of-the-art methodology^32–42^, especially in combination with multiconfigurational quantum chemistry^43–47^. For HOSO^48,49^ and HOSO_2_^50,51^ radical photochemistry, NAMD has recently demonstrated that the ejection of OH is the main photodissociation channel for both systems in Earth’s atmosphere^24,52^. Guided by the photochemical outcomes, in the present work we also use multiconfigurational quantum chemistry to improve the conventional kinetics description of other reactions that give access to ^3^S_2_ through deoxygenation reactions of ^1^(SO)_2_ mediated by ^3^SO (Eqs. 3 and 4):

3 $${}^{1}cis-{{{{{\rm{OSSO}}}}}}+{}^{3}{{{{{\rm{SO}}}}}}\to {}^{3}{{{{{{\rm{S}}}}}}}_{2}{{{{{\rm{O}}}}}}+{}^{1}{{{{{{\rm{SO}}}}}}}_{2}$$

4 $${}^{1}{{{{{{\rm{S}}}}}}}_{2}{{{{{\rm{O}}}}}}+{}^{3}{{{{{\rm{SO}}}}}}\to {}^{3}{{{{{{\rm{S}}}}}}}_{2}+{}^{1}{{{{{{\rm{SO}}}}}}}_{2}$$

and other species X=NO, O, S, H present in Venus’ atmosphere^12,17,21,53^. We also add high-level quantum chemistry information for the reaction involving Cl (Eq. 5), which is an important source of S_2_O (see Pinto et al.^19^):

5 $${}^{2}{{{{{\rm{ClS}}}}}}+{}^{3}{{{{{\rm{SO}}}}}}\to {}^{1}{{{{{{\rm{S}}}}}}}_{2}{{{{{\rm{O}}}}}}+{}^{2}{{{{{\rm{Cl}}}}}}$$

## Results

### (SO)_2_ excited-state dynamics

**Fig. 1:**
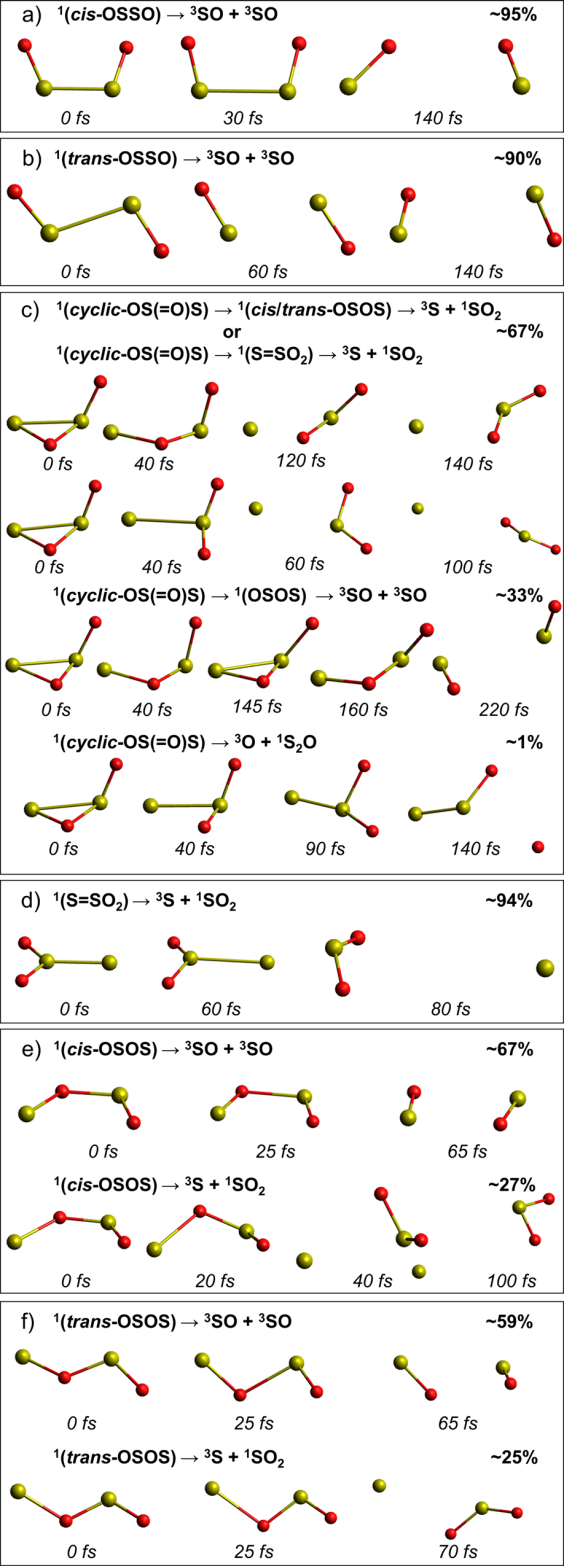
Time evolution and photolysis yields of (SO)_2_ isomers. **a** *cis*-OSSO and **b** *trans*-OSSO non-adiabatic molecular dynamics (NAMD) were computed with the multi-state complete-active-space second-order perturbation theory (MS-CASPT2) method (SHARC2.1/OpenMolcas)^25,46^, whereas the excited-state dynamics of the other systems (**c**–**f**) were run using the time-dependent density functional theory (TD-DFT) method (SHARC2.1/Gaussian 16)^25,71^. Photodynamics of *cis*-OSSO and *trans*-OSSO are clearly dominated by photocleavage to ^3^SO + ^3^SO, while ^3^S production arises also as another relevant light-induced decomposition product in *cyclic*-OS(=O)S, S=SO_2_, *cis*-OSOS, and *trans*-OSOS. Note that the spin multiplicity of the photoproducts corresponds to their respective ground-state, assumed to be reached at the end of the photodissociation process. The sensitivity of the yields to the number of trajectories is analyzed in Supplementary Table 4. See time evolution of relevant bond distances along the simulations in Supplementary Figs. 6, 7, 9, and 18–23. Red and yellow balls represent oxygen and sulfur atoms, respectively.

The distribution of the *cis*- and *trans*-OSSO photoproducts simulated for the atmosphere of Venus, and that of other relevant isomers, is shown in Fig. 1a, b, including quantum yields and illustrative snapshots of the geometries along the dynamics (see also Supplementary Tables 1 and 4). Note that the yields can be directly implemented as quantum yields for the photoinduced dissociations in the present and future Venus atmospheric modelings. Excitation of *cis*-OSSO and *trans*-OSSO in the atmospherically relevant window (310–496 nm), which mainly populates their second excited electronic state (*S*_*2*_), triggers exclusively photodissociations into ^3^SO + ^3^SO in less than 140 fs, with quantum yields of ca. 95% and 90%, respectively. These results clearly establish previous proposals by Frandsen et al.^9^. The decay of the *S*_*2*_ state into the energetically lower *S*_*1*_ state is faster in the *cis* isomer as compared to the *trans* species (Supplementary Figs. 8 and 10, respectively), whereas the triplet population is negligible for both isomers. Within our simulation time, 5% of *cis*-OSSO and 10% of *trans*-OSSO did not show any photolysis. Instead, the systems showed motions that could lead either to photoisomerizations (e.g., *cis* ↔ *trans* or *cyclic*-OS(=O)S) or to non-reactive decays. S–O bond breakings in the populated low-lying states are hindered by high-energy requirements (Supplementary Fig. 12).

Excitation at 225–496 nm of the *cyclic*-OS(=O)S species (which has been recently detected^11^ in the laboratory using matrix isolation spectroscopy) populates mostly the *S*_*4*_ and *S*_*5*_ states (Supplementary Fig. 24), producing a mixture of photoproducts (Fig. 1c). The dominant (67%) photodissociation gives rise to ^3^S + ^1^SO_2_, whereas 33% leads to ^3^SO + ^3^SO via either *cis*-/*trans*-OSOS or the trigonal isomer S=SO_2_. Surprisingly, 1% of molecules produced ^1^S_2_O + ^3^O, a reaction that has not been documented until now.

As expected, the trigonal S=SO_2_, the most stable ^1^(SO)_2_ isomer in the ground-state^9,54,55^, photodissociates exclusively into ^3^S + ^1^SO_2_ when irradiated at 225–496 nm (Fig. 1d), confirming the proposal of ^1^(SO)_2_ photolysis from Krasnopolsky et al. based on theoretical estimations of energy profiles^53,54^. On the other hand, excitation (310–496 nm) of *cis*-OSOS (Fig. 1e) activates the dominant fragmentation into ^3^SO + ^3^SO (67%), whereas the photolysis into ^3^S + ^1^SO_2_ is less competitive (27%) although significant. A similar scenario is found for *trans*-OSOS (Fig. 1f).

Overall, the photodynamics of ^1^(SO)_2_ dimers give rise mainly to ^3^SO, while ^3^S atoms are produced from ^3^SO photodissociation (Supplementary Table 13) and are also generated indirectly from the minor isomeric photoproducts of *cis-/trans*-OSSO (*cyclic*-OS(=O)S, S=SO_2_, *cis*-OSOS, and *trans-*OSOS) as found herein from the simulations. Considering the photolysis rate of *cis*-OSSO and *trans*-OSSO of 0.20 and 0.62 s^−1^ (ref. 16), respectively, and the yields computed in this work for the non-photoreactive species (Fig. 1), an upper limit of 0.01–0.03 s^−1^ can be established for the rate of photoproduction of ^3^S from ^1^(SO)_2_. Such ^3^S atoms could recombine giving rise to ^3^S_2_ via reaction (Eq. 6) or oxidized by ^3^O_2_ (Eq. 7). Measured 3-body rate constants for reaction (Eq. 6) differ by nearly a factor of 10^4^ (see Supplementary Table 13). Nevertheless, as shown in Supplementary Table 6, when considering high-pressure conditions for (Eq. 6) and the concentrations of ^3^S and ^3^O_2_ at the relevant altitude of ~64 km from Zhang et al.^12^, the pseudo-first order rate (*k*) appears lower for ^3^S recombination (Eq. 6) than that for oxidation (Eq. 7).

6 $${}^{3}{{{{{\rm{S}}}}}}+{}^{3}{{{{{\rm{S}}}}}}+{{{{{\rm{M}}}}}}\to {}^{3}{{{{{{\rm{S}}}}}}}_{2}+{{{{{\rm{M}}}}}}$$

7 $${}^{3}{{{{{\rm{S}}}}}}+{}^{3}{{{{{{\rm{O}}}}}}}_{2}\to {}^{3}{{{{{\rm{SO}}}}}}+{}^{3}{{{{{\rm{O}}}}}}$$

### ^3^SO deoxygenation reactions

**Fig. 2:**
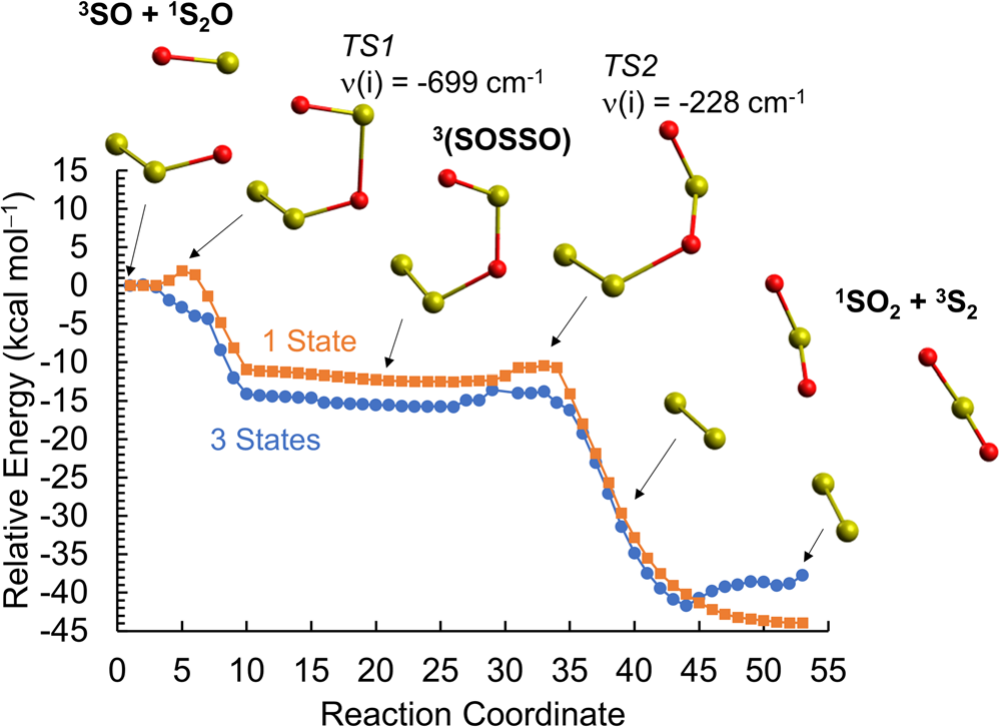
^3^SO + ^1^S_2_O → ^1^SO_2_ + ^3^S_2_ reaction energy profile. Two approaches of multiconfigurational quantum chemistry (CASPT2) are shown, with a wavefunction based only on the lowest-lying electronic state (1 State) or a wavefunction allowing the interaction between the 3 lowest-lying nearby states (3 States). Benchmark analyses demonstrate the latter to be of higher accuracy, while the former is used as sensitivity test (see section 4.1 in the SI). A two-step process is found via an intermediate adduct ^3^(SOSSO) involving energy barriers heights of at most ~2 kcal mol^−1^ for both approaches to access the transition states for the adduct formation (*TS1*) and S–O bond cleavage from the adduct (*TS2*). The values of the imaginary frequency that characterize the TSs are also shown. Red and yellow balls represent oxygen and sulfur atoms, respectively.

Taking into account the significant photoproduction of ^3^SO from *cis*-/*trans*-OSSO, *cyclic*-OS(=O)S, and *cis*-/*trans*-OSOS (Fig. 1), formed in situ, we characterized the thermal (i.e., ground-state) deoxygenation reactions (Eqs. 3 and 4) that yield ^1^S_2_O, ^3^S_2_, and ^1^SO_2_ (Fig. 2, Supplementary Figs. 25 and 27, and Supplementary Table 6), which open the path toward aerosols (S_*n*_O and S_*n*_). Since other species (^2^NO, ^3^O, ^3^S, and ^2^H) could also produce analogous reactions^12,19,53^, we accurately determined their reactivity with *cis*-OSSO (Supplementary Table 6 and Supplementary Figs. 28–35) to update previous estimations for the rate of these reactions. Furthermore, reaction between the abundant ^3^SO species and ^2^ClS (Eq. 5), which gives ^1^S_2_O, and subsequently ^3^S_2_ via (Eq. 4), was also characterized (Supplementary Figs. 39–41). The strong electron correlation character of the involved species forbids the general use of the single-reference DFT- or CC-based methodologies, which are popular in this field, requiring high-level multiconfigurational quantum chemistry (see analyses in the Supplementary Note 7). The obtained results indicate that reaction (Eq. 3) generates ^1^S_2_O with an energy barrier of ~9 kcal mol^−1^, in reasonable agreement with the estimates from reported rate constants (~7.5 kcal mol^−1^)^56^. Production of ^1^S_2_O by the reaction between *cis-*OSSO and ^2^NO, ^3^O, and ^3^S must overcome higher energy barrier heights (Supplementary Table 6), while a lower value is found for the reaction with ^2^H. On the contrary, reaction in Eq. 5 shows no activation energy. Once ^1^S_2_O is produced, the subsequent reaction to produce ^3^S_2_ shows a low-energy barrier especially for the reaction with ^3^SO (Eq. 4), as shown by the energy profile in Fig. 2. Analogous reaction energetics should apply for *trans*-OSSO.

The complex thermal reactivity between two *cis*-OSSO molecules was also explored (Supplementary Figs. 45–47), giving rise first to a cyclic species (*cyclic*-S_4_O_4_) by an energy barrier of ~9 kcal mol^−1^ and next to *cis*-S_3_O_2_ and ^1^SO_2_ involving a barrier of ~12 kcal mol^−1^ (Supplementary Table 6). Further reaction of ^1^*cis*-S_3_O_2_ with ^3^SO becomes faster (energy penalty of ~5 kcal mol^−1^) and ultimately results in ^1^S_*n*_O molecular systems and ^1^SO_2_ (Supplementary Fig. 48). Note also that S_*n*_O can photolyze to S_*n*_ (Supplementary Fig. 42).

### Photochemical steady-state model for Venus middle atmosphere

With our ^1^(SO)_2_ photoreaction rates and branching ratios and the thermal reactivity properties, an approximate photochemical steady-state model was built to determine the reaction rate profiles and number densities for the key sulfur species for Venus middle atmosphere. Apart from ^1^SO_2_, most middle and upper atmosphere sulfur species have photochemical lifetimes shorter than their vertical transport timescales, making a steady-state approximation viable. Using ^1^SO_2_ number density and photodissociation rate profiles from Zhang et al.^12^ we computed steady abundances of ^3^SO, *cis-*OSSO, ^1^S_2_O, and ^3^S_2_ (see Supplementary Note 8). Number densities of ^2^NO, ^3^O, ^3^S, ^2^H, and ^2^ClS reported by Zhang et al.^12^ were also used. We treated ^3^S_2_ as a proxy for sulfur aerosols, but we did not include sulfur allotrope condensation reactions.

According to the calculated pseudo-first order rates (*k*) (Supplementary Table 6), thermal reactivity of *cis*-OSSO (*k* < 10^−4 ^s^−1^) is clearly much slower than its photochemistry (0.19 s^−1^). This confirms previous assessments made by Frandsen et al.^4^ based on their calculated photochemical lifetime (0.20 s^−1^)^4,16^ and the thermal reactivity of OSSO reported by Yung and DeMore^57^. In the present work, we expand the amount of thermal reactions and the level of accuracy, which clearly strengthen the argument that OSSO photochemistry dominates over thermochemistry. Regarding the comparison for the thermal reactivity between *cis-*OSSO and ^3^SO/^2^NO/^3^O/^3^S/^2^H (see reaction rates in Supplementary Figs. 54 and 55), ^3^SO has a rate clearly higher than the others. As can be seen in Supplementary Table 6, for ^2^NO, ^3^O, and ^3^S, the reason is their larger energy barrier heights, while for ^2^H, it can be associated with the much lower abundance of this species (Supplementary Table 8). For reaction between ^2^ClS and ^3^SO (Eq. 5), the reaction rate (Supplementary Figs. 54 and 55) exceeds the rates of the other processes. Meanwhile, the lower concentration obtained for *cis*-OSSO in comparison to ^3^SO gives rise also to a lower *k* (Supplementary Table 6), and associated reaction rate, for the reactivity initiated by two *cis*-OSSO molecules as compared to that by ^3^SO or as compared to (Eq. 5).

**Fig. 3:**
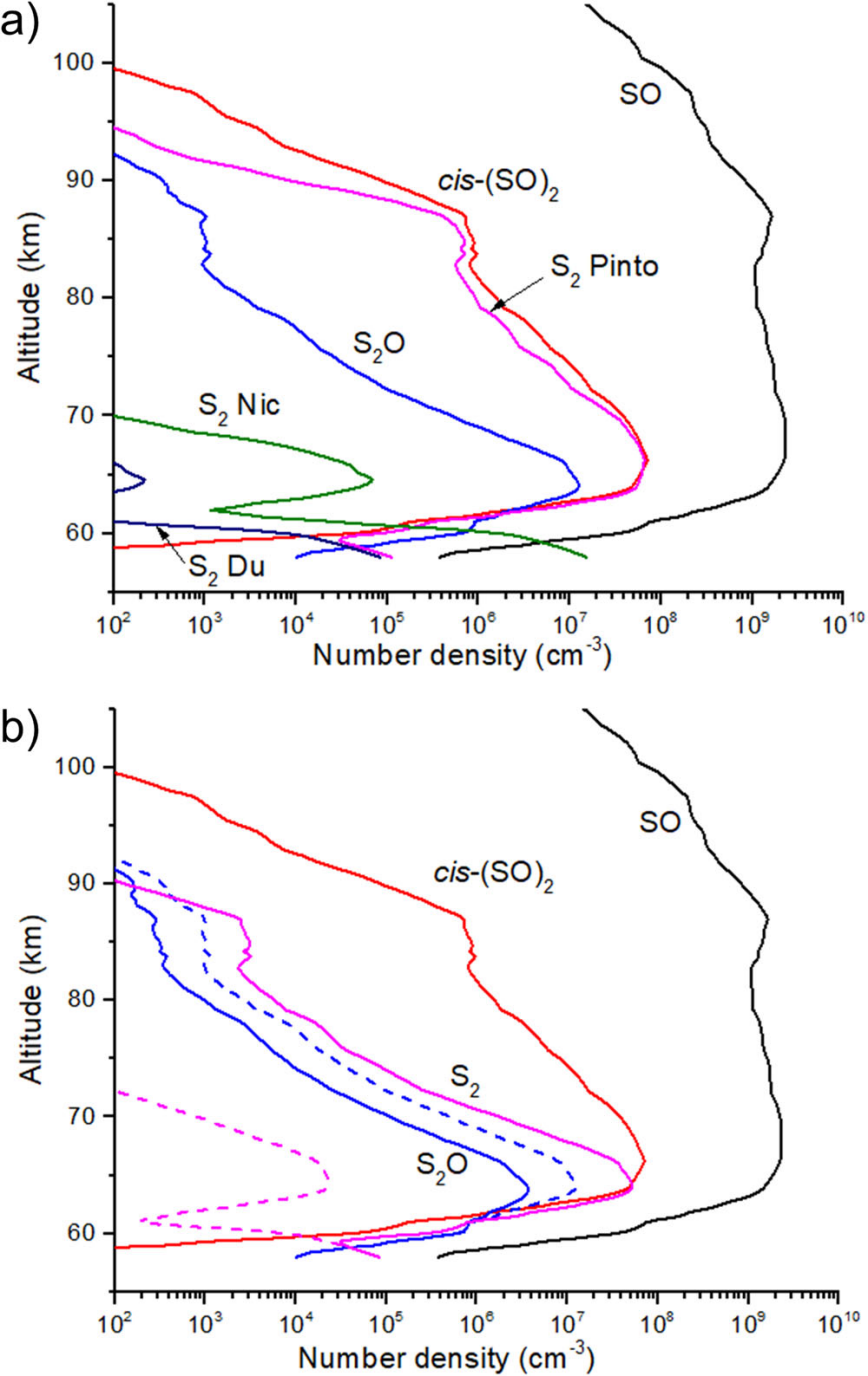
Estimated steady-state profiles for several key sulfur species. **a** S_2_ profiles for several possible model conditions. “S_2_ Pinto” is derived from photolysis of *cis*-OSSO as in Pinto et al.^19^. Turning off this reaction and using a fast ^3^S + ^3^S rate coefficient^79^ yields “S_2_ Nic”, and using a slower ^3^S + ^3^S rate coefficient^80^ yields “S_2_ Du”. In both cases ^3^S_2_ is dramatically reduced. **b** With the Pinto et al. S_2_ formation mechanism turned off, reaction of ^3^SO with ^1^S_2_O becomes the dominant pathway for ^3^S_2_. ^3^S_2_ and ^1^S_2_O profiles are shown for a rate constant of 1 × 10^−10^ cm^3^ s^−1^ (solid lines) and for a rate constant of 1 × 10^−14^ cm^3^ s^−1^ (dashed lines). The higher rate constant is consistent with ab initio calculations presented here and yields substantial ^3^S_2_.

Figure 3a displays the number density profiles obtained for the key sulfur species and comparative analysis with those previously reported. For the assumption of 10% ^3^S_2_ produced during *cis-*OSSO as in Pinto et al.^19^, the ^3^S_2_ profile closely tracks the *cis*-OSSO profile (Fig. 3a). If we instead assume zero ^3^S_2_ photoproduction and allow for ^3^S_2_ production primarily from ^3^S atom recombination, we obtain an ^3^S_2_ number density a factor of 10^4^ lower (or lower) at 65 km, depending on the 3-body rate coefficient used for ^3^S atom recombination. At greater depth ^3^S_2_ abundance rises but to a value dependent on the widely varying published 3-body rate constants (Fig. 3a).

With zero ^3^S_2_ production from *cis-*OSSO photolysis, we included reaction (Eq. 4) for several possible values of the rate constant. Ab initio reactivity calculations presented here (Supplementary Table 6) indicate that reaction (Eq. 4) is fast. Evaluating (Eq. 4) over a span of 4 orders of magnitude to consider estimations from similar reactions (Supplementary Table 13) and the updated value in the present work yields a peak ^3^S_2_ abundance comparable to that reported by Pinto et al.^19^ for the highest rate constant, but with a more narrow vertical distribution (Fig. 3b).

## Discussion

The photochemistry study of ^1^(SO)_2_ isomers, herein performed with state-of-the-art photodynamics simulations reproducing Venus’ atmosphere conditions, points to an overwhelming prevalence of the S–S photodissociations over the S–O ruptures, which must be attributed to the larger S–O bond strength in comparison to that of S–S. Clearly, the S–S cleavage drives the photochemistry of ^1^(SO)_2_. The findings (displayed in Fig. 1) show that the major photoproducts are ^3^SO, ^3^S, and ^1^SO_2_. This seriously questions a significant occurrence of (Eq. 2), as assumed by Pinto et al.^19^ on the basis of experiments by Wu et al.^11^ in cold conditions and solid matrixes, since no ^3^S_2_ + ^3^O_2_ production has been observed in our thorough simulations. As a matter of fact, the release of a single oxygen atom rarely takes place. Therefore, it is reasonable to conclude that the reaction shown in Eq. 2 is not able to explain a significant generation of polysulfur reactions in Venus through irradiation of ^1^(SO)_2_ species. The more usual mechanism for ^3^S_2_ formation is ^3^S atom recombination (Eq. 6). ^3^S atoms are produced from ^3^SO photodissociation and are also generated indirectly from the minor isomeric photoproducts of *cis-/trans*-OSSO (*cyclic*-OS(=O)S), S=SO_2_, *cis*-OSOS, and *trans-*OSOS) as obtained in the present study (Fig. 1). However, polysulfur production by Eq. 6 is limited when formation of allotropes of size ^3^S_2_ and larger must proceed through this pathway as ^3^S atoms are lost rapidly through oxidation by ^3^O_2_ (Eq. 7). This ^3^S atom bottleneck greatly prevents significant production of polysulfur species in the Venusian atmosphere from this source.

Contrary to the situation in Venus’ atmosphere, the production of ^3^S_2_ in matrix monitored by Wu et al.^11^ through the absorption increase at 287 nm seems to be the result of the recombination (Eq. 6), fed by the sulfur atoms largely released by four out of six ^1^(SO)_2_ isomers (Fig. 1c–f), whereas the depletion of the 375 nm absorption band is likely the result of the fragmentations exposed in Fig. 1. It must be emphasized that the experimental conditions (cold molecules adsorbed on a solid matrix) are not strictly comparable to our simulations in the gas-phase at standard temperature neither to those of the Venus atmosphere, even more considering the extremely long irradiation times (tens of minutes) used by Wu et al.^11^ as compared to the ultrafast photodissociations studied in our work.

Instead of an ^3^S atom pathway, we propose that ^3^S_2_ formation in the atmosphere occurs primarily from the precursor ^1^S_2_O by Eq. 4, which is generated by Eqs. 3 and 5, and other sources reported in the literature (see Supplementary Figs. 54 and 55). This is motivated by the fact that when switching on our highly accurate energetics and rate for Eq. 4 computed herein and ^1^S_2_O generation rates available in the literature (supported in this work for the relevant Eqs. 3 and 5 by quantum chemistry profiles) in our photochemical steady-state model of the Venus middle atmosphere, ^3^S_2_ abundance increases 4 orders of magnitude approaching the number density reported by Pinto et al.^19^ but without the photochemical pathway shown in Eq. 2. A similar peak of ^3^S_2_ abundance is found although the vertical distribution is narrower in our case. We can clearly state that the sulfur cycle in the Venus middle atmosphere can maintain a high fraction of elemental sulfur aerosols by converting ^3^SO or ^1^(SO)_2_ to ^3^S_2_ via the ^1^S_2_O intermediate and the coupling between S and Cl chemistry in Venus. Assessment of deoxygenation pathways analogous to Eqs. 3 and 4 but mediated by ^2^NO, ^3^O, ^3^S, and ^2^H shows that they are not competitive with ^3^SO. Therefore, ^3^SO is a more efficient agent in comparison to ^2^NO, ^3^O, ^3^S, and ^2^H to convert thermally *cis-*OSSO (predominant ^1^(SO)_2_ isomer in Venus’ atmosphere) first into ^1^S_2_O by Eq. 3 and subsequently to ^3^S_2_ by Eq. 4. Nevertheless, the most efficient process leading to the intermediate ^1^S_2_O involves the reaction between ^3^SO and ^2^ClS (Eq. 5). Reactivity of two *cis*-OSSO molecules can produce S_*n*_O, S_*n*_, and ^1^SO_2_ via a complex mechanism as shown herein. However, it does not compete with reactions shown in Eqs. 3–5, also since this reaction is limited by the lower concentration of *cis*-OSSO in the Venusian atmosphere predicted by the present modeling. This reactivity can be expected during the night or in conditions of weak radiation, and it should contribute to the reported day/night variation of the ^1^SO_2_/^3^SO concentration ratio^58^.

The findings obtained in this study allow for a more complete understanding of the current Venusian atmosphere. Because of the complexity of the sulfur chemistry in the middle atmosphere, which includes many species of the form S_*x*_O_*y*_Cl_*z*_, and because of the paucity of laboratory rate coefficient data for most of the reactions among these species, high-level ab initio calculations are essential for understanding the chemistry of the Venus atmosphere. Those improvements are needed also to guide the next terrestrial measurements, spacecraft missions (recently announced by the National Aeronautics and Space Administration motivated in part by the studies on the phosphine detection in Venus atmosphere)^59–63^, and to assist with geoengineering of Earth’s climate and monitor stratospheric volcanic eruptions’ clouds. Furthermore, they are beneficial to better understand the atmosphere of early Earth (pre-oxygenation of Earth’s atmosphere) and shall be used in describing Earth-like exoplanet atmospheres in conjunction with future high-resolution spectroscopy measurements of said exoplanets.

## Methods

### Thermal and photochemical reaction channels and associated rates

Herein, photolysis of ^1^(SO)_2_ has been studied in detail by means of NAMD propagated with the multi-state complete-active-space second-order perturbation theory (MS-CASPT2) method^43,44,64^ with the OpenMolcas and SHARC programs^25,46^, including the mixing of singlet and triplet states (see active space orbitals in Supplementary Fig. 1). Multireference configuration interaction (MRCI)^47,65,66^ profiles validate this level of theory (Supplementary Figs. 2–5). These high-level results have been used to validate NAMD simulations propagated at the faster time-dependent density functional theory (TD-DFT) method^67–70^ with the Gaussian and SHARC programs^25,71^ (Supplementary Figs. 11–17 and Supplementary Table 3). Simulation settings and method validations are fully detailed in the Supplementary Information (SI), Supplementary Notes 1–3 and Supplementary Tables 2 and 3^72–77^.

Minima and transition states that describe the thermal reaction profiles have been optimized for the lowest-lying electronic state, with the single-state (SS)-CASPT2 method using state-specific complete-active-space self-consistent field (SS)-CASSCF wave functions as reference. The nature of the stationary points has been verified through the corresponding frequency analyses whereas the transition states have been unambiguously connected with their corresponding reactants and products through intrinsic reaction coordinates determinations. To allow the coupling between nearby electronic states, state-average (SA)-CASSCF wave functions have been computed on top of the mentioned optimized structures and the final energy profiles were corrected with the MS-CASPT2 method using the same protocol as for the SS-CASSCF determinations. The strong electron character and the multiconfigurational nature of some of these ground-state reactions have been evidenced by comparisons with the DFT and coupled-cluster methods used in Gaussian^71^ and ORCA^78^. Static excited-state pathways and ground-state reaction channels with problematic transition-state optimizations have been explored through relaxed scans of the reaction coordinate or by means of linear interpolations of internal coordinates between relevant structures. Further information on the specific computational parameters (active spaces, basis sets, number of computed states), the employed optimization algorithms, and data used for computing rates can be found in the SI, Supplementary Notes 4–7, Supplementary Figs. 25–50 and Supplementary Tables 5–12.

### Photochemical steady-state calculations: abundances of sulfur species

We use photochemical steady-state calculations to estimate the abundance profiles for several sulfur species of particular importance in the Venus atmosphere. As the photochemical lifetimes of many of the trace sulfur species are shorter than the eddy transport timescale, this approximation is valid to first order, and allows us a rapid assessment of the implications of the new chemical schemes proposed here based on our ab initio results. For non-sulfur species, long-lived sulfur species, and photodissociation rate constants, we use profiles from Pinto et al.^19^ and Zhang et al.^12^. Mixing ratio profiles for [^3^O], [^3^O_2_], [^2^NO], [^2^H], [^1^SO_2_], [^3^S], and [^2^ClS] are digitally read-in from Zhang et al.^12^ or Pinto et al.^19^. The vertical profiles for the photodissociation rate coefficients for ^3^SO and ^1^SO_2_ were taken from the bibliography as described in Supplementary Note 8. Condensation reactions for sulfur allotropes have not been included here, as appears to be the case for Pinto et al.^19^. Photochemical steady-state calculations are carried out from 58 to 112 km, following the temperature and total number density profiles given by Zhang et al.^12^. Steady-state number densities are computed for ^3^SO, ^1^(SO)_2_, which we assume in these calculations to be primarily *cis*-(SO)_2_, ^1^S_2_O, and ^3^S_2_. A reduced set of 19 reactions involving these species is given in Supplementary Table 13. Setting production rate equal to loss rate for each of the 4 species of interest, and using the reactions in Supplementary Table 13, we arrive at the Equations for steady-state number density S1–S4. These Equations are solved in the order presented in the SI, Supplememntary Note 8, and the steady-state values are used as applicable. Loss of ^3^S_2_ to S_4_ formation is accounted for, but we are not attempting to accurately account for sulfur allotrope abundances. For this reason, ^3^S_2_ may be taken as a proxy for total sulfur aerosol production. Further details on the photochemical steady-state model and benchmarking analyses can be found in the SI, Supplementary Note 8 and Supplementary Figs. 51–53.

## Supplementary information

Supplementary Information

## Data Availability

All data to evaluate the conclusions in the paper are available in the main text and/or the Supplementary Materials.
