# Supplementary material for: Photochemical and thermochemical pathways to S2 and polysulfur formation in the atmosphere of Venus
Source: Nat Commun. 2022 Jul 30;13:4425. doi: 10.1038/s41467-022-32170-x (PMC9338966; doi:10.1038/s41467-022-32170-x)
Supplement: Supplementary file 1 — Supplementary Information [file 41467_2022_32170_MOESM1_ESM.pdf]

Supplementary Information for:

Photochemical and thermochemical pathways to S_2 and polysulfur formation in the atmosphere of Venus

Antonio Francés-Monerris,[1]* Javier Carmona-García,[2,3] Tarek Trabelsi,[4] Alfonso Saiz-Lopez,[3] James R. Lyons,[5]* Joseph S. Francisco,[4]* Daniel Roca-Sanjuán[2]*

[1] Departament de Química Física, Universitat de València, 46100 Burjassot, Spain

[2] Institut de Ciència Molecular, Universitat de València, València 46071, Spain

[3] Department of Atmospheric Chemistry and Climate, Institute of Physical Chemistry Rocasolano, CSIC, Madrid 28006, Spain

[4] Department of Earth and Environmental Sciences and Department of Chemistry, University of Pennsylvania, Philadelphia, Pennsylvania 19104, United States

[5] Planetary Science Institute, Tucson, AZ, United States

*Corresponding Authors: A.F.-M. Antonio.Frances@uv.es, J.L. jlyons@psi.edu, J.S.F. frjoseph@sas.upenn.edu, D.R.-S. Daniel.Roca@uv.es

Table of Contents

1. Supplementary Note 1. Computational details of the MRCI profiles 6

2. Supplementary Note 2. MS-CASPT2 non-adiabatic molecular dynamics. *Cis* and *trans*-OSSO .. 6

2.1 Simulation details 6

2.2 Method validation 8

2.3 Results 10

3. Supplementary Note 3. TD-DFT non-adiabatic molecular dynamics 13

3.1 Simulation details 13

3.2 Method validation 14

3.3 Results 24

4. Supplementary Note 4. Static ground-state CASPT2/MS-CASPT2 reactivity 32

4.1 Computational details and benchmark analyses 32

4.2 Results 34

5. Supplementary Note 5. Excited-state MS-CASPT2 energy profiles.. 44

6. Supplementary Note 6. Ground-state DFT reactivity.. 46

6.1 Computational details 46

6.2 Results 46

7. Supplementary Note 7. Coupled-cluster results 50

7.1 Computational details 50

7.2 Results 51

7.3 Influence of the wave function initial guess and spin density analysis.. 53

8. Supplementary Note 8. Estimation of sulfur species profiles in the atmosphere of Venus 54

8.1 Methodological details and data analyses 54

8.2 Further comments on the sensitivity of the photochemical steady state model.. 58

8.3 Origin of the rates included in the photochemical steady state model 60

9. Supplementary References 64

List of Supplementary Figures

Supplementary Figure	Page
**Supplementary Figure 1.** SA(4)-CASSCF natural orbitals employed in the MS-CASPT2 NAMD simulations for *cis*-OSSO. An analogous active space has been used for the simulation on the *trans* isomer.	S7
**Supplementary Figure 2.** MRCI+Q/aug-cc-pV(T+d)Z (a) and MS-CASPT2/ANO-S-VDZP (b) potential energy curves for *cis*-OSSO along the rigid scan of the OS-SO bond distance.	S8
**Supplementary Figure 3.** MRCI+Q/aug-cc-pV(T+d)Z (a) and MS-CASPT2/ANO-S-VDZP (b) potential energy curves for *cis*-OSSO along the rigid scan of the O-SSO bond distance.	S9
**Supplementary Figure 4.** MRCI+Q/aug-cc-pV(T+d)Z (a) and MS-CASPT2/ANO-S-VDZP (b) potential energy curves for *trans*-OSSO along the rigid scan of the OS-SO bond distance.	S9
**Supplementary Figure 5.** MRCI+Q/aug-cc-pV(T+d)Z (a) and MS-CASPT2/ANO-S-VDZP (b) potential energy curves for *trans*-OSSO along the rigid scan of the OS-SO bond distance.	S10
**Supplementary Figure 6.** Time evolution of the most relevant bond distances of *cis*-OSSO upon excitation to the S_1 state.	S11
**Supplementary Figure 7.** Time evolution of the most relevant bond distances of *cis*-OSSO upon excitation to the S_2 state.	S11
**Supplementary Figure 8.** Normalized populations of the 86 trajectories ensemble starting from S_2 for *cis*-OSSO.	S12
**Supplementary Figure 9.** Time evolution of the most relevant bond distances of *trans*-OSSO upon excitation to the S_2 state.	S12
**Supplementary Figure 10.** Normalized populations of the 70 trajectories ensemble starting from S_2 for *trans*-OSSO.	S13
**Supplementary Figure 11.** TD-B3LYP/6-31G* (top panel) and MS-CASPT2/ANO-S-VTZP (bottom panel) potential energy landscapes for the S1-S3 bond breaking of *cis*-OSSO.	S17
**Supplementary Figure 12.** TD-B3LYP/6-31G* (top panel) and MS-CASPT2/ANO-S-VTZP (bottom panel) potential energy landscapes for the S1-O2 bond breaking of *cis*-OSSO.	S18
**Supplementary Figure 13.** TD-B3LYP/6-31G* (top panel) and MS-CASPT2/ANO-S-VTZP (bottom panel) potential energy landscapes for the S1-S3 bond breaking of the trigonal $S{=}SO_2$.	S19
**Supplementary Figure 14.** TD-B3LYP/6-31G* (top panel) and MS-CASPT2/ANO-S-VTZP (bottom panel) potential energy landscapes for the S1-S3 bond breaking of *cyclic* OS(=O)S.	S20
**Supplementary Figure 15.** TD-B3LYP/6-31G* (top panel) and MS-CASPT2/ANO-S-VTZP (bottom panel) potential energy landscapes for the S3-O2 bond breaking of *trans*-OSOS.	S21
**Supplementary Figure 16.** TD-B3LYP/6-31G* (top panel) and MS-CASPT2/ANO-S-VTZP (bottom panel) potential energy landscapes for the S1-O2 bond breaking of *trans*-OSOS.	S22
**Supplementary Figure 17.** TD-B3LYP/6-31G* (top panel), potential (in the diagonal representation) and kinetic energy (mid panel), and MS-CASPT2/ANO-S-VTZP (bottom panel) potential energy landscapes for a *cyclic* OS(=O)S → S + SO_2 trajectory run with the TD-B3LYP/6-31G* NAMD.	S23
**Supplementary Figure 18.** Time evolution of the different bond distances for the *cis*-OSSO system computed with the TD-B3LYP/6-31G* NAMD.	S24
**Supplementary Figure 19.** Time evolution of the different bond distances for the *trans*-OSSO system computed with the TD-B3LYP/6-31G* NAMD.	S25
**Supplementary Figure 20.** Time evolution of the different bond distances for the *cyclic*-OS(=O)S system computed with the TD-B3LYP/6-31G* NAMD.	S26
**Supplementary Figure 21.** Time evolution of the different bond distances for the $S{=}SO_2$ system computed with the TD-B3LYP/6-31G* NAMD.	S27

**Supplementary Figure 22.** Time evolution of the different bond distances for the *cis*-OSOS system computed with the TD-B3LYP/6-31G* NAMD.	S28
**Supplementary Figure 23.** Time evolution of the different bond distances for the *trans*-OSOS system computed with the TD-B3LYP/6-31G* NAMD.	S29
**Supplementary Figure 24.** Time evolution of the populations for *cis*-OSSO (a), *trans*-OSSO (b), *cyclic*-OS(=O)S (c), S=SO_2 (d), *cis*-OSOS (e), and *trans*-OSOS (f).	S31
**Supplementary Figure 25.** ^{3}SO + ^{1}OSSO → $^1SO_2$ + ^3S_2O reaction profile.	S36
**Supplementary Figure 26.** CASSCF (1R) and SA-CASSCF (3R) Mulliken spin densities computing 1 and 3 states, respectively, along the first step of the ^{3}SO + ^{1}OSSO → $^1SO_2$ + ^3S_2O reaction.	S36
**Supplementary Figure 27.** ^{3}SO + ^{1}SSO → $^1SO_2$ + $^3S_2$ reaction profile.	S37
**Supplementary Figure 28.** ^{2}NO + ^{1}OSSO → ^{2}ONOSSO reaction profile.	S37
**Supplementary Figure 29.** ^{2}ONOSSO → $^2NO_2$ + ^{1}SSO reaction profile.	S38
**Supplementary Figure 30.** ^{3}O + ^{1}OSSO → $^3O_2$ + ^1S_2O reaction profile.	S38
**Supplementary Figure 31.** ^{3}O + ^{1}SSO → $^3[O_2 + S_2]$ reaction profile.	S39
**Supplementary Figure 32.** ^{3}S + ^{1}OSSO → ^{3}SO + ^1S_2O reaction profile.	S39
**Supplementary Figure 33.** ^{3}S + ^{1}SSO → $^3[SO + S_2]$ reaction profile.	S40
**Supplementary Figure 34.** ^{2}H + ^{1}OSSO → ^{2}HOSSO reaction profile.	S40
**Supplementary Figure 35.** ^{2}H + ^{1}SSO → ^{2}HSSO reaction profile.	S41
**Supplementary Figure 36.** ^{3}S + ^{3}OO → ^{1}SOO reaction profile.	S41
**Supplementary Figure 37.** ^{1}SOO → ^{3}SO + ^{3}O reaction profile.	S42
**Supplementary Figure 38.** ^{3}[S + S] → $^3S_2$ reaction profile.	S42
**Supplementary Figure 39.** ^{2}ClS + ^{3}SO → ^{2}ClSSO reaction profile.	S43
**Supplementary Figure 40.** ^{2}ClSSO → ^{2}Cl + ^{1}SSO reaction profile.	S43
**Supplementary Figure 41.** ^{2}ClS + ^{3}OS → ^{2}ClSOS reaction profile.	S44
**Supplementary Figure 42.** MS-CASPT2 relaxed scan of ^{1}*cis*-SSSO, relaxing the **S_2** state.	S44
**Supplementary Figure 43.** ^{2}HOSS → $^2[HO + S_2]$ excited-state reaction along the S-O bond stretch relaxing the D_2 state at the MS-CASPT2 level of theory.	S45
**Supplementary Figure 44.** ^{2}HOSS → ^{2}HOS + ^{3}S excited-state reaction along the S-S bond stretch relaxing the D_2 state at the MS-CASPT2 level of theory.	S45
**Supplementary Figure 45.** Reaction of two ^{1}*cis*-OSSO molecules to yield ^{1}*cis*-S_3O_2 and $^1SO_2$ (right), followed by the ^{1}*cis*-S_3O_2 decomposition to yield $^1SO_2$ and $^1S_2$ (left).	S47
**Supplementary Figure 46.** Exploration of the ***TS3*** region.	S48
**Supplementary Figure 47.** Exploration of the ***TS4*** region.	S48
**Supplementary Figure 48.** ^{1}*cis*-S_3O_2 + ^{3}SO → ^{3}SSOSOSO → $^1SO_2$ + ^{3}SSOS reaction profiles**.**	S49
**Supplementary Figure 49.** CCSD/cc-pVDZ reaction profiles for ^{3}SO + ^{1}SSO → $^1SO_2$ + $^3S_2$.	S53
**Supplementary Figure 50.** Spin densities of the O4 atom through a relaxed scan of the O4-S5 bond distance at different levels of theory.	S54
**Supplementary Figure 51.** Comparison of reaction rates computed using abundance profiles from Pinto et al.[1] to plots form Pinto et al.[1]	S57
**Supplementary Figure 52.** Comparison of steady state computed values versus profiles from Pinto et al.[1] for ^{3}SO and the SO dimer.	S57
**Supplementary Figure 53.** Same as Supp. Fig. 52 but for ^1S_2O and $^3S_2$.	S58
**Supplementary Figure 54.** Rates computed for S_2O production reactions.	S59
**Supplementary Figure 55.** Rates computed for S_2O production reactions with multiconfigurational values.	S59

List of Supplementary Tables

**Supplementary Table 1.** Photochemical outcome of the *cis*-OSSO and *trans*-OSSO runs departing from the S_2 state for the MS-CASPT2 NAMD simulations.	S13
**Supplementary Table 2.** Information of the NAMD simulations run at the TD-B3LYP/6-31G* level of theory.	S14
**Supplementary Table 3.** TD-DFT dissociation limits for the $(SO)_2$ studied isomers.	S15
**Supplementary Table 4.** Summary of the number of trajectories per channel and the calculated channel yields for the TD-DFT NAMD runs.	S30
**Supplementary Table 5.** CAS used to compute the reaction profiles (optimizations and final energies) with the (MS)-CASPT2/ANO-L-VTZP method and studied spin multiplicities of the chemical transformations.	S33
**Supplementary Table 6**. MS-CASPT2 activation energies ($\Delta E^{\ddagger}$), energy difference between reactants and products (ΔE), and calculated rates for thermal processes derived from the reactivity between ^{1}OSSO and ^{2}NO / ^{3}O / ^{3}S / ^{2}H, as well as those related to the reaction between ^{2}ClS and ^{3}SO. Energetic profiles of the association of ^{3}S and $^3O_2$ / S are also shown.	S34
**Supplementary Table 7.** Photolysis rates from *cis-/trans*-OSSO to produce SO from Frandsen et al.,[2] with Venusian altitude set to 64 km and latitude 0º and updated value considering the yields of photogenerated SO computed in this work.	S35
**Supplementary Table 8**. Number density (reported and computed here; in molecules cm^{-3}) in the Venusian atmosphere at 64 km altitude of the species involved in the bimolecular reactions studied in this work.	S35
**Supplementary Table 9.** Electronic activation energies ($\Delta E^{\ddagger}$) and energy differences between reactants and products (ΔE) for reactivity derived from the interaction between ^{1}OSSO and ^{3}SO.	S51
**Supplementary Table 10.** Gibbs activation energies ($\Delta G^{\ddagger}$) and Gibbs energy difference between reactants and products (ΔG) for reactivity derived from the interaction between ^{1}OSSO and ^{3}SO.	S51
**Supplementary Table 11.** Expected value of the total spin operator $\langle S^2 \rangle$. The ideal value for a triplet state is 2.	S52
**Supplementary Table 12.** T_1 diagnostic values for coupled-cluster calculations. T_1 values >0.02 suggest the need of multireference electron correlation methods[3] such as CASPT2.	S52
**Supplementary Table 13.** Reactions used in the photochemical steady state model with rate constants obtained from the bibliography or from this work.	S55

1. Supplementary Note 1. Computational details of the MRCI profiles

The excited-state calculations were done in the C_s symmetry group. We have used the complete-active-space self-consistent field (CASSCF) method followed by the internally contracted multi-reference configuration interaction (MRCI) [4,5] scheme to compute the dynamic electron correlation. Here, the atoms were described by aug-cc-pV(T+d)Z basis set. The CASSCF active space was chosen after considering the [1a′-14a′] and [1a″- 2a″] lowest molecular orbitals as doubly occupied and keeping the remaining valence orbitals as active. MRCI calculations included all configurations of the CI expansion of the CASSCF wavefunctions having a weight larger than 0.01. All singlet and triplet electronic states were calculated to the first dissociation limit (SO + SO), *i.e.* four ^{1}A′ and two ^{1}A″ states were averaged in the CASSCF procedure. Only three triplet states were averaged, *i.e.* ^{3}A′ and two ^{3}A″ states. This leads for instance to considering more than 10^8 contracted and $3{\cdot}10^{11}$ uncontracted configuration state function when computing singlet electronic states. The MOLPRO2019 software was used [6].

2. Supplementary Note 2. MS-CASPT2 non-adiabatic molecular dynamics. *Cis* and *trans*-OSSO

2.1 Simulation details

All trajectories were computed making use of the surface-hopping including arbitrary couplings (SHARC) code [7,8]. The electronic structure was computed with the MS-CASPT2 method [9–11] as implemented in the OpenMolcas software package [12], interfaced with the SHARC program. Therefore, wave functions, energies, nuclear and electronic gradients and spin-orbit couplings were calculated on the fly by the OpenMolcas software whereas SHARC computed the diabatic states, the hop probabilities between them, and propagated the excited-state trajectories. Decoherence corrections were included using the energy-based method of Granucci, Persico and Zoccante with the recommended parameter of C = 0.1 a.u. [13] For all systems, the integration of the nuclear motion was done by means of the Velocity-Verlet algorithm using a time step of 0.5 fs.

Initial conditions were obtained stochastically at 300 K by sampling the ground state minimum *via* a Wigner distribution employing CASPT2 frequencies. The atomic coordinates and velocities were subsequently used to start the molecular dynamic simulations. For *cis*-OSSO, 30 trajectories departed from the S_1 state and 86 from S_2, whereas for *trans*-OSSO 70 runs started from S_2. The S_1 state is mostly dark and unreactive. The dynamics were run on diagonal potential energy surfaces (PESs) resulting from the diagonalization of the Hamiltonian containing non-adiabatic couplings and spin-orbit couplings [14], employing the SHARC algorithm for surface hopping. Globally, the total simulation time was 140 fs, even though some trajectories were extended to further simulation times to confirm certain photoproducts. A total of 4 singlet and 4 triplet spin-free (SF) states were mixed to obtain the corresponding SO states, making use of the state-average CASSCF method and employing the CAS(12,10) active space shown in Supplementary Figure 1. All states were active in the simulations except the fourth triplet root, which was excluded in all cases to save computational time. The MS-CASPT2 energies and gradients were computed with the IPEA shift set to 0.25 a.u. [15],

whereas the imaginary level shift was set to 0.2 a.u. to minimize the presence of weakly-interacting intruder states [16]. The ANO-S-VDZP basis set was used for all MS-CASPT2 dynamic runs.

A certain number of trajectories were excluded from the final analyses (100 trajectories were prepared in the initial setup for the simulations starting from S_2) due to premature crashes caused mostly by errors in the calculation of the MS-CASPT2 numerical gradients, problems with SOC determinations, divergence in CASSCF iterations, or inconsistencies in the total energy (variations larger than 0.5 eV between consecutive steps).

As previously used in the literature [17,18], bonds are considered as broken when they are stretched 1.5 times the bond distance at the Franck-Condon geometry (optimized at the same level of theory as the dynamics). These values are indicated with grey solid lines in the distance plots (also for the TD-DFT NAMD simulations, see below). This criterion is again confirmed by the fact that, considering the significant bond stretching momentum observed in the dissociations, as shown in the corresponding interatomic distance curves, and the fact that for all bonds considered in this work, no trajectory reduces the bond distance after passing the 1.5 times stretch limit (see Supplementary Figures 6, 7, and 9). The numbers 1-4 of the atom labelling are arbitrary and only depend on the total number of atoms, and do not refer to the total number of atoms of the same element. The product analysis of the whole sets of excited-state trajectories requires the inspection of the evolution of the most relevant S-S, O-O, and S-O distances over time, even if there is no covalent bonding in the ground-state, to analyze the possible occurrence of all possible photoisomerizations or other events.

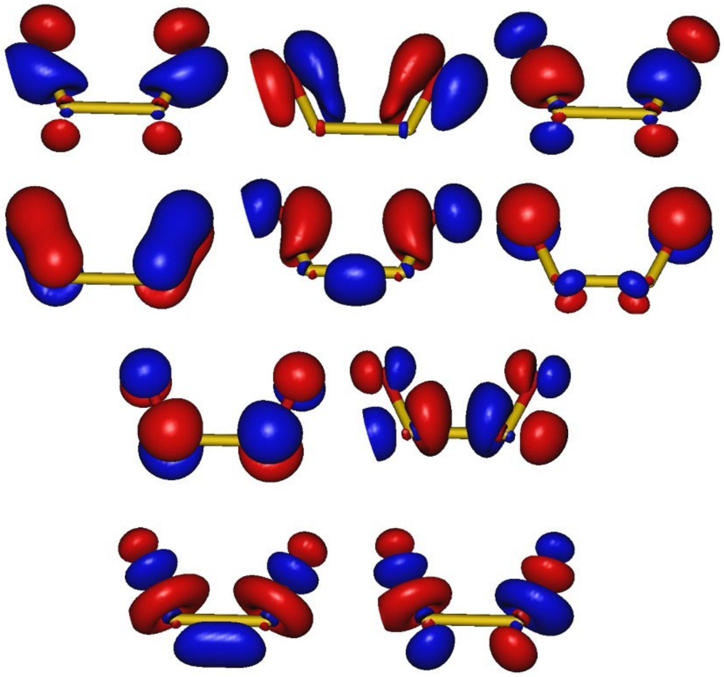

Supplementary Figure 1. SA(4)-CASSCF natural orbitals employed in the MS-CASPT2 NAMD simulations for *cis*-OSSO. An analogous active space has been used for the simulation on the *trans* isomer.

2.2 Method validation

The MS-CASPT2/ANO-S-VDZP method detailed in the previous section has been validated by comparing the energy profiles along the rigid scans of the S-S and O-S bonds for both *cis*- and *trans*-OSSO, computed at this level of theory and with the MRCI method (Supplementary Figures 2-5). Overall, MS-CASPT2 reproduces the main features of the S_0-S_2 and T_1-T_3 curves with maximum differences of a few tenths of eV. Discrepancies between both electronic structure methods increase for the T_1 and T_2 curves at large S-S distances, however, the population of triplet states is clearly negligible and thus these differences will not influence the dynamics. In addition, at these regions, the molecule is already dissociated and therefore the mentioned small changes in energy do not affect the branching into the different photoproducts. Globally, these results fully validate the use of the MS-CASPT2/ANO-S-VDZP method as a suitable level of theory to run the NAMD simulations for both OSSO isomers.

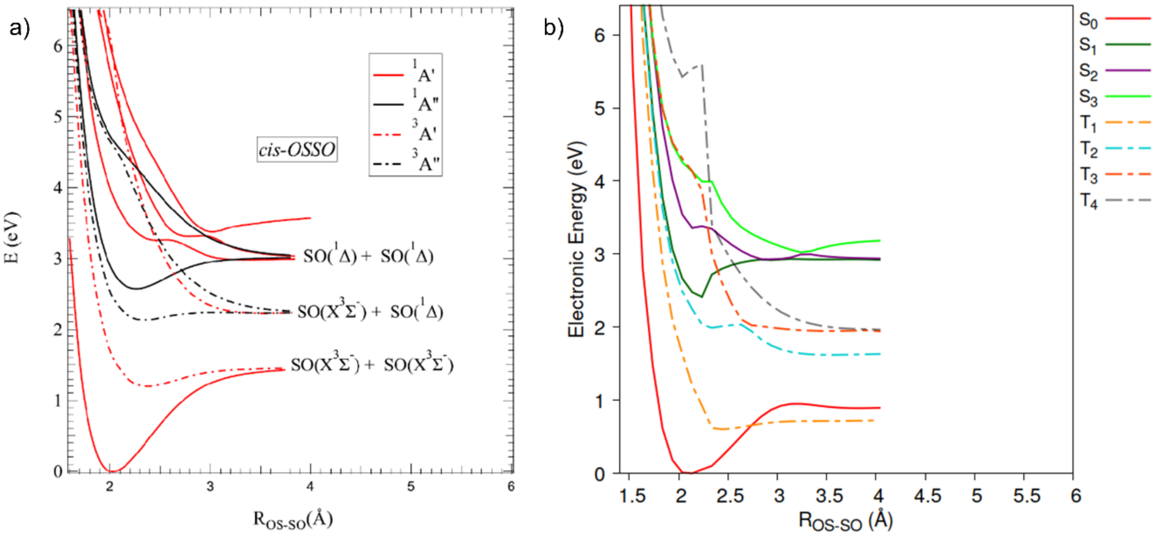

Supplementary Figure 2. MRCI+Q/aug-cc-pV(T+d)Z (a) and MS-CASPT2/ANO-S-VDZP (b) potential energy curves of the low-lying singlet and triplet electronic states of *cis*-OSSO along the rigid scan of the OS-SO bond distance.

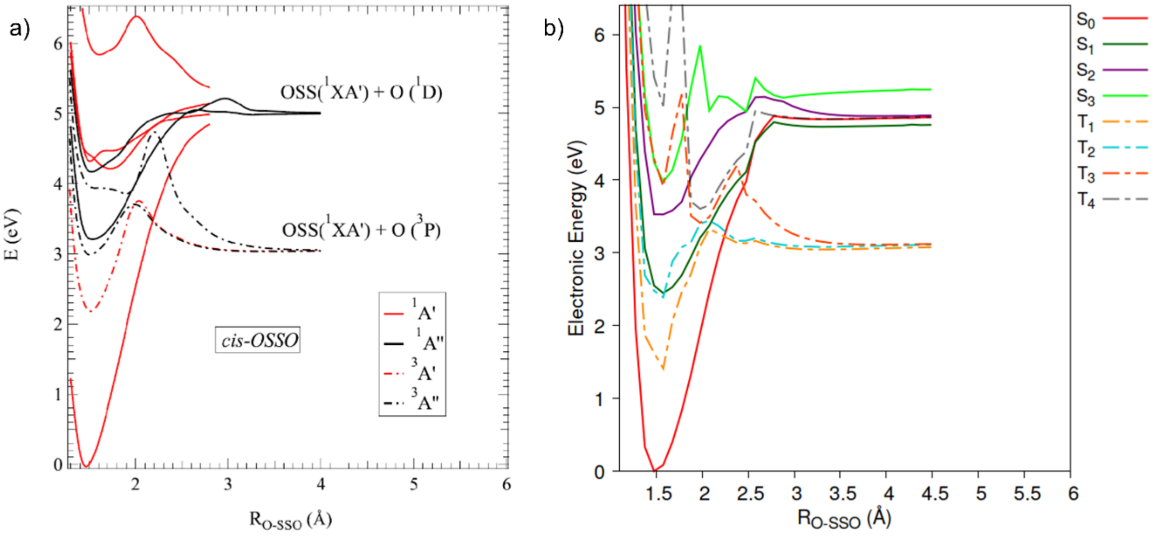

Supplementary Figure 3. MRCI+Q/aug-cc-pV(T+d)Z (a) and MS-CASPT2/ANO-S-VDZP (b) potential energy curves of the low-lying singlet and triplet electronic states of *cis*-OSSO along the rigid scan of the O-SSO bond distance.

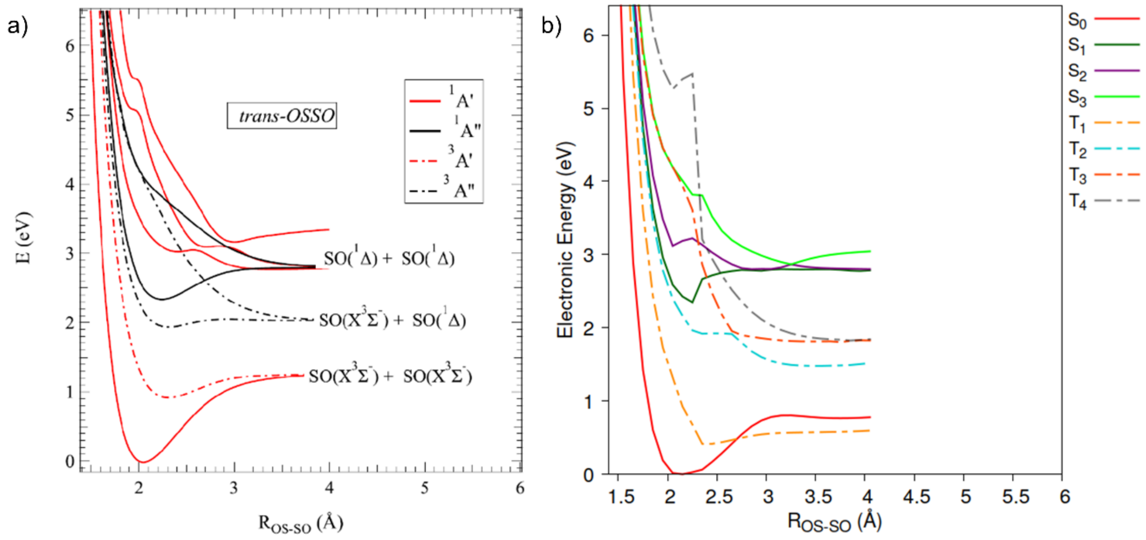

Supplementary Figure 4. MRCI+Q/aug-cc-pV(T+d)Z (a) and MS-CASPT2/ANO-S-VDZP (b) potential energy curves of the low-lying singlet and triplet electronic states of *trans*-OSSO along the rigid scan of the OS-SO bond distance.

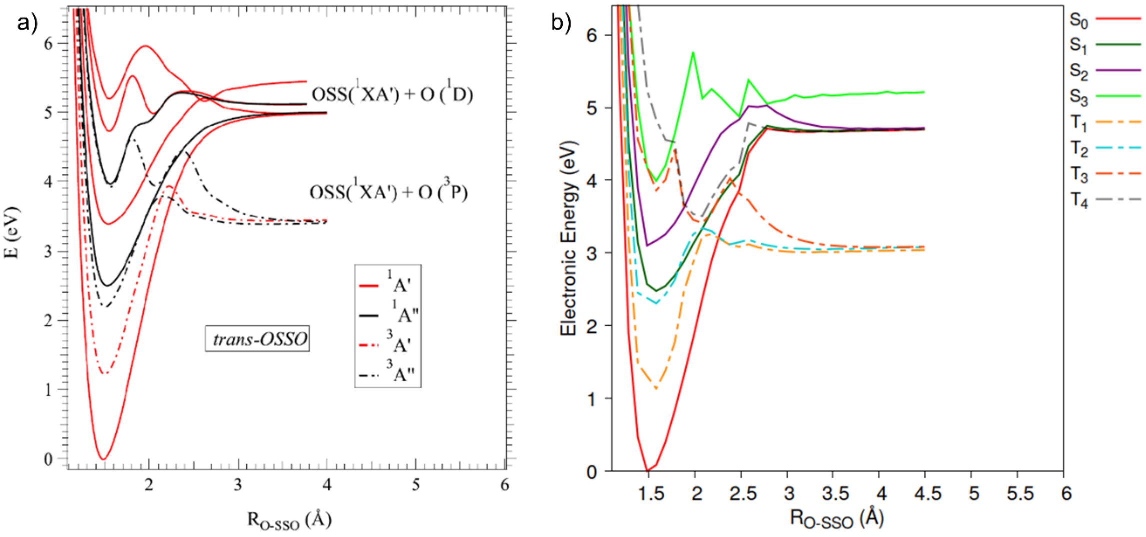

Supplementary Figure 5. MRCI+Q/aug-cc-pV(T+d)Z (a) and MS-CASPT2/ANO-S-VDZP (b) potential energy curves of the low-lying singlet and triplet electronic states of *trans*-OSSO along the rigid scan of the OS-SO bond distance.

2.3 Results

For *cis*-OSSO and *trans*-OSSO, two excited states were initially populated, namely the dark S_1 and the bright S_2 states. Results are compiled in Supplementary Figures 6-10 and Supplementary Table 1. Whereas no photoreaction was observed when starting the simulations from the S_1 state (Supplementary Figure 6), time evolution of the S-S and S-O bond distances upon excitation to the S_2 state (Supplementary Figures 7 and 9) indicate that SO + SO is the only photodissociation product. For *cis*-OSSO, about 60% of the S_2 population decays into S_1 before 40 fs, while the same phenomenon takes place in *trans*-OSSO at about 80 fs. The triplet state participation is almost zero. In both isomers the S-S photolysis takes place in the S_2 and S_1 states, the ground state only starts to be marginally populated (about 15%) after 80-100 fs. The SO fragments are thus released in the singlet manifold (S_2, S_1, and S_0 states). Those excited ^1SO fragments are assumed to decay finally to their respective triplet ground states ^3SO. To quantify the $^1SO/^3SO$ ratio, photodynamics should be extended far beyond the time limit of the current simulations, which is prohibitive at the level of theory required for a correct description. This is out of the scope of this work. Nevertheless, future implementations of machine learning techniques together with the photodynamics methodology shall help to quantify this ratio and the relevance of further reactivity of ^1SO.

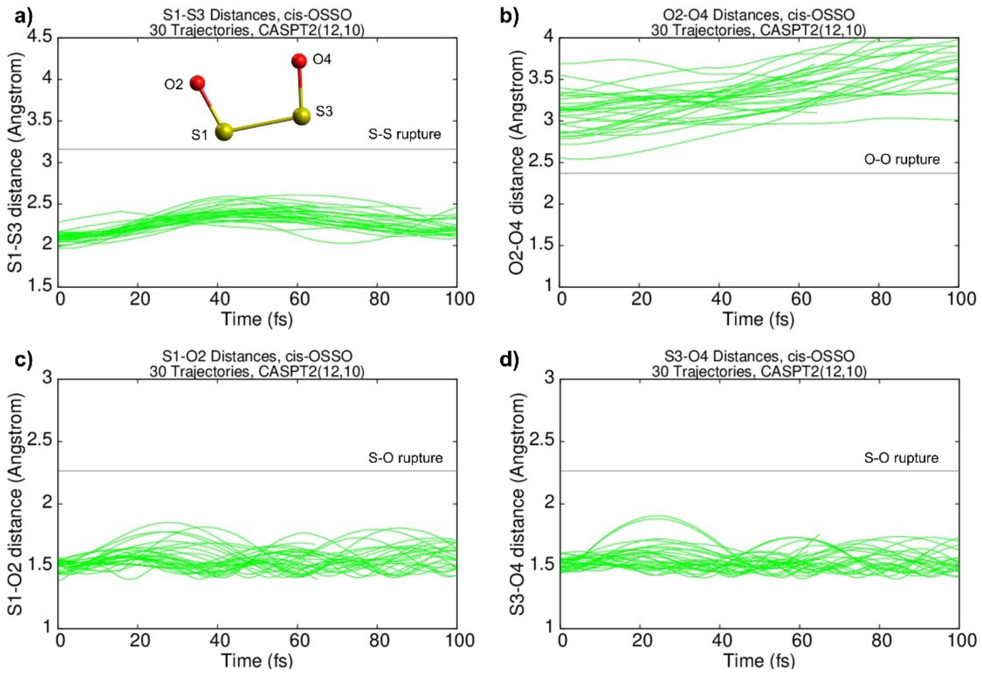

Supplementary Figure 6. Time evolution of the most relevant bond distances of *cis*-OSSO upon excitation to the S_1 state. No photoreaction is observed in the first 100 fs after light absorption.

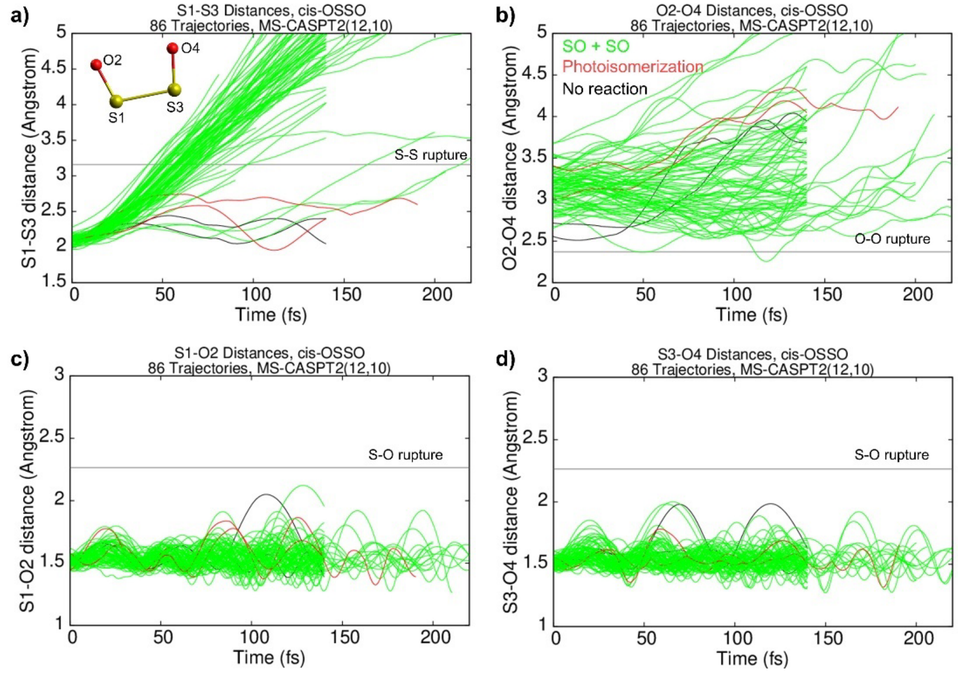

Supplementary Figure 7. Time evolution of the most relevant bond distances of *cis*-OSSO upon excitation to the S_2 state.

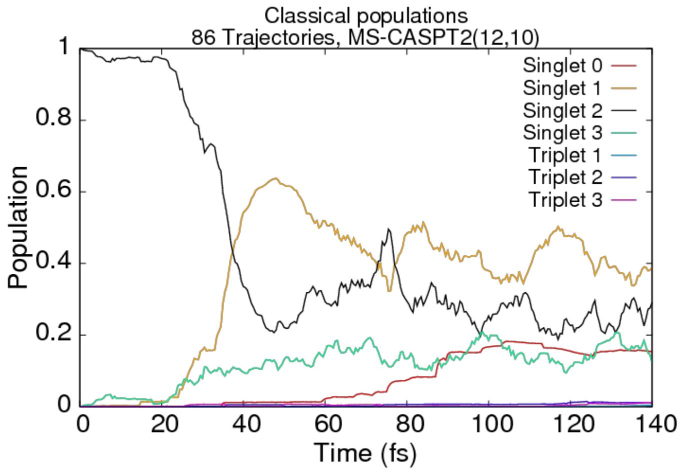

Supplementary Figure 8. Normalized populations of the 86 trajectories ensemble starting from S_2 for *cis*-OSSO.

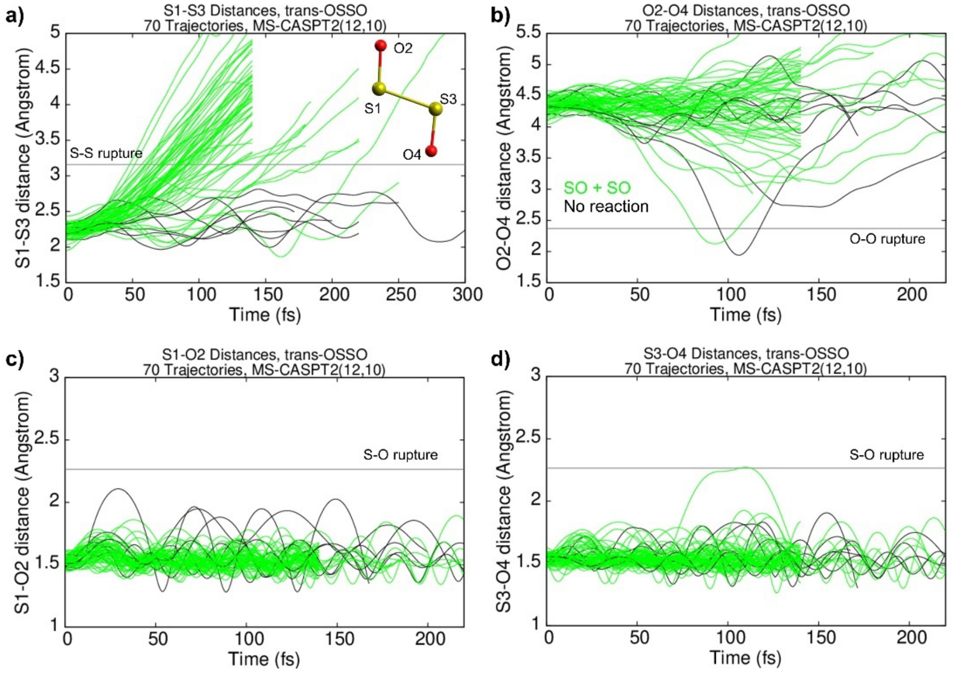

Supplementary Figure 9. Time evolution of the most relevant bond distances of *trans*-OSSO upon excitation to the S_2 state.

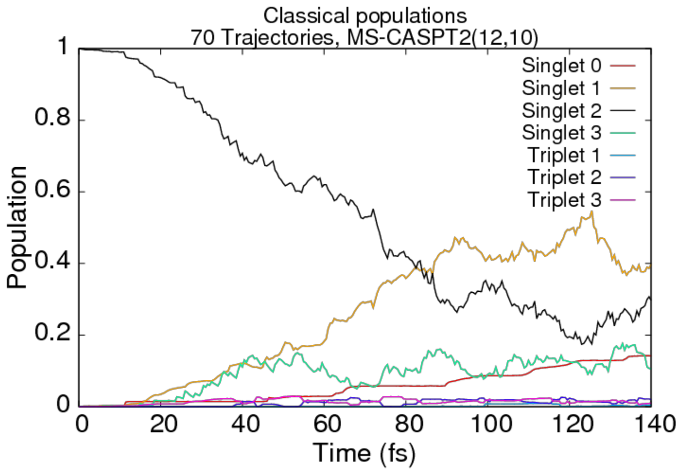

Supplementary Figure 10. Normalized populations of the 70 trajectories ensemble starting from S_2 for *trans*-OSSO.

Supplementary Table 1. Photochemical outcome of the *cis*-OSSO and *trans*-OSSO runs departing from the S_2 state for the MS-CASPT2 NAMD simulations.

**Photochemical channel**	**# Traj.**	**%**
	cis-OSSO	
cis-OSSO → ^{1}SO + ^{1}SO	82	95
No photoreaction	4	5
**Total**	86	100
	trans-OSSO	
trans-OSSO → ^{1}SO + ^{1}SO	63	90
No photoreaction	7	10
**Total**	70	100

3. Supplementary Note 3. TD-DFT non-adiabatic molecular dynamics

3.1 Simulation details

The simulation protocol for the TD-DFT[19,20] non-adiabatic dynamics is analogous to the one used for the MS-CASPT2 simulations, only the differences between them are mentioned here. Electronic energies and analytical gradients are computed at the TD-B3LYP/6-31G* level [21,22] with the Gaussian 09 program [23], whereas SHARC computed the diabatic states, the hop probabilities between them, and propagated the excited-state trajectories. The ground state was computed using a restricted DFT ansatz, and the quadratic convergence was used when default self-consistent field procedure did not converge. No triplet states were included in the simulations since its participation is excluded from the MS-CASPT2 simulations.

Initial conditions were generated with a Wigner distribution as commented before, however, the population of the initial state was decided through a different procedure. We performed single-point calculations with the TD-B3LYP/6-31G* method on top of each initial condition to obtain a set of vertical absorption energies and oscillator strengths. Later, the initial active state was flagged stochastically, as documented elsewhere [24], simulating an excitation window that varied according to the optical properties of the different isomers. Supplementary Table 2 collects the excitation windows, number of trajectories, states included in the simulations, initial active states, and total simulation time for the NAMD at the TD-DFT level. The simulations were run only for 80 fs, since it is sufficient time to observe the photodissociations (and the mutual competition between the different breaking channels) and the population of the ground state (not well accounted in the TD-B3LYP NAMD, see next section) is negligible, as shown by the population analysis of the MS-CASPT2 simulations (Supplementary Figures 8 and 10).

Supplementary Table 2. Information of the NAMD simulations run at the TD-B3LYP/6-31G* level of theory.

System	Excitation window (eV)	# of states (including S_0) included in the simulation	# of trajectories and initial active states (within parenthesis)	Total # of trajectories	Total simulation time (fs)[a]
cis-OSSO	2.5-4.0	4	183 (S_2), 3 (S_3)	186	80
trans-OSSO	2.5-4.0	4	136 (S_2)	136	80
cyclic OS(=O)S	2.5-5.5	8	4 (S_1), 28 (S_3), 32 (S_4), 66 (S_5), 6 (S_6)	136	140
$S{=}SO_2$	2.5-5.5	6	15 (S_2), 41 (S_3), 75 (S_4), 31 (S_5)	162	80
cis-OSOS	2.5-4.0	6	172 (S_2), 9 (S_3), 4 (S_4), 5 (S_5)	190	80
trans-OSOS	2.0-4.0	6	178 (S_2), 5 (S_3), 5 (S_4), 6 (S_5)	194	80

[a] Some additional simulation time was necessary to verify the photoproducts in some trajectories.

3.2 Method validation

This section explains in detail the validation of the NAMD dynamics performed at the TD-B3LYP/6-31G* level. This level of theory has been systematically benchmarked against MS-CASPT2 dynamics and static profiles to ensure the full reliability of the obtained results.

In bonded structures (*i.e.* when the molecule is not fragmented), results clearly show that the TD-B3LYP/6-31G* method accurately reproduces the excited state energy ordering and associated oscillator strengths as compared to MS-CASPT2(12,10)/ANO-S-VTZP determinations. This is clearly shown for several $^1(SO)_2$ isomers in Supplementary Figures 11-16. Therefore, there is a range of bond distances, including regions close to the Franck-Condon area and zones with relatively long bond lengths (see below), in which the TD-B3LYP/6-31G* description is fully reliable. Since the branching ratio into different photodissociation channels is largely dominated by the initial coordinates and velocities and the nature of the initial active state, and considering that the ultrafast dissociations take place at the sub-100 fs regime, where the ground state is not active at all, this level

of theory provides the correct photoproduct distribution for each system, which is the main goal of this study. As a matter of fact, TD-DFT dynamics correctly identify the $^3SO + {}^3SO$ dissociation as the only photoproduct (100%) of *cis*- and *trans*-OSSO, in reasonable agreement with the MS-CASPT2 dynamics (≥90%).

On the other hand, it is well known that the ground-state profiles computed with the restricted DFT ansatz are not reliable at bond dissociation limits because a single restricted determinant cannot account for both zwitterionic and diradical solutions (see top panels of Supplementary Figures 11-16). Thus, these methods typically show an artificial energy increase as long as the bond distance elongates, whereas multiconfigurational schemes such as MRCI or MS-CASPT2 provide the correct asymptotic, smooth dissociation profiles. Thus, the bond distance at which the energy starts to increase artificially, can be easily found by comparing the TD-B3LYP vs MS-CASPT2 profiles. This has been systematically evaluated for all systems considered in this work in Supplementary Figures 11-16. By inspection of these results, we can clearly see that, for *cis*-OSSO, the TD-DFT profiles exhibit an artificial minimum at a S1-S3 distance of ~2.8 Å followed by a significant energy increase upon larger interatomic distances, whereas the MS-CASPT2 profiles are more planar (Supplementary Figure 11). Therefore, this minimum can artificially trap some trajectories in this area that would have certainly dissociated at the MS-CASPT2 level, as unambiguously observed in the MS-CASPT2 dynamics (Supplementary Figure 7). The reason is that once reached these S1-S3 bond distances, the molecules always dissociate. Therefore, it is reasonable to conclude that the runs that reach this area must be considered as dissociated. Taking into account that the description at shorter distances is trustworthy (as shown by Supplementary Figure 11), it can be safely concluded that the dissociation limit for the S1-S3 bond at the TD-DFT level is 2.8 Å. This value is indicated with a thicker black line in the corresponding figures of the next subsection.

The same analysis can be performed for the rest of systems and bonds. Supplementary Table 3 summarizes the TD-DFT bond dissociation limits determined by comparison with the MS-CASPT2 profiles.

Supplementary Table 3. TD-DFT dissociation limits for the $(SO)_2$ studied isomers.

System	Bond	Photoproduct	TD-DFT dissociation limit / Å	Normal dissociation criterion (1.5 times the FC distance) / Å
cis/*trans*-OSSO	S1-S3	$^3SO + {}^3SO$	~2.8	3.16
cis/*trans*-OSSO	S1-O2	$^3S + {}^1SO_2$ (not observed)	Not affected	2.26
$S{=}SO_2$	S1-S3	$^3S + {}^1SO_2$	~2.7	3.16
cyclic-OS(=O)S	S1-S3	*cis*/*trans*-OSOS	Not affected	3.16
cis/*trans*-OSOS	S3-O2	$^3SO + {}^3SO$	~2.2	2.26
cis/*trans*-OSOS	S1-O2	$^3S + {}^1SO_2$	~2.2	2.26

Finally, in order to further test the TD-B3LYP NAMD validity, we have computed the potential energy landscape along a *cyclic*-OS(=O)S → S + SO_2 trajectory with both the TD-B3LYP/6-31G* and the MS-CASPT2(12,10)/ANO-S-VTZP methods. Results are shown in Supplementary Figure 17. The agreement between both profiles is very good, especially at the beginning of the run.

Three important facts emerge from all MS-CASPT2 and TD-DFT analyses presented above:

- The MS-CASPT2 dynamics indicate that neither the ground state nor the triplet states participate in the photodissociations of the *cis* and *trans*-OSSO.
- In TD-B3LYP NAMD, hops to the ground state are almost shut down due to the poor coupling between the ground state (single determinant) and the excited states (CI expansion of single excitations and de-excitations) due to the TD-DFT formalism. Therefore, artificial trapping in ground/excited-state degeneracies (not happening at the MS-CASPT2 level, in which both ground and excited states are treated on equal foot and normal hopping is allowed), are not a problem.
- The dissociation bond distances can be easily corrected by comparing both TD-B3LYP/6-31G* vs MS-CASPT2/ANO-S-VTZP energy profiles.

The following reasons justify the use of the TD-B3LYP/6-31G* method to describe the photodynamics of the $^1(SO)_2$ isomers. Note that other molecules not studied here would require the corresponding validation studies.

- The description of the most relevant excited states and oscillator strengths at the TD-B3LYP/6-31G* method is excellent at distances shorter than the dissociation limits, ensuring the correct branching into the different photodissociation channels and therefore allowing correct quantifications of the photoproducts.
- TD-DFT NAMD on *cis-/trans*-OSSO perfectly identifies the main dissociation channel ($^3SO + {}^3SO$) and provides comparable quantifications of the photoproducts (≥90% at the MS-CASPT2 level *vs* 100% with the TD-B3LYP method).
- Analysis of the potential energy landscape of a *cyclic*-OS(=O)S → $^3S + {}^1SO_2$ trajectory evidences small differences between both methods in an actual run.

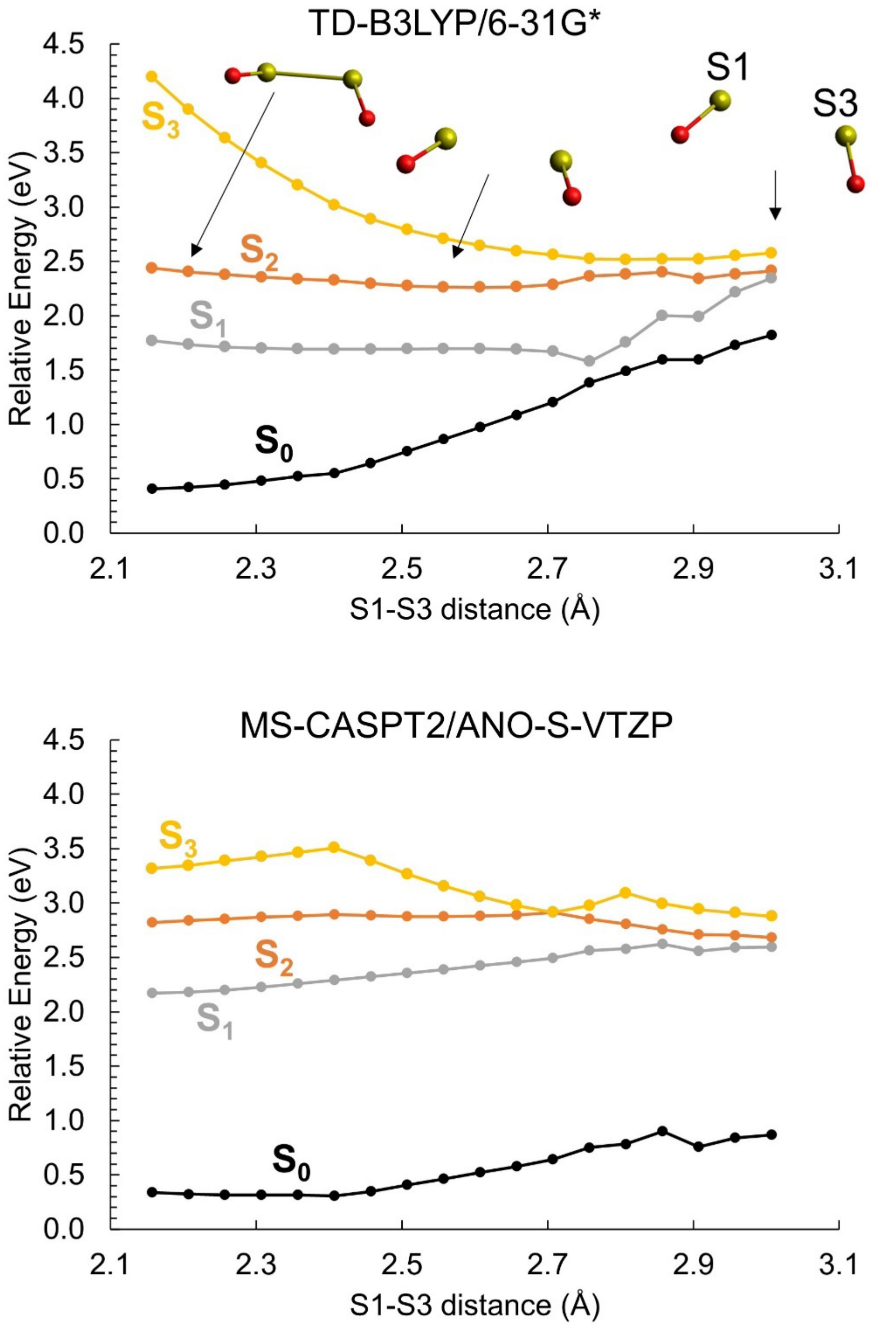

Supplementary Figure 11. TD-B3LYP/6-31G* (top panel) and MS-CASPT2/ANO-S-VTZP (bottom panel) potential energy landscapes for the S1-S3 bond breaking of *cis*-OSSO. Structures have been optimized at the TD-B3LYP/6-31G* level of theory relaxing the S_2 state. Similar results should apply for *trans*-OSSO.

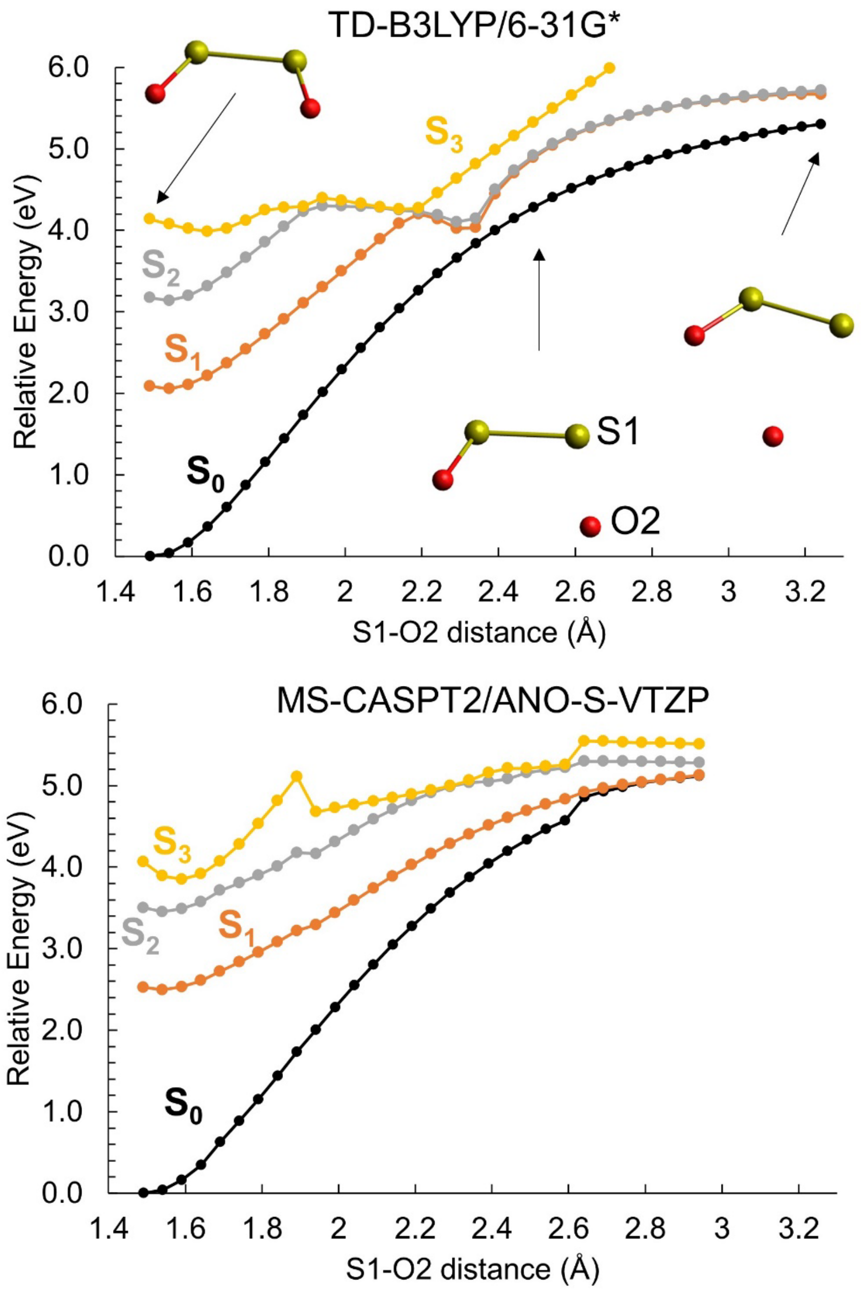

Supplementary Figure 12. TD-B3LYP/6-31G* (top panel) and MS-CASPT2/ANO-S-VTZP (bottom panel) potential energy landscapes for the S1-O2 bond breaking of *cis*-OSSO. Structures have been optimized at the B3LYP/6-31G* level of theory relaxing the S_0 state. Similar results should apply for *trans*-OSSO.

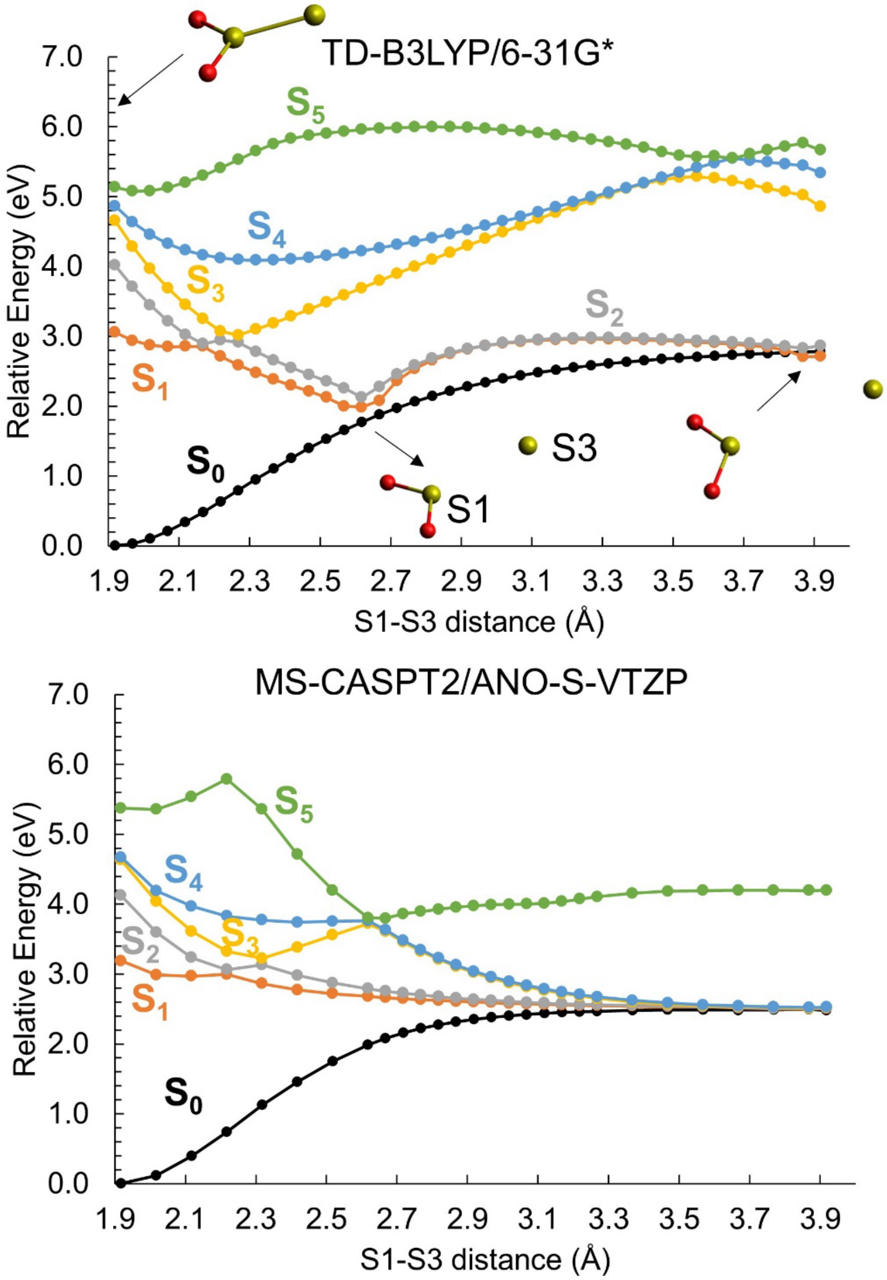

Supplementary Figure 13. TD-B3LYP/6-31G* (top panel) and MS-CASPT2/ANO-S-VTZP (bottom panel) potential energy landscapes for the S1-S3 bond breaking of the trigonal S=SO_2. Structures have been optimized at the B3LYP/6-31G* level of theory relaxing the S_0 state.

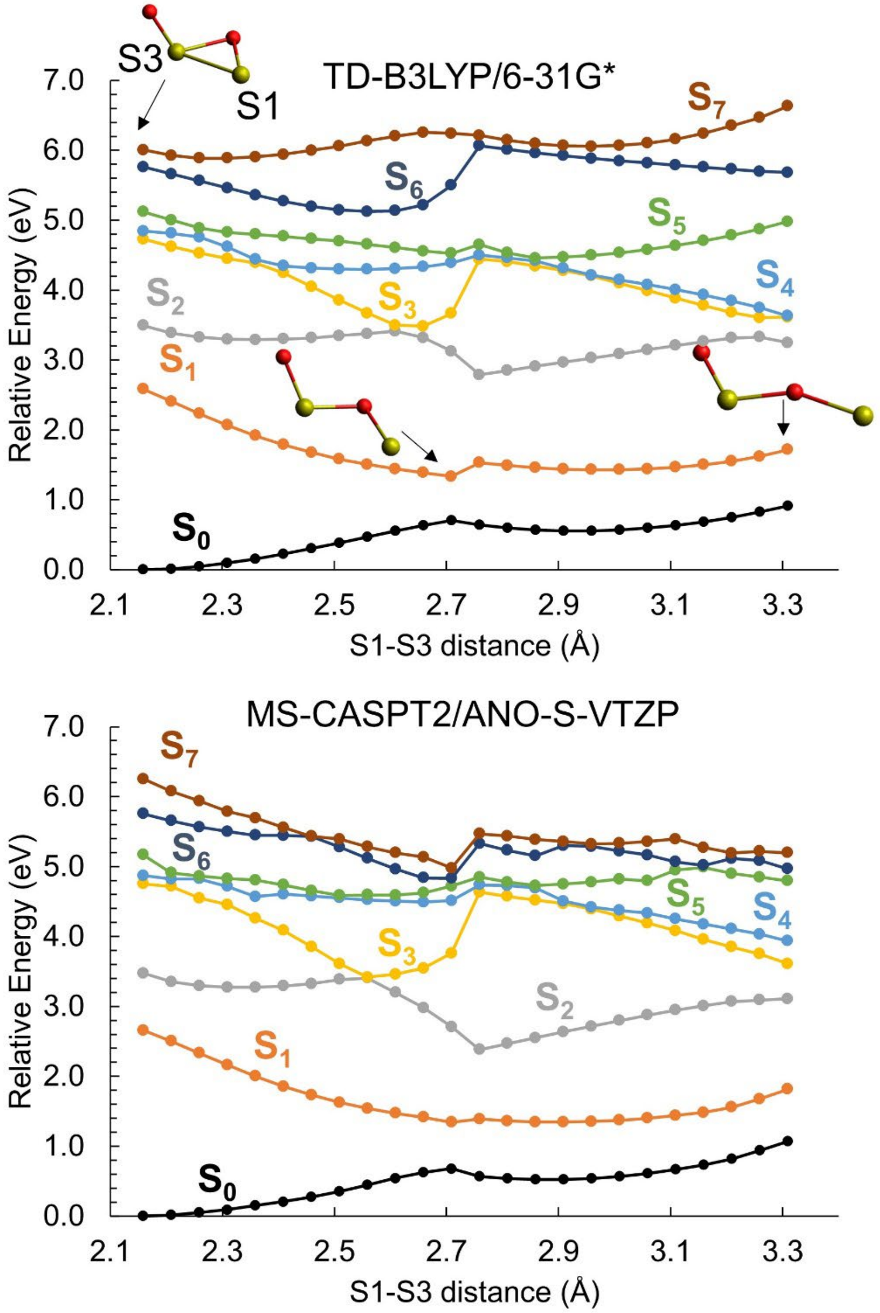

Supplementary Figure 14. TD-B3LYP/6-31G* (top panel) and MS-CASPT2/ANO-S-VTZP (bottom panel) potential energy landscapes for the S1-S3 bond breaking of *cyclic* OS(=O)S. Structures have been optimized at the B3LYP/6-31G* level of theory relaxing the S_0 state.

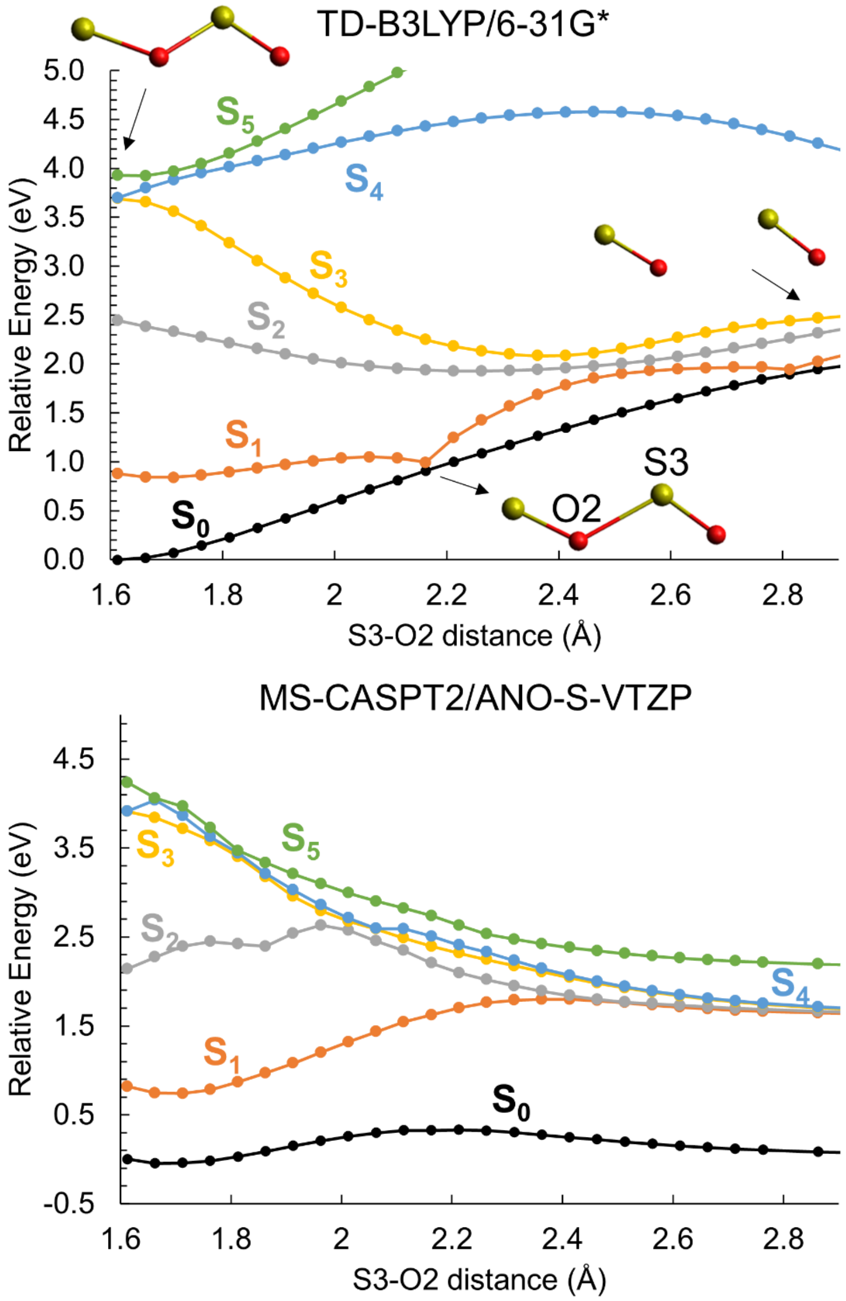

Supplementary Figure 15. TD-B3LYP/6-31G* (top panel) and MS-CASPT2/ANO-S-VTZP (bottom panel) potential energy landscapes for the S3-O2 bond breaking of *trans*-OSOS. Structures have been optimized at the B3LYP/6-31G* level of theory relaxing the S_0 state. Similar results should apply for *cis*-SOSO.

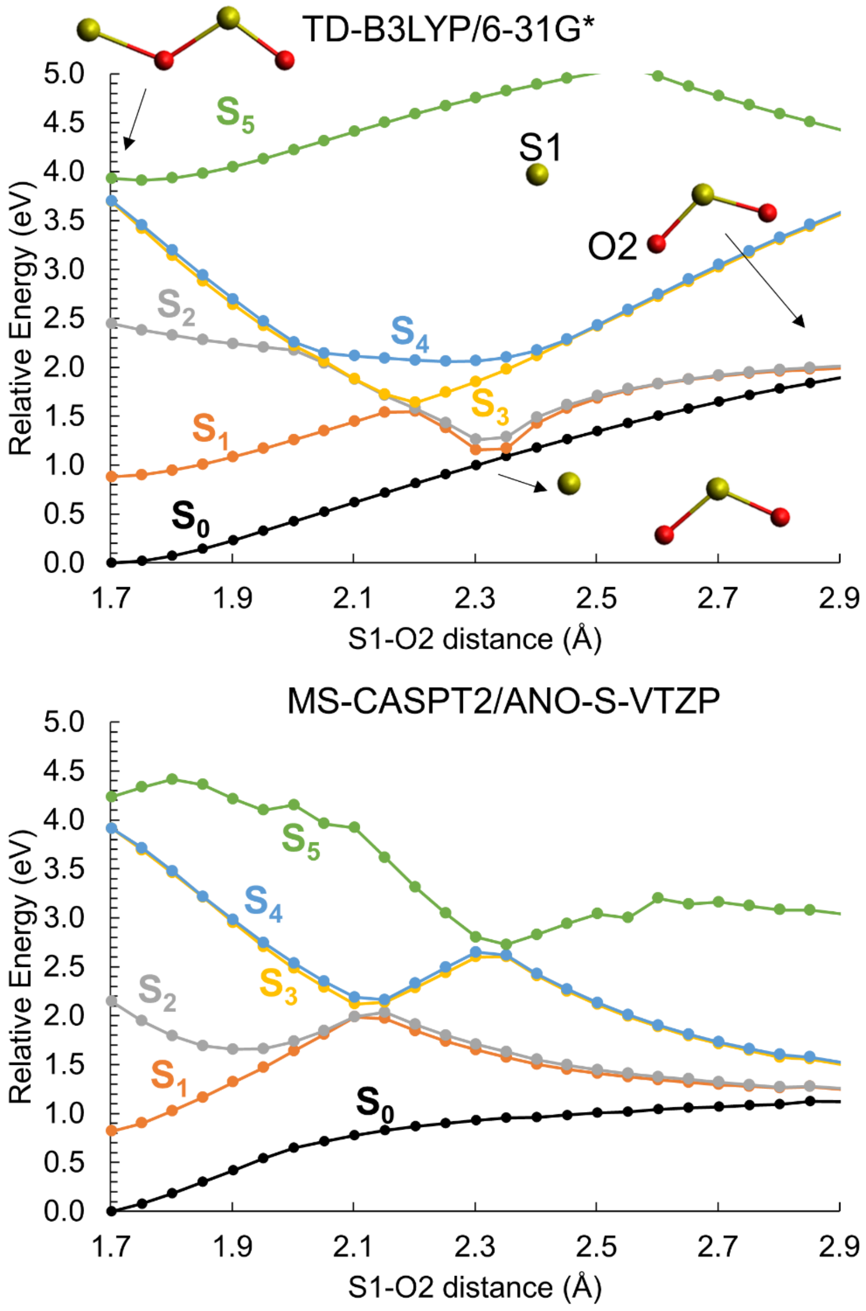

Supplementary Figure 16. TD-B3LYP/6-31G* (top panel) and MS-CASPT2/ANO-S-VTZP (bottom panel) potential energy landscapes for the S1-O2 bond breaking of *trans*-OSOS. Structures have been optimized at the B3LYP/6-31G* level of theory relaxing the S_0 state. Similar results should apply for *cis*-SOSO.

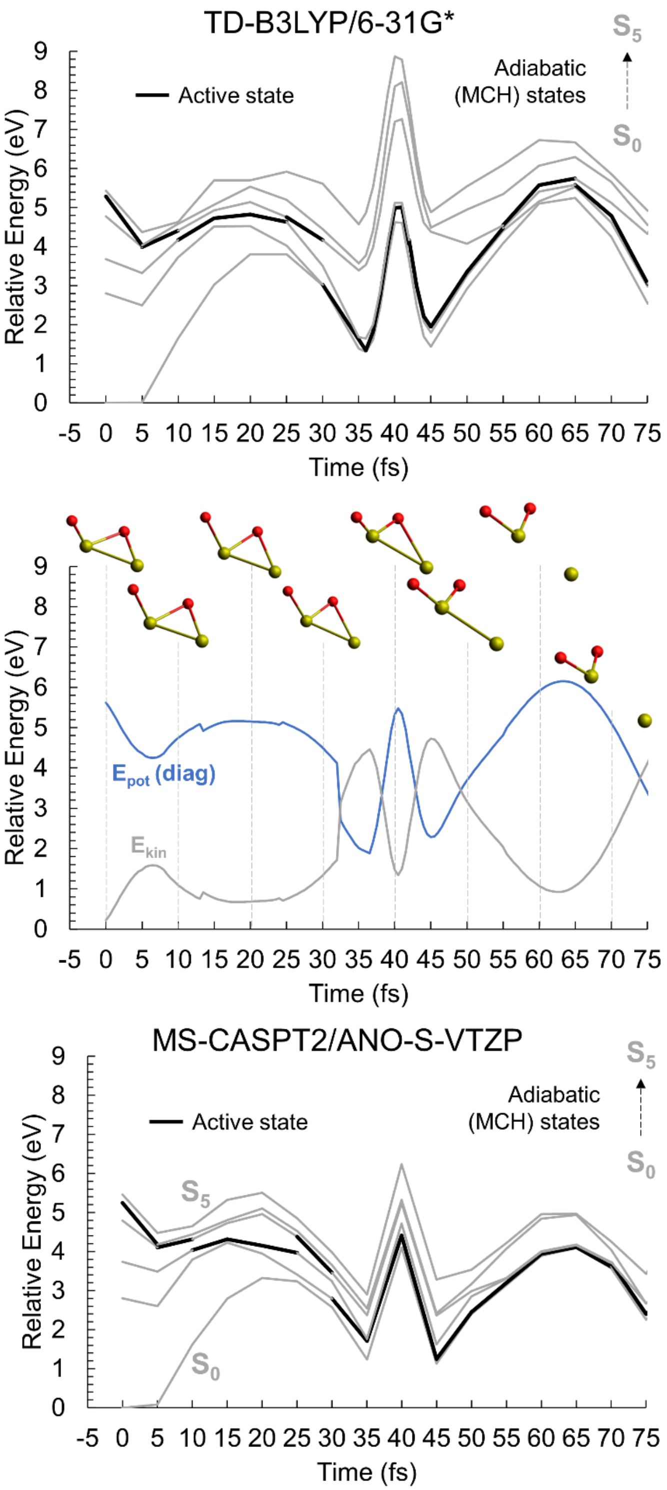

Supplementary Figure 17. TD-B3LYP/6-31G* (top panel), potential (in the diagonal representation) and kinetic energy (mid panel), and MS-CASPT2/ANO-S-VTZP (bottom panel) potential energy landscapes for a *cyclic* OS(=O)S → S + SO_2 trajectory run with the TD-B3LYP/6-31G* NAMD. Several frames of the run are shown at the mid panel.

3.3 Results

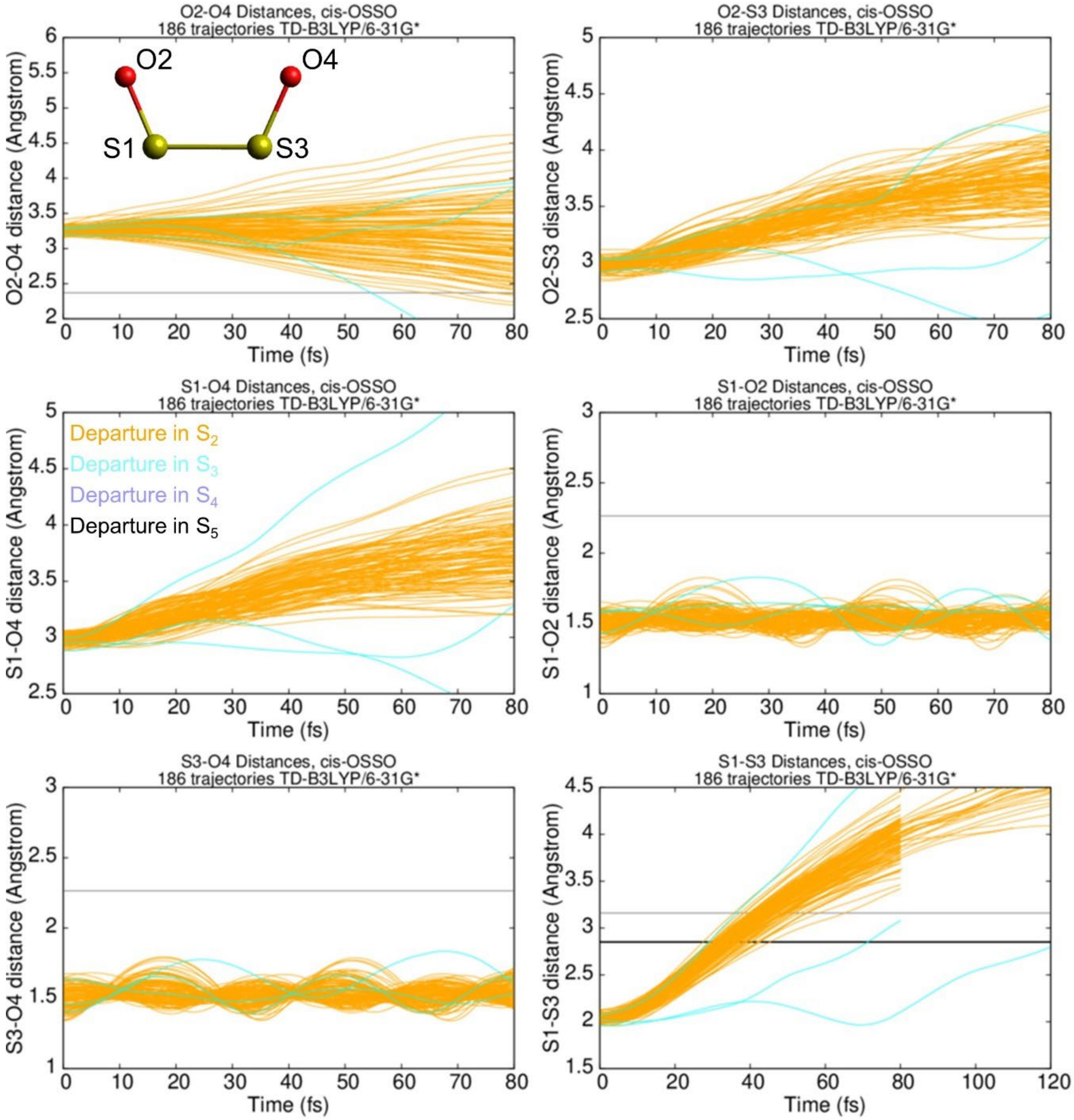

Supplementary Figure 18. Time evolution of the different bond distances for the *cis*-OSSO system computed with the TD-B3LYP/6-31G* NAMD. The horizontal grey bar shows the normal dissociation limit (1.5 times the bond distance at the FC region) and the black bar indicates the TD-DFT dissociation value (verified with MS-CASPT2).

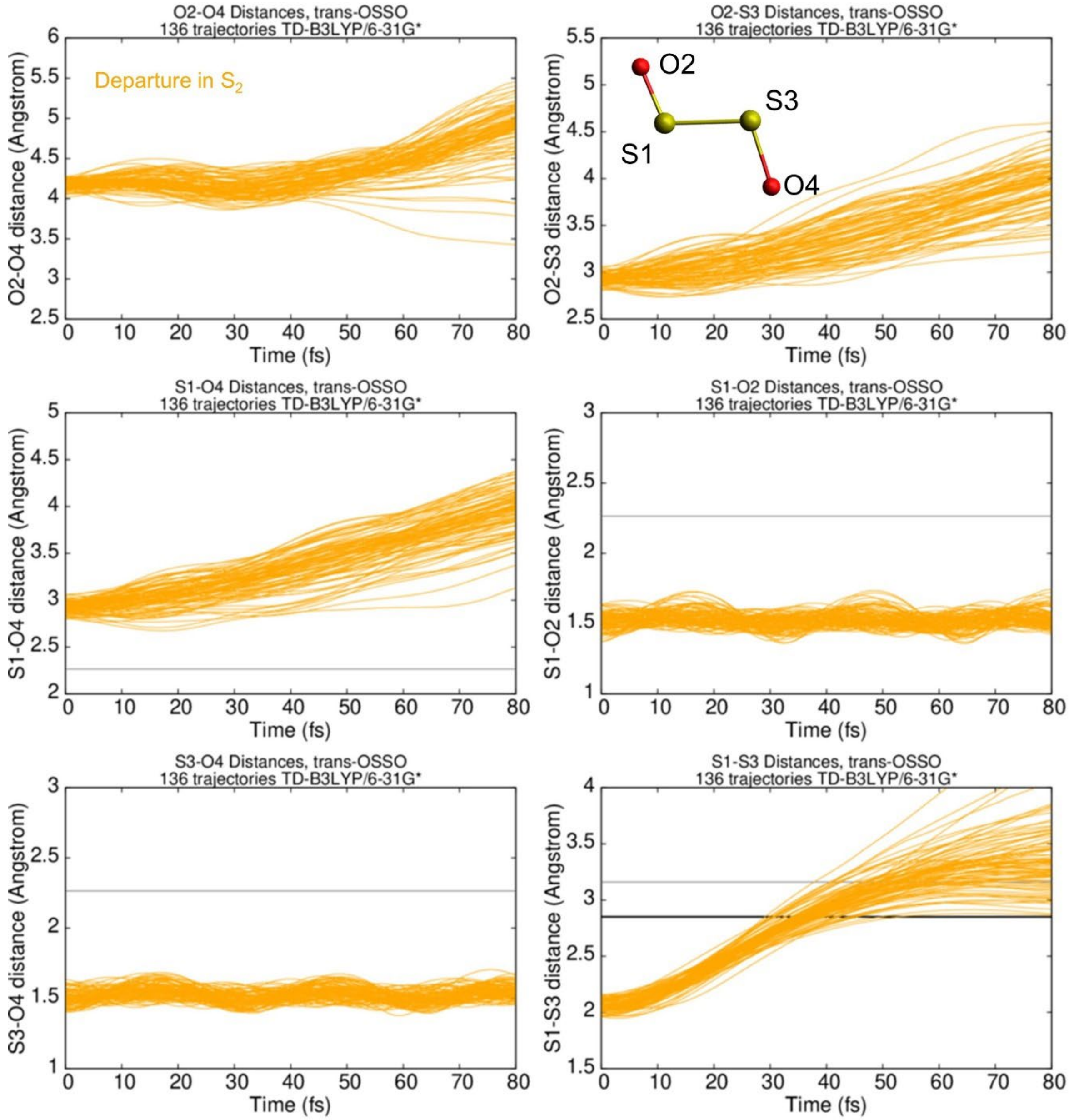

Supplementary Figure 19. Time evolution of the different bond distances for the *trans*-OSSO system computed with the TD-B3LYP/6-31G* NAMD. The horizontal grey bar shows the normal dissociation limit (1.5 times the bond distance at the FC region) and the black bar indicates the TD-DFT dissociation value (verified with MS-CASPT2).

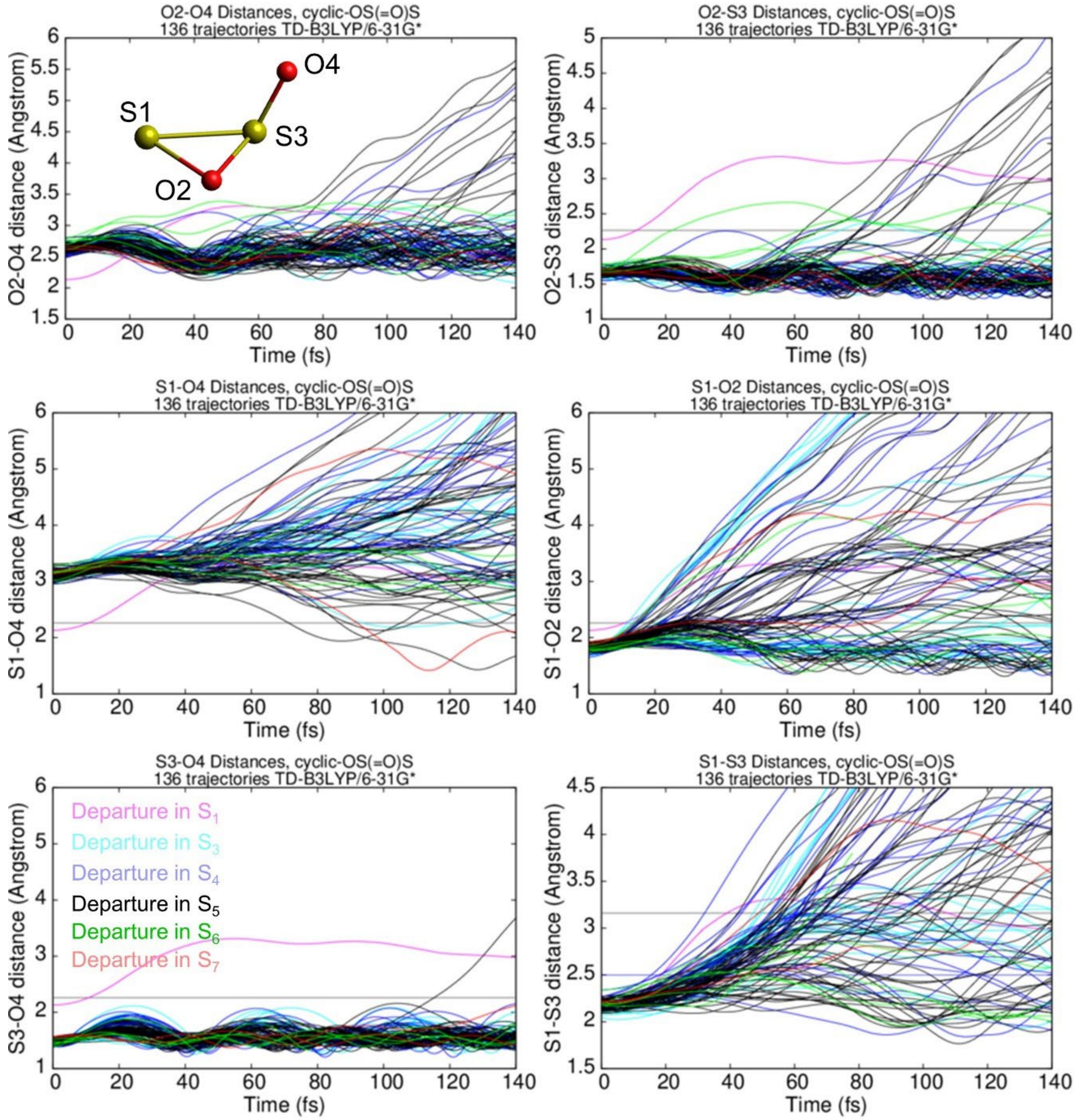

Supplementary Figure 20. Time evolution of the different bond distances for the *cyclic*-OS(=O)S system computed with the TD-B3LYP/6-31G* NAMD. The horizontal grey bar shows the normal dissociation limit (1.5 times the bond distance at the FC region).

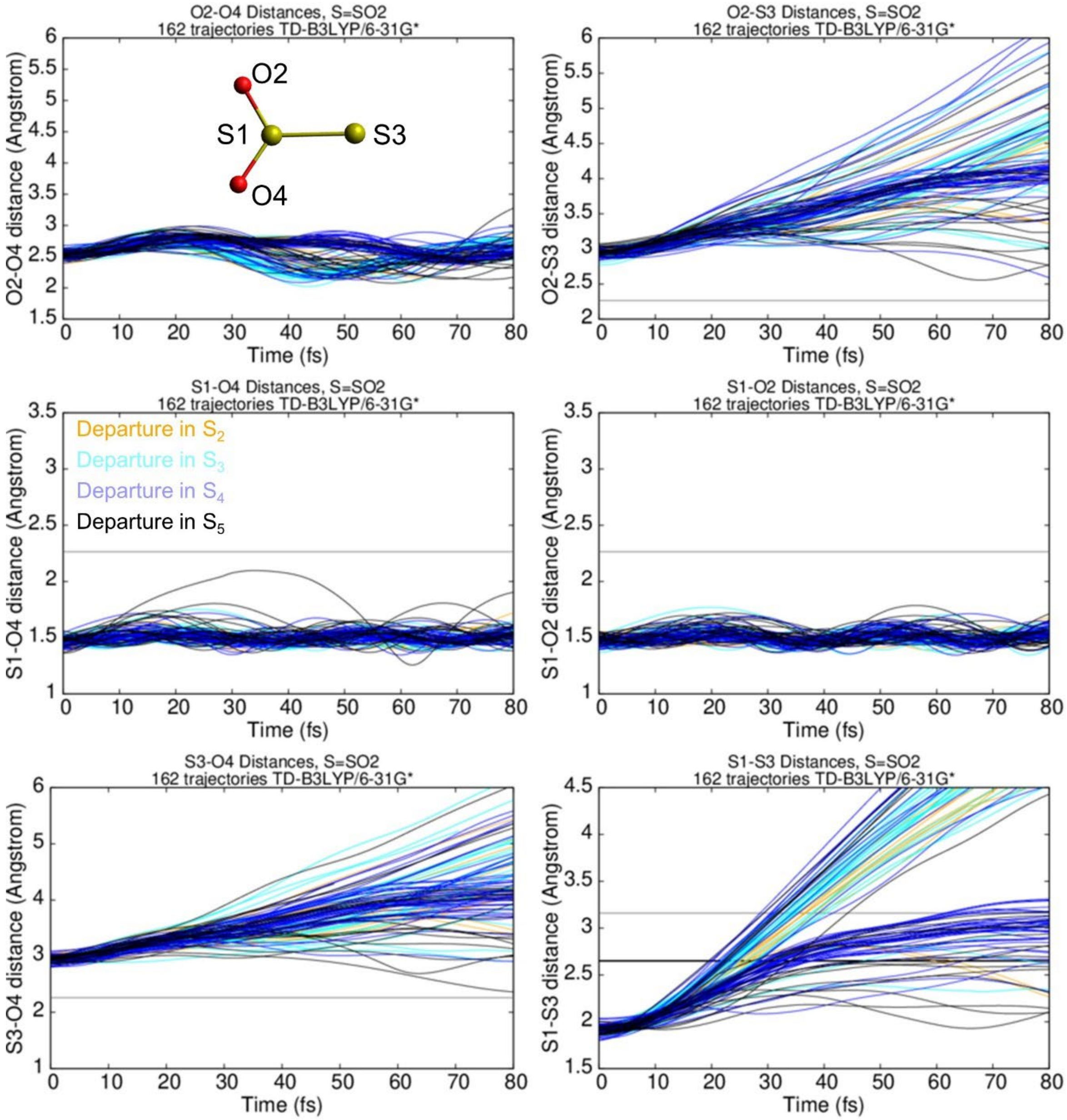

Supplementary Figure 21. Time evolution of the different bond distances for the S=SO_2 system computed with the TD-B3LYP/6-31G* NAMD. The grey bar shows the normal dissociation limit (1.5 times the bond distance at the FC region) and the black bar indicates the TD-DFT dissociation value (verified with MS-CASPT2).

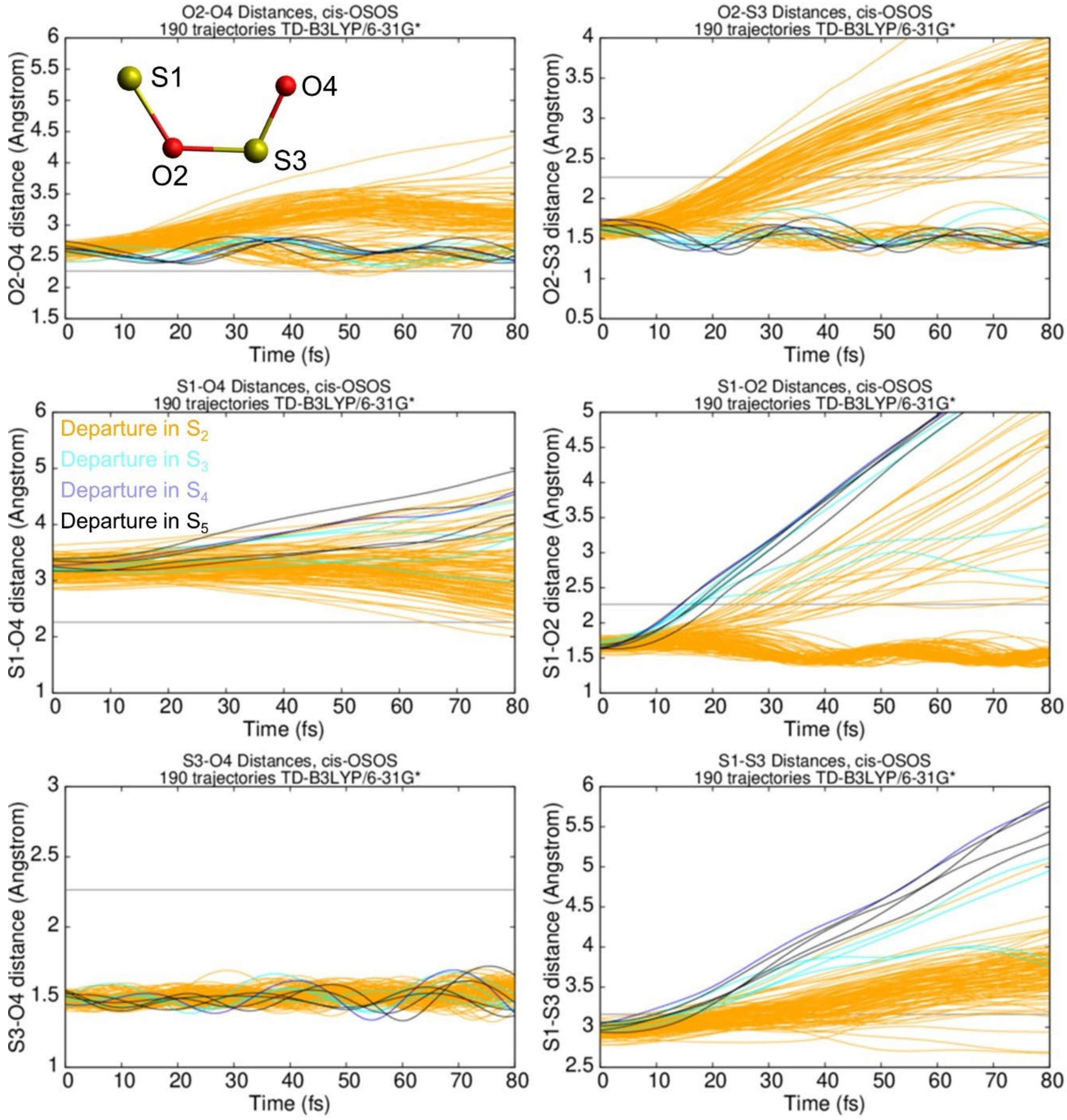

Supplementary Figure 22. Time evolution of the different bond distances for the *cis*-OSOS system computed with the TD-B3LYP/6-31G* NAMD. The grey bar shows the normal dissociation limit (1.5 times the bond distance at the FC region) and the black bar indicates the TD-DFT dissociation value (verified with MS-CASPT2).

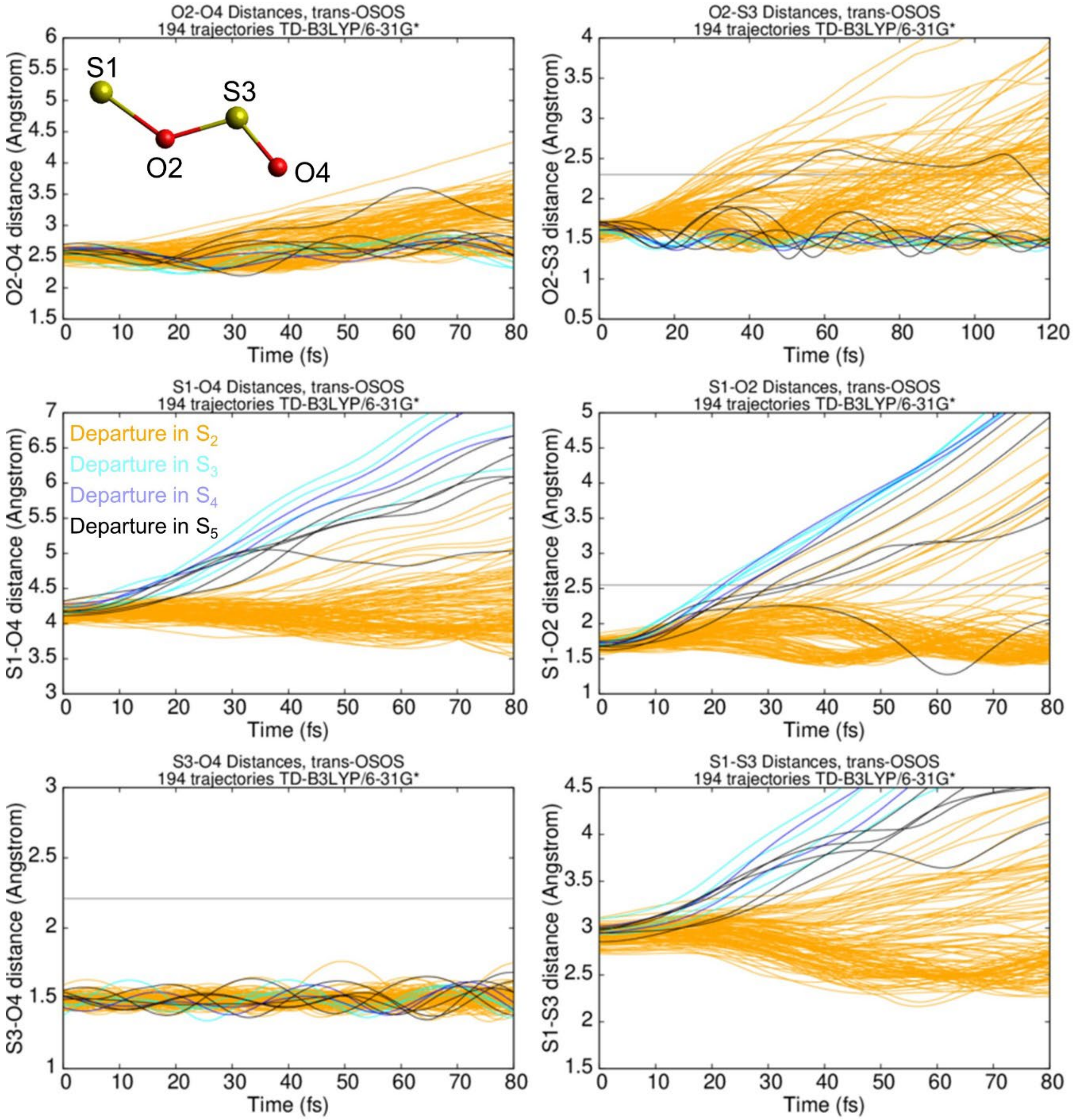

Supplementary Figure 23. Time evolution of the different bond distances for the *trans*-OSOS system computed with the TD-B3LYP/6-31G* NAMD. The grey bar shows the normal dissociation limit (1.5 times the bond distance at the FC region) and the black bar indicates the TD-DFT dissociation value (verified with MS-CASPT2).

Supplementary Table 4. Summary of the number of trajectories per channel and the calculated channel yields for the TD-DFT NAMD runs. A sensibility test of the estimated channel yields as a function of the number of considered NAMD trajectories is listed in the last column. Photodissiociation yields show low dependence on the number of trajectories. The largest deviation between the two analyses is *ca.* 7% for the dominant pathway of the *cyclic*-OS(=O)S photolysis, whereas rarer events remain low when the number of trajectories is enlarged.

**Photoreaction**	**# Trajectories[a]**	**Approximated channel yield (%)[a]**	**# Trajectories[b]**	**Approximated channel yield (%)[b]**	**Error (%)[c]**
***cis*-OSSO**					
cis-OSSO → SO + SO	97/97	100	186/186	100	0
***trans*-OSSO**					
trans-OSSO → SO + SO	71/71	100	136/136	100	0
***cyclic*-OS(=O)S**					
cyclic-OS(=O)S → S + SO_2	40/67	60	89/136	67	7
cyclic-OS(=O)S → SO + SO	23/67	34	43/136	33	1
cyclic-OS(=O)S → S_2O + O	2/67	3	2/136	1	2
cyclic-OS(=O)S → *cis*-OSOS	1/67	1	1/136	1	0
cyclic-OS(=O)S → No reaction	1/67	1	1/136	1	0
**S=SO_2**					
S=SO_2 → SO_2 + S	75/79	95	152/162	94	1
S=SO_2 → No reaction	4/79	5	9/162	6	1
***cis*-OSOS**					
cis-OSOS → SO + SO	68/102	67	127/190	67	0
cis-OSOS → S + SO_2	25/102	25	51/190	27	2
cis-OSOS → *cyclic*-OS(=O)S	1/102	1	3/190	2	1
cis-OSOS → No reaction	8/102	8	9/190	5	3
***trans*-OSOS**					
trans-OSOS → SO + SO	56/98	57	115/194	59	2
trans-OSOS → S + SO_2	22/98	22	48/194	25	3
trans-OSOS → *cyclic*-OS(=O)S	1/98	1	1/194	1	1
trans-OSOS → *cis*-OSOS	0/98	0	2/194	1	1
trans-OSOS → No reaction	19/98	20	28/194	14	6

[a] Only half of the trajectories (preserving the ratio of the initially populated states).
[b] All trajectories.
[c] Difference between the channel yields obtained considering only half of the trajectories and considering the whole set.

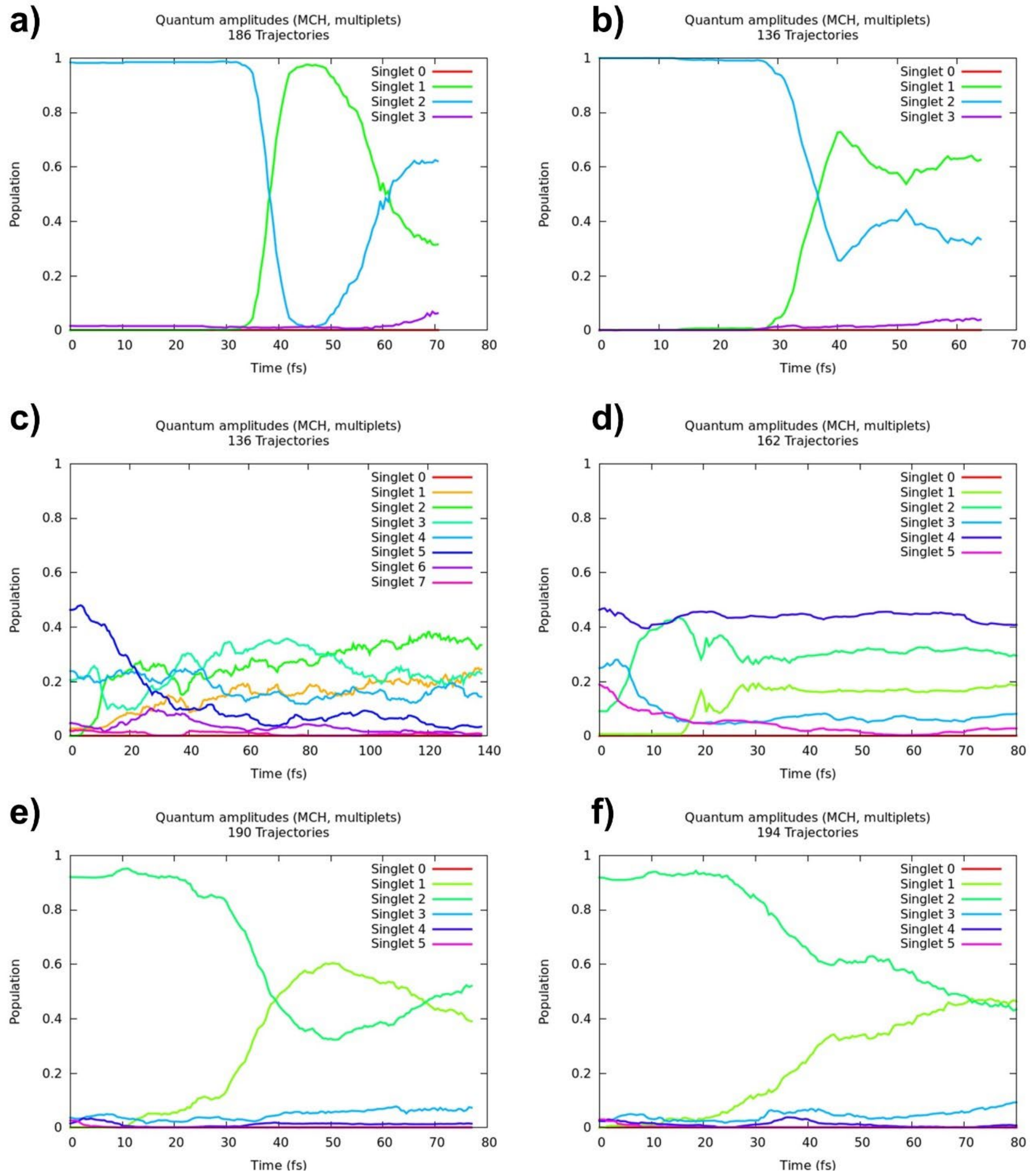

Supplementary Figure 24. Time evolution of the populations for *cis*-OSSO (a), *trans*-OSSO (b), *cyclic*-OS(=O)S (c), S=SO_2 (d), *cis*-OSOS (e), and *trans*-OSOS (f).

4. Supplementary Note 4. Static ground-state CASPT2/MS-CASPT2 reactivity

4.1 Computational details and benchmark analyses

The chemical reactions summarized on Table 1 of the main text were modeled by means of CASPT2 optimizations in combination with the ANO-L-VTZP basis set. The reason for the choice of thermal reactions was to improve old rough estimations of kinetic rates involved in the direct or stepwise generation of S_2 in an alternative manner as photochemical pathway proposed by Pinto et al. [1] (reaction 2 of the main manuscript). We update in Supplementary Table 6 rates related to reactions with energy barriers using conventional transition state theory. Note that for barrierless association reactions forming $(SO)_2$ dimers, such as $^3SO+^3SO$, which are also relevant in the overall modeling, Frandsen et al. [2,25] have recently reported computational data based on single-reference methods and collision theory and variational transition state theory. We used the literature rates for the barrierless reactivity.

Minima and transition states were optimized numerically using the subroutines implemented in OpenMolcas [12]. The true nature of the TSs was verified by analyzing the list of vibrational frequencies (only one negative vibration corresponding to the reaction coordinate) and through intrinsic reaction coordinates (IRCs) calculations to connect the TSs with the corresponding reactants and products. These latter structures were further optimized without constraints to ensure the full relaxation of the structures. In some cases, relaxed scan calculations and/or linear interpolation in internal coordinates (LIIC) were also used to approximate the reaction profiles, using an in-house code for the interpolations and again OpenMolcas to compute wave functions and energies on top of the interpolated structures.

The different active spaces used for each ground-state reaction are listed on Supplementary Table 5. All CASPT2 optimizations were performed computing only one root (state-specific) in the CASSCF procedure. To test the influence of averaging more states in the CASSCF method in the reaction profiles, state-average (SA)-CASSCF computations including 3 roots were also performed on top of the converged structures. The dynamic electron correlation of the 3 states was recovered with the MS-CASPT2 ansatz. With this latter protocol, namely SA(3)-MS-CASPT2, only the first root is displayed in the reaction profiles.

Results show that there is significant state-average effect in some systems, especially in regions close to the TS structures in which the ground and excited states couple more efficiently. A comparative analysis between the CASSCF and the SA(3)-CASSCF wave functions reveal slight differences in the weights of some important configuration state functions, causing the energy differences. In general, the energy barrier heights tend to be smaller at the SA(3)-MS-CASPT2 level of theory as compared to the state-specific CASPT2 method. For reaction (3), in which an accurate experimental rate constant is reported in the literature (~7.5 kcal/mol) [26], the MS-CASPT2 data for reaction (3) gives an energy barrier of 9.0 kcal/mol, showing a clearly better agreement with the experiment. Therefore, SA(3)-MS-CASPT2 energy profiles must be considered to have the highest accuracy. State-specific CASPT2 profiles are used for comparison purposes to display the effects state average and multistate approach.

Supplementary Table 5. CAS used to compute the reaction profiles (optimizations and final energies) with the (MS)-CASPT2/ANO-L-VTZP method and studied spin multiplicities of the chemical transformations. The figures that show the reaction profiles are also listed. The spin of each species shown in the first column corresponds to the lowest-energy configurations.

Thermal reaction	CAS	Studied total multiplicities	Energy profile
$^{3}SO + {}^{1}OSSO \rightarrow {}^{1}OSO + {}^{3}SSO$	16in12	Triplet	Supp. Fig. 25
$^{3}SO + {}^{1}SSO \rightarrow {}^{1}OSO + {}^{3}SS$	12in10	Triplet	Supp. Fig. 27
$^{2}NO + {}^{1}OSSO \rightarrow {}^{2}ONOSSO$	15in12	Doublet	Supp. Fig. 28
$^{2}ONOSSO \rightarrow {}^{2}ONO + {}^{1}SSO$	15in12	Doublet	Supp. Fig. 29
$^{3}O + {}^{1}OSSO \rightarrow {}^{3}OO + {}^{1}SSO$	16in12	Triplet	Supp. Fig. 30
$^{3}O + {}^{1}SSO \rightarrow {}^{3}[OO + SS]$[a]	8in7	Triplet	Supp. Fig. 31
$^{3}S + {}^{1}OSSO \rightarrow {}^{3}SO + {}^{1}SSO$	16in12	Triplet	Supp. Fig. 32
$^{3}S + {}^{1}SSO \rightarrow {}^{3}[SS + SO]$[a]	8in7	Triplet	Supp. Fig. 33
$^{2}H + {}^{1}OSSO \rightarrow {}^{2}HOSSO$	13in11	Doublet	Supp. Fig. 34
$^{2}H + {}^{1}SSO \rightarrow {}^{2}[HO + SS]$[a]	11in9	Doublet	Supp. Fig. 35
$^{3}S + {}^{3}OO \rightarrow {}^{1}SOO$	10in8	Singlet, triplet and quintet	Supp. Fig. 36
$^{1}SOO \rightarrow {}^{3}S + {}^{3}OO$	10in8	Singlet, triplet and quintet	Supp. Fig. 36
$^{1}SOO \rightarrow {}^{3}SO + {}^{3}O$	10in8	Singlet	Supp. Fig. 37
$^{3}SO + {}^{3}O \rightarrow {}^{1}SOO$	10in8	Singlet	Supp. Fig. 37
$^{3}[S + S]$[a] $\rightarrow {}^{3}SS$	16in12	Singlet and triplet	Supp. Fig. 38
$^{3}SS \rightarrow {}^{3}[S + S]$[a]	16in12	Singlet and triplet	Supp. Fig. 38
$^{2}ClS + {}^{3}SO \rightarrow {}^{2}ClSSO$	11in9	Doublet	Supp. Fig. 39
$^{2}ClSSO \rightarrow {}^{2}Cl + {}^{1}SSO$	11in9	Doublet	Supp. Fig. 40
$^{2}ClS + {}^{3}SO \rightarrow {}^{2}ClSOS$	11in9	Doublet	Supp. Fig. 41

[a] At the intermolecular distances of the post-reactive complex, the doublet or triplet state is delocalized over the two reaction products.

4.2 Results

Supplementary Table 6. MS-CASPT2 activation energies ($\Delta E^{\ddagger}$) and energy difference between reactants and products (ΔE) and calculated rates for thermal processes derived from the reactivity between ^{1}OSSO and ^{2}NO / ^{3}O / ^{3}S / ^{2}H, as well as those related to the reaction between ^{2}ClS and ^{3}SO. Energetic profiles of the association of ^{3}S and $^3O_2$ / S are also shown. The value estimated from experimental measurements for the reaction ^{3}SO + ^{1}OSSO → $^1SO_2$ + ^3S_2O is shown within parenthesis. Unimolecular and bimolecular rate constants have been computed by means of transition state theory at T=298 K. Pseudo-first order rates were calculated using the concentration values of the reactants presented in Supplementary Table 8 from Zhang et al. [27] except for ^{3}SO and ^{1}OSSO, for which the concentrations computed in this work have been used.

Thermal reaction	$\Delta E^{\ddagger}$ (kcal/mol)	ΔE (kcal/mol)	Unimolecular rate (s^{-1})	Bimolecular rate (molecule^{-1} cm^3 s^{-1})	Pseudo-first order rate (s^{-1})
^{3}SO + ^{1}OSSO → ^{1}OSO + ^{3}SSO	9.0 (~7.5)^a	-1.9		2.6×10^{-15} (3.3×10^{-14})^a	6.2×10^{-06} 7.9×10^{-05}
^{3}SO + ^{1}SSO → ^{3}OSOSS	0.1 – 2.0^b	-15.7		8.7×10^{-09} – 3.5×10^{-10}	20.9 – 0.8
^{3}OSOSS → ^{1}OSO + ^{3}SS	2.1	-26.0	$1.8\times10^{+11}$		
^{2}NO + ^{1}OSSO → ^{2}ONOSSO	16.3	13.4		1.1×10^{-20}	2.1×10^{-10}
^{2}ONOSSO → ^{2}ONO + ^{1}SSO	~27.1	~24.4	8.3×10^{-08}		
^{3}O + ^{1}OSSO → ^{3}OO + ^{1}SSO	16.1	-34.3		1.6×10^{-20}	1.1×10^{-12}
^{3}O + ^{1}SSO → ^{3}[OO + SS]	18.9	-26.2		1.4×10^{-22}	1.0×10^{-14}
^{3}S + ^{1}OSSO → ^{3}SO + ^{1}SSO	10.7	-35.1		1.5×10^{-16}	7.3×10^{-09}
^{3}S + ^{1}SSO → ^{3}[SS + SO]	10.9	-28.0		1.0×10^{-16}	3.7×10^{-10}
^{2}H + ^{1}OSSO → ^{2}HOSSO	5.5	-66.0		9.5×10^{-13}	4.6×10^{-05}
^{2}H + ^{1}SSO → ^{2}[HO + SS]	6.1	-59.5		3.5×10^{-13}	1.3×10^{-06}
^{3}S + ^{3}OO → ^{1}SOO	0.0	-13.6		2.3×10^{-12c}	7.8×10^{-03}
^{1}SOO → ^{3}S + ^{3}OO	13.6	13.6	$6.6\times10^{+02}$		
^{1}SOO → ^{3}SO + ^{3}O	13.5	9.4	$7.8\times10^{+02}$		
^{3}SO + ^{3}O → ^{1}SOO	4.1	-9.4		1.0×10^{-11}	2.4×10^{-02}
^{3}[S + S] → ^{3}SS	0.0	-97.5		1.0×10^{-13d}	2.4×10^{-07}
^{3}SS → ^{3}[S + S]	97.5	97.5	1.9×10^{-59}		
^{2}ClS + ^{3}SO → ^{2}ClSSO	0.0	-38.2		1.0×10^{-11c}	2.4×10^{-02}
^{2}ClSSO → ^{2}Cl + ^{1}SSO	25.6	25.6	1.0×10^{-06}		
^{1}*cis*-OSSO + ^{1}*cis*-OSSO → ^{1}cyclic-S_4O_4	9.0^e	-4.1^e		2.6×10^{-15}	1.3×10^{-7}
^{1}cyclic-S_4O_4 → ^{1}*cis*-S_3O_2 + $^1SO_2$	12.0^e	-6.3^e	$9.8\times10^{+03}$		

^{a}Bimolecular rate reported by Herron et al. [26].

^{b}Range of $\Delta E^{\ddagger}$ values obtained from state-specific CASSCF/CASPT2 and state-average multistate SA(3)-CASSCF/MS-CASPT2 approaches (see section 4.1).

^{c}Bimolecular rate constant from Zhang et al. [27] for the global reaction S + O_2 → SO + O and ClS + SO → Cl + S_2O.

^{d}High-pressure rate constant from Du et al.[28]

^{e}Values obtained at the (U)CCSD(T)/aug-cc-pVQZ level of theory (Supplementary Table 9).

Supplementary Table 7. Photolysis rates from *cis-/trans*-OSSO to produce SO from Frandsen et al.,[2] with Venusian altitude set to 64 km and latitude 0º and updated value considering the yields of photogenerated SO computed in this work.

	**Photolysis rate reported (s^{-1})[a]**	**Yield (%)[b]**	**Photolysis rate updated (s^{-1})[b]**
^{1}*cis*-OSSO + hν → ^{3}SO + ^{3}SO	0.20	95%	0.19
^{1}*trans*-OSSO + hν → ^{3}SO + ^{3}SO	0.62	90%	0.56

[a]Frandsen et al. [2]

[b]This work.

Supplementary Table 8. Number density (reported and computed here) in the Venusian atmosphere at 64 km altitude of the species involved in the bimolecular reactions studied in this work. We considered an atmospheric number density of 3.4E+18 molecules cm^{-3} at this altitude. The reported values presented here have been obtained by digitizing the figures from Zhang et al. [27] and Pinto et al. [1]. To compute the pseudo-first order rates, the concentration of the most abundant species in each reaction was used.

**Species**	**Number density (Zhang et al.) (molecule cm^{-3})**	**Number density (this work) (molecule cm^{-3})**	**Number density (Pinto et al.) (molecule cm^{-3})**
^{2}H	4.42E+04		
^{3}SO	1.70E+09	2.40E+09	2.30E+09
^{3}S	2.38E+06		
^{3}O	7.14E+07		
^{2}NO	1.87E+10		
$^{1}(SO)_2$	3.74E+01	4.86E+07	5.10E+08
$^{1}S_2O$	8.16E+07	3.65E+06	1.40E+09
^{2}ClS	4.08E+07		
$^{3}O_2$	3.40E+09		

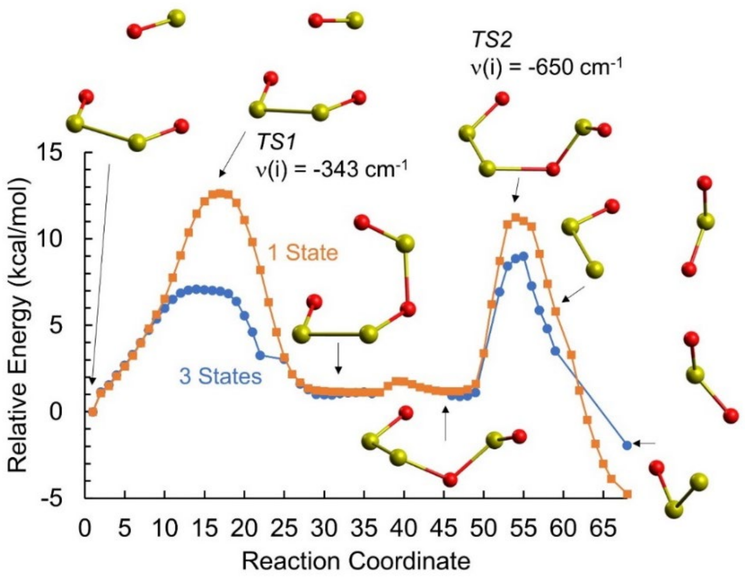

Supplementary Figure 25. ^3SO + ^1OSSO → $^1SO_2$ + ^3S_2O reaction profile. The dark orange line represents the CASPT2 energies computing 1 state in the CASSCF procedure. The blue line represents the MS-CASPT2 ground-state energy demanding 3 roots in the SA-CASSCF method. Geometries have been optimized only at the CASPT2 level of theory considering only 1 root (dark orange profile), whereas the MS-CASPT2 energies have been computed on top of the optimized geometries with the previous method. Reaction coordinate points 1-36 and 46-59 correspond to IRC determinations, whereas points 37-45 and 61-68 have been obtained through linear interpolations in internal coordinates.

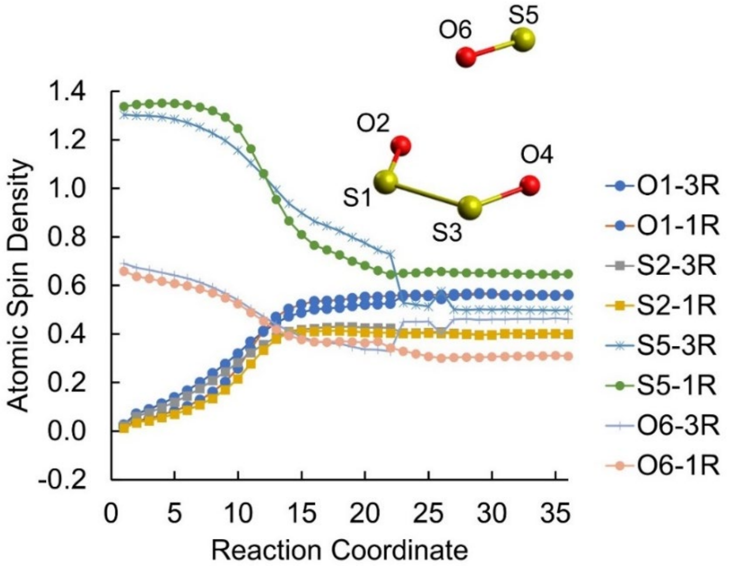

Supplementary Figure 26. CASSCF (1R) and SA-CASSCF (3R) Mulliken spin densities computing 1 and 3 states, respectively, along the first step of the ^3SO + ^1OSSO → $^1SO_2$ + ^3S_2O reaction. Differences in spin densities are small but have a rather important impact in states' energies, as shown in the previous Figure.

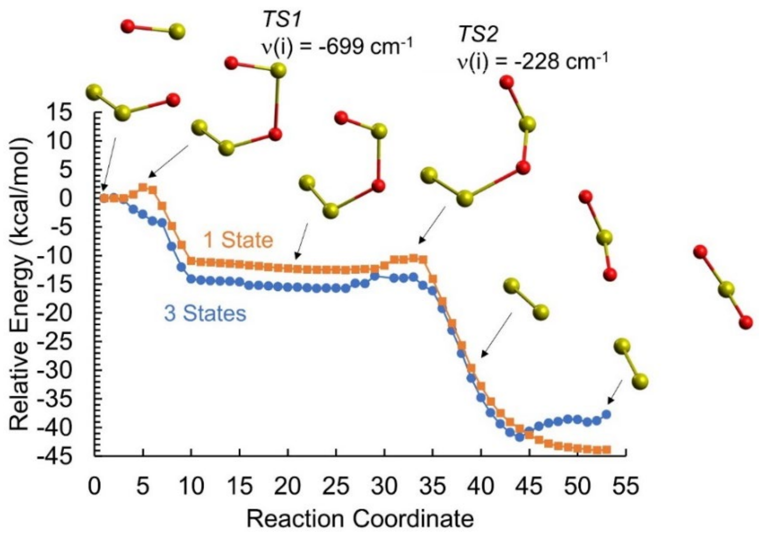

Supplementary Figure 27. ^3SO + ^1SSO → $^1SO_2$ + $^3S_2$ reaction profile. The dark orange line represents the CASPT2 energies computing 1 state in the CASSCF procedure. The blue line represents the MS-CASPT2 ground-state energy demanding 3 roots in the SA-CASSCF method. Geometries have been optimized only at the CASPT2 level of theory considering only 1 root (dark orange profile), whereas the MS-CASPT2 energies have been computed on top of the optimized geometries with the previous method. All reaction coordinate points correspond to IRC determinations.

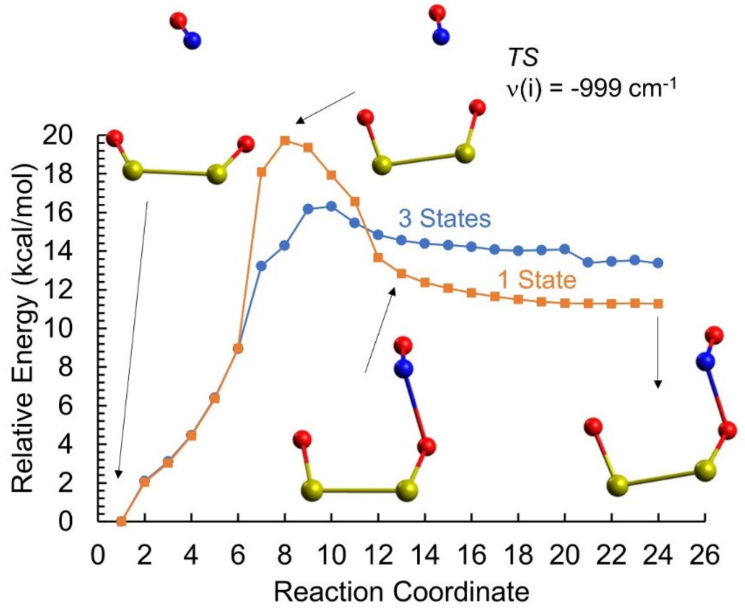

Supplementary Figure 28. ^2NO + ^1OSSO → ^2ONOSSO reaction profile. The dark orange line represents the CASPT2 energies computing 1 state in the CASSCF procedure. The blue line represents the MS-CASPT2 ground-state energy demanding 3 roots in the SA-CASSCF method. Geometries have been optimized only at the CASPT2 level of theory considering only 1 root (dark orange profile), whereas the MS-CASPT2 energies have been computed on top of the optimized geometries with the previous method. All reaction coordinate points correspond to IRC determinations. N atom represented in blue.

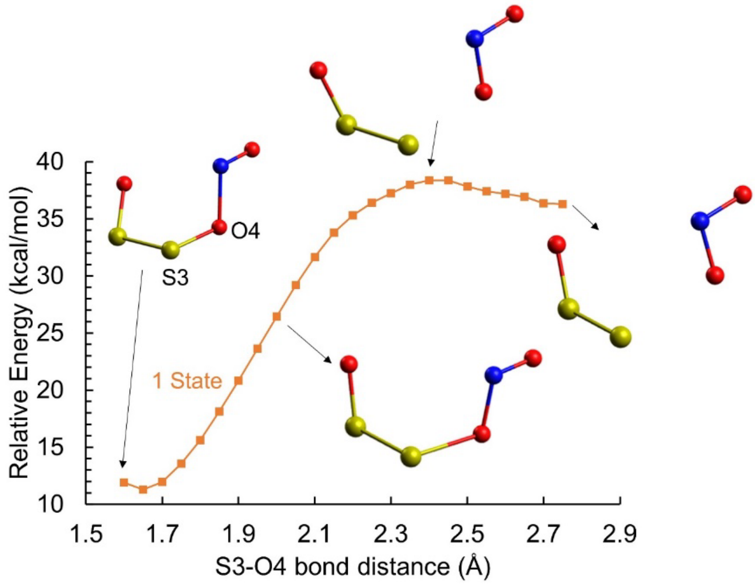

Supplementary Figure 29. $^{2}ONOSSO \rightarrow {}^{2}NO_2 + {}^{1}SSO$ reaction profile obtained through a relaxed scan of the S3-O4 bond with the CASPT2 method computing 1 state in the CASSCF procedure.

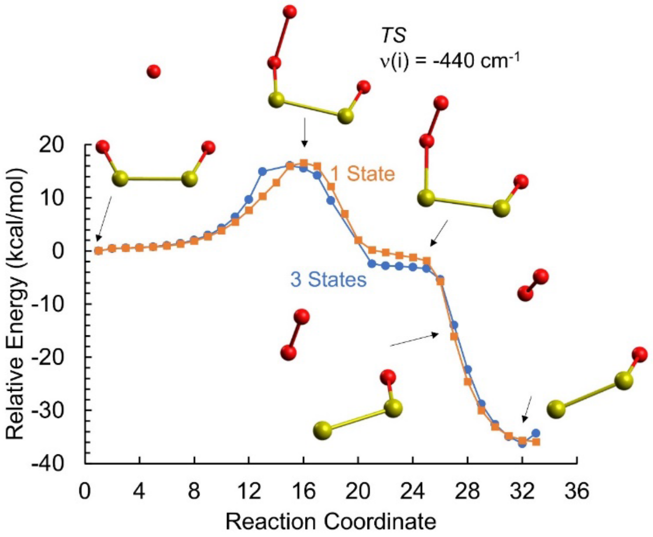

Supplementary Figure 30. $^{3}O + {}^{1}OSSO \rightarrow {}^{3}O_2 + {}^{1}S_2O$ reaction profile. The dark orange line represents the CASPT2 energies computing 1 state in the CASSCF procedure. The blue line represents the MS-CASPT2 ground-state energy demanding 3 roots in the SA-CASSCF method. Geometries have been optimized only at the CASPT2 level of theory considering only 1 root (dark orange profile), whereas the MS-CASPT2 energies have been computed on top of the optimized geometries with the previous method. Reaction coordinate points 1-25 correspond to IRC determinations, whereas points 26-33 have been obtained through linear interpolations in internal coordinates.

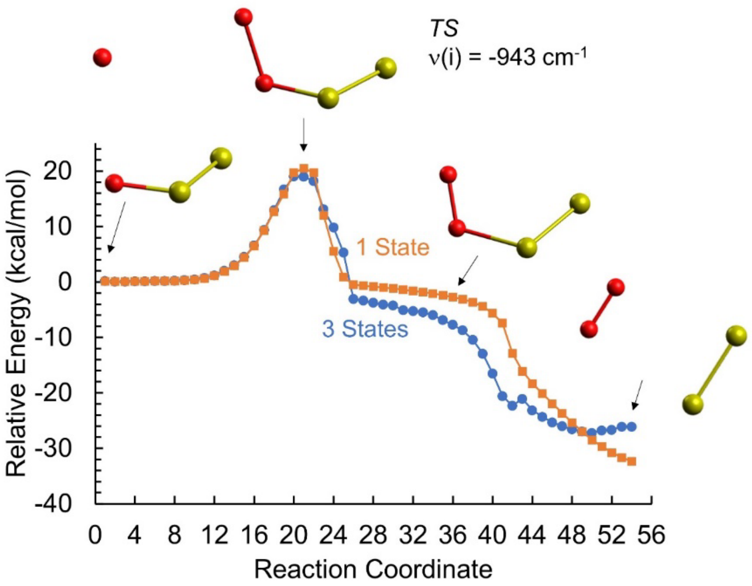

Supplementary Figure 31. $^3O + {}^1SSO \rightarrow {}^3[O_2 + S_2]$ reaction profile. The dark orange line represents the CASPT2 energies computing 1 state in the CASSCF procedure. The blue line represents the MS-CASPT2 ground-state energy demanding 3 roots in the SA-CASSCF method. Geometries have been optimized only at the CASPT2 level of theory considering only 1 root (dark orange profile), whereas the MS-CASPT2 energies have been computed on top of the optimized geometries with the previous method. All reaction coordinate points correspond to IRC determinations.

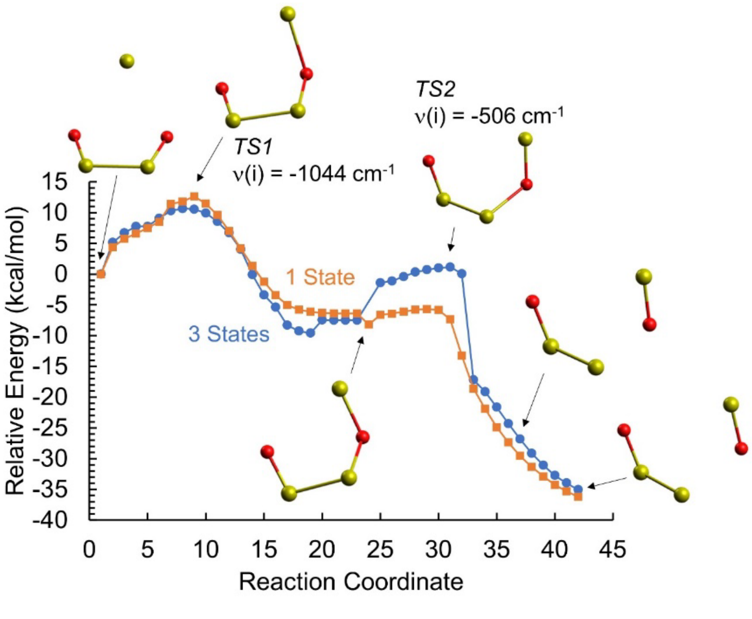

Supplementary Figure 32. $^3S + {}^1OSSO \rightarrow {}^3SO + {}^1S_2O$ reaction profile. The dark orange line represents the CASPT2 energies computing 1 state in the CASSCF procedure. The blue line represents the MS-CASPT2 ground-state energy demanding 3 roots in the SA-CASSCF method. Geometries have been optimized only at the CASPT2 level of theory considering only 1 root (dark orange profile), whereas the MS-CASPT2 energies have been computed on top of the optimized geometries with the previous method. All reaction coordinate points correspond to IRC determinations.

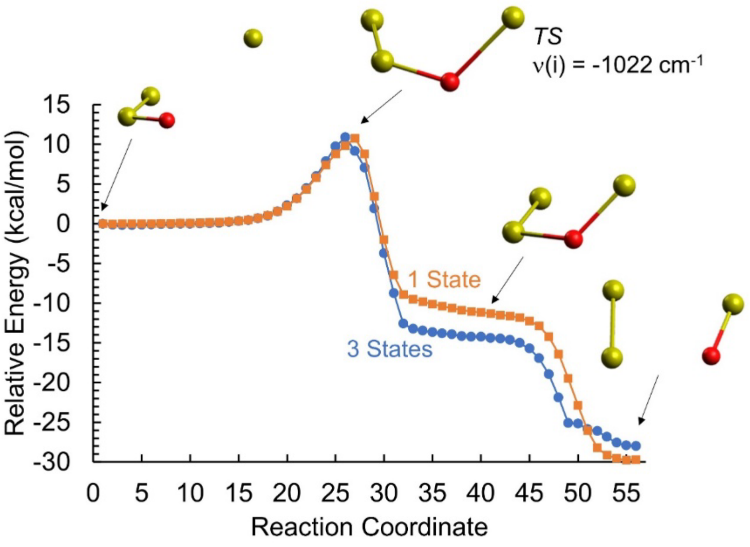

Supplementary Figure 33. ^{3}S + ^{1}SSO → ^{3}[SO + S_2] reaction profile. The dark orange line represents the CASPT2 energies computing 1 state in the CASSCF procedure. The blue line represents the MS-CASPT2 ground-state energy demanding 3 roots in the SA-CASSCF method. Geometries have been optimized only at the CASPT2 level of theory considering only 1 root (dark orange profile), whereas the MS-CASPT2 energies have been computed on top of the optimized geometries with the previous method. All reaction coordinate points correspond to IRC determinations.

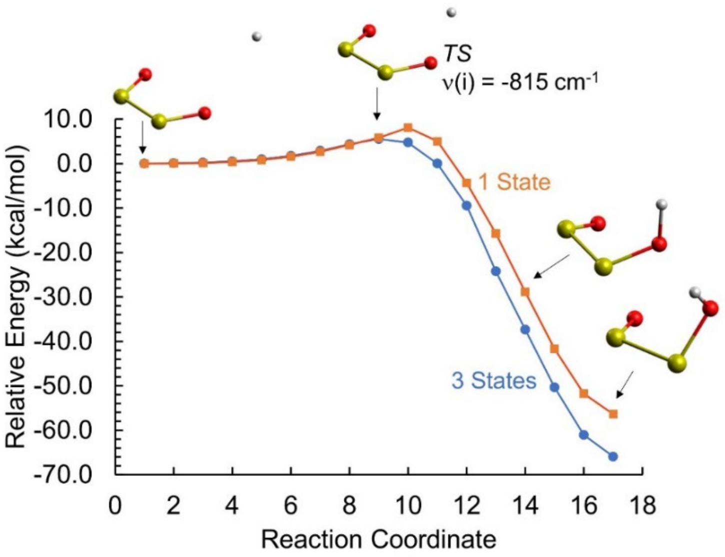

Supplementary Figure 34. ^{2}H + ^{1}OSSO → ^{2}HOSSO reaction profile. The dark orange line represents the CASPT2 energies computing 1 state in the CASSCF procedure. The blue line represents the MS-CASPT2 ground-state energy demanding 3 roots in the SA-CASSCF method. Geometries have been optimized only at the CASPT2 level of theory considering 1 root (dark orange profile), whereas the MS-CASPT2 energies have been computed on top of the optimized geometries with the previous method. All reaction coordinate points correspond to interpolations and therefore the TS is only approximated. The frequencies analysis corroborates its TS character even though it was not possible to achieve geometry convergence with the OpenMolcas optimizer. H atom represented in white.

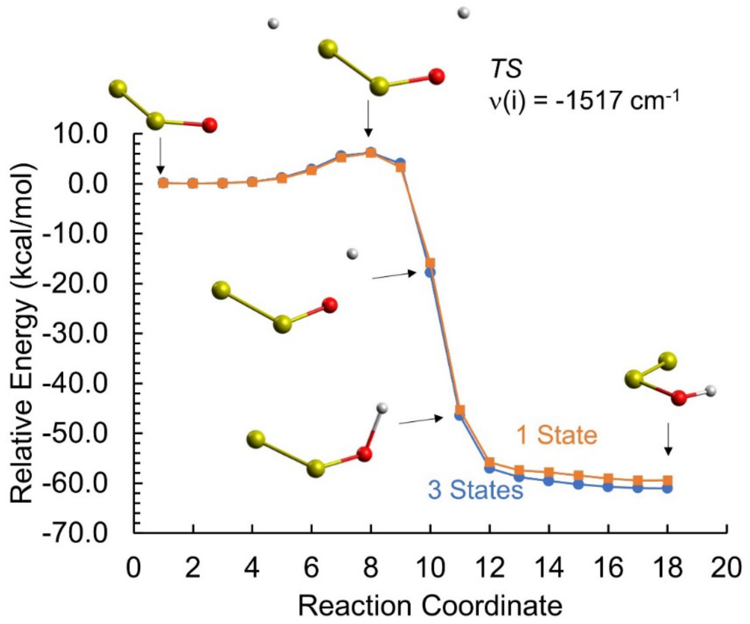

Supplementary Figure 35. ^2H + ^1SSO → ^2HSSO reaction profile. The dark orange line represents the CASPT2 energies computing 1 state in the CASSCF procedure. The blue line represents the MS-CASPT2 ground-state energy demanding 3 roots in the SA-CASSCF method. Geometries have been optimized only at the CASPT2 level of theory considering only 1 root (dark orange profile), whereas the MS-CASPT2 energies have been computed on top of the optimized geometries with the previous method. All reaction coordinate points correspond to IRC determinations.

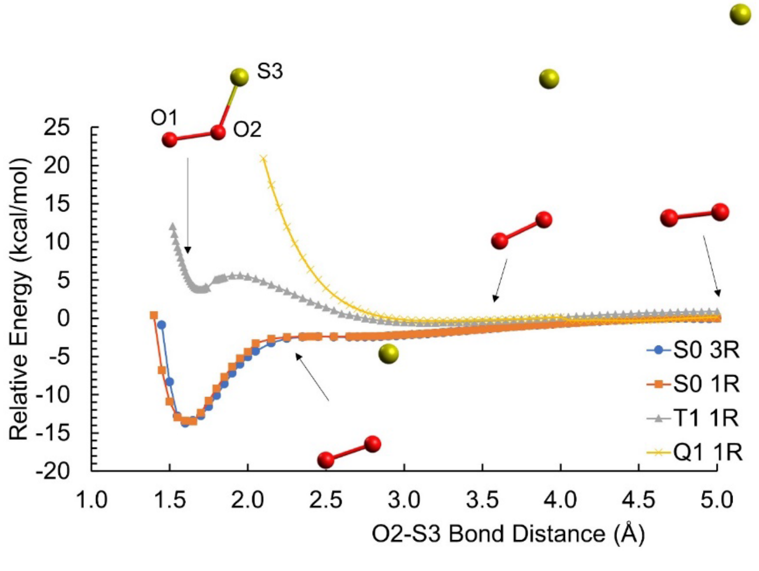

Supplementary Figure 36. ^3S + ^3OO → ^1SOO reaction profile obtained through a relaxed scan of the O2-S3 bond with the CASPT2 method computing 1 state (1R) in the CASSCF procedure for each multiplicity. MS-CASPT2 energies have been computed on top of the S_0 optimized geometries averaging 3 states in the SA-CASSCF method.

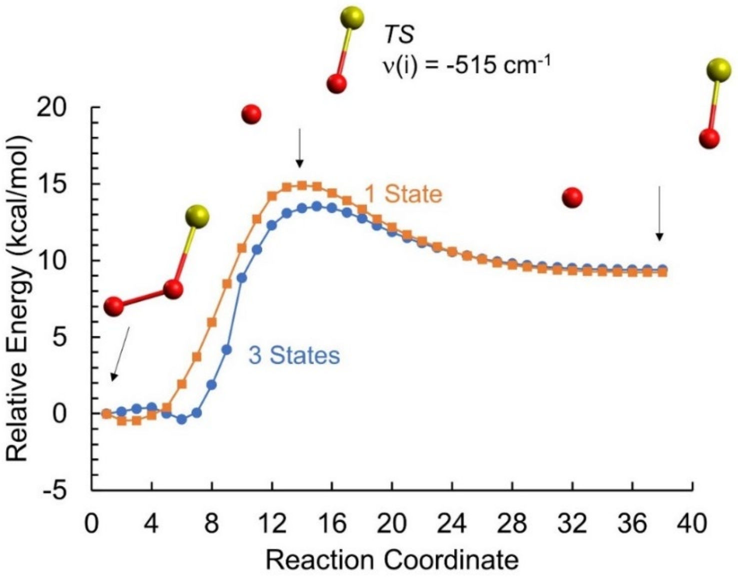

Supplementary Figure 37. $^1SOO \rightarrow {}^3SO + {}^3O$ reaction profile. The dark orange line represents the CASPT2 energies computing 1 state in the CASSCF procedure. The blue line represents the MS-CASPT2 ground-state energy demanding 3 roots in the SA-CASSCF method. Geometries have been optimized only at the CASPT2 level of theory considering only 1 root (dark orange profile), whereas the MS-CASPT2 energies have been computed on top of the optimized geometries with the previous method. All reaction coordinate points correspond to IRC determinations.

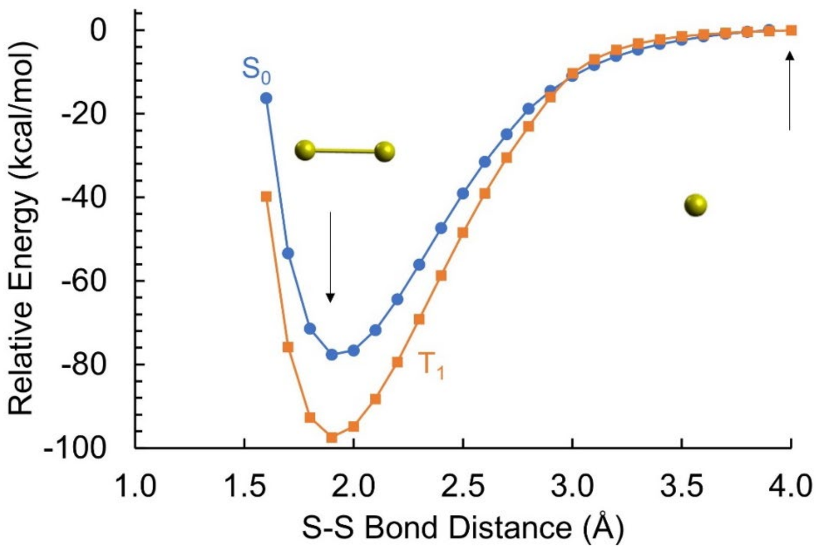

Supplementary Figure 38. $^3[S + S] \rightarrow {}^3S_2$ reaction profile obtained through a relaxed scan of the S-S bond with the CASPT2 method computing 1 state (1R) in the CASSCF procedure for each multiplicity.

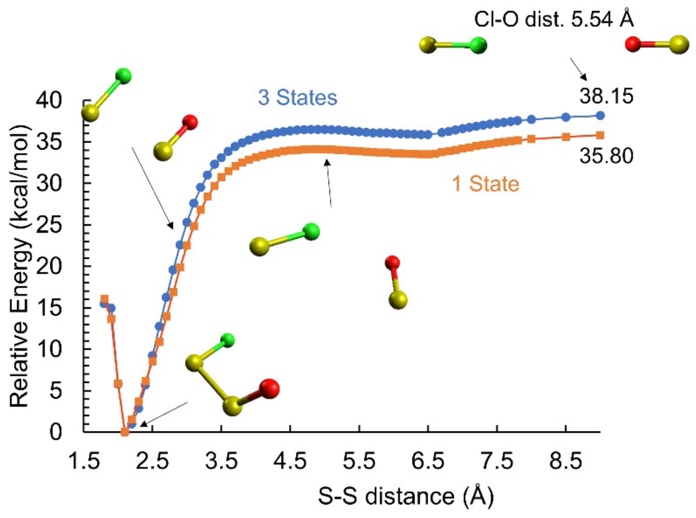

Supplementary Figure 39. $^{2}ClS + ^{3}SO \rightarrow ^{2}ClSSO$ reaction profile. The dark orange line represents the CASPT2 energies computing 1 state in the CASSCF procedure. The blue line represents the MS-CASPT2 ground-state energy demanding 3 roots in the SA-CASSCF method. Geometries have been optimized only at the CASPT2 level of theory considering only 1 root (dark orange profile), whereas the MS-CASPT2 energies have been computed on top of the optimized geometries with the previous method. The x axis corresponds to the relaxed scan of the S-S bond distances. Cl atom represented in green.

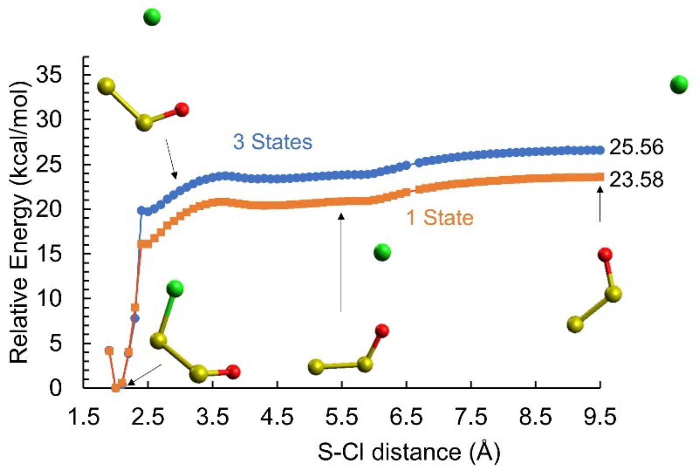

Supplementary Figure 40. $^{2}ClSSO \rightarrow ^{2}Cl + ^{1}SSO$ reaction profile. The dark orange line represents the CASPT2 energies computing 1 state in the CASSCF procedure. The blue line represents the MS-CASPT2 ground-state energy demanding 3 roots in the SA-CASSCF method. Geometries have been optimized only at the CASPT2 level of theory considering only 1 root (dark orange profile), whereas the MS-CASPT2 energies have been computed on top of the optimized geometries with the previous method. The x axis corresponds to the relaxed scan of the Cl-S bond distances.

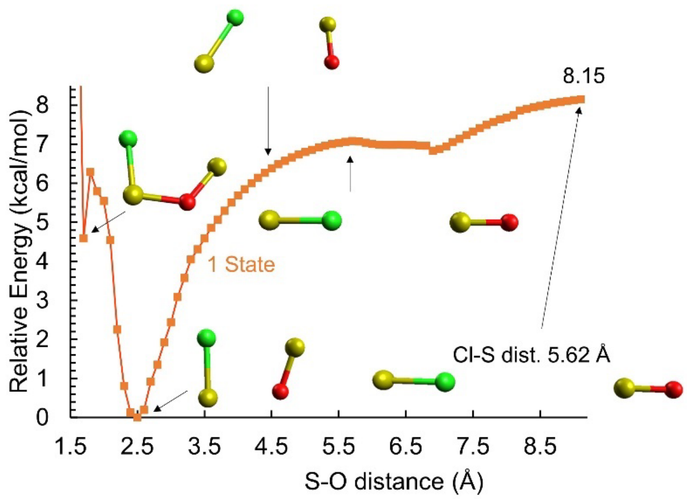

Supplementary Figure 41. ^2ClS + ^3OS → ^2ClSOS reaction profile. The dark orange line represents the CASPT2 energies computing 1 state in the CASSCF procedure. Geometries have been optimized only at the CASPT2 level of theory considering only 1 root (dark orange profile), whereas the MS-CASPT2 energies have been computed on top of the optimized geometries with the previous method. The x axis corresponds to the relaxed scan of the S-O bond distances.

5. Supplementary Note 5. Excited-state MS-CASPT2 energy profiles

The energetic profiles for some particularly interesting excited-state dissociations have been computed with the MS-CASPT2/ANO-S-VDZP method and the OpenMolcas software.[12]

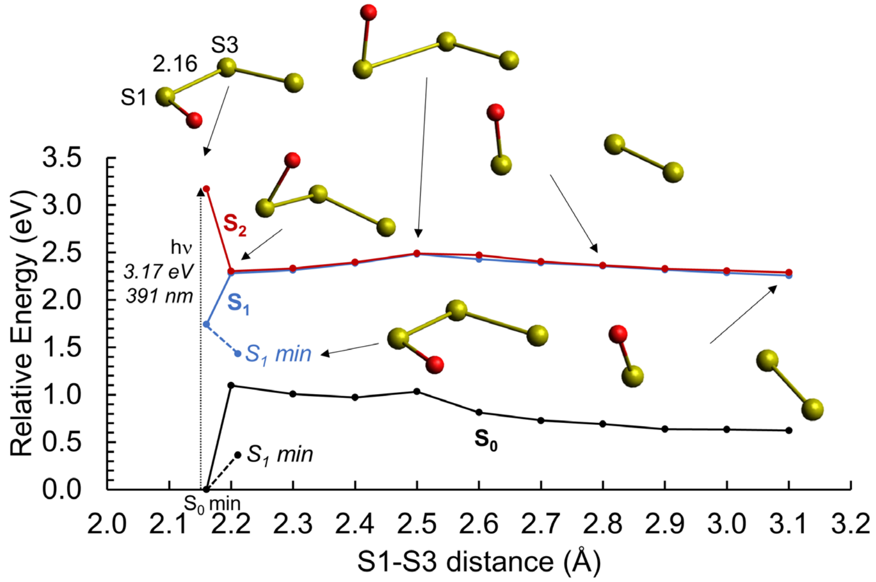

Supplementary Figure 42. MS-CASPT2 relaxed scan of ^{1}*cis*-SSSO, relaxing the $\mathbf{S_2}$ state. The absorption energy to populate the $\mathbf{S_2}$ state at the $\mathbf{S_0}$ min region (S1-S3 distance of 2.16 Å) is shown through the dotted arrow. The energy of the $\mathbf{S_0}$ and $\mathbf{S_1}$ states at the $\mathbf{S_1}$ min geometry (S1-S3 distance of 2.21 Å) is also shown. Four roots have been computed in the SA-CASSCF method.

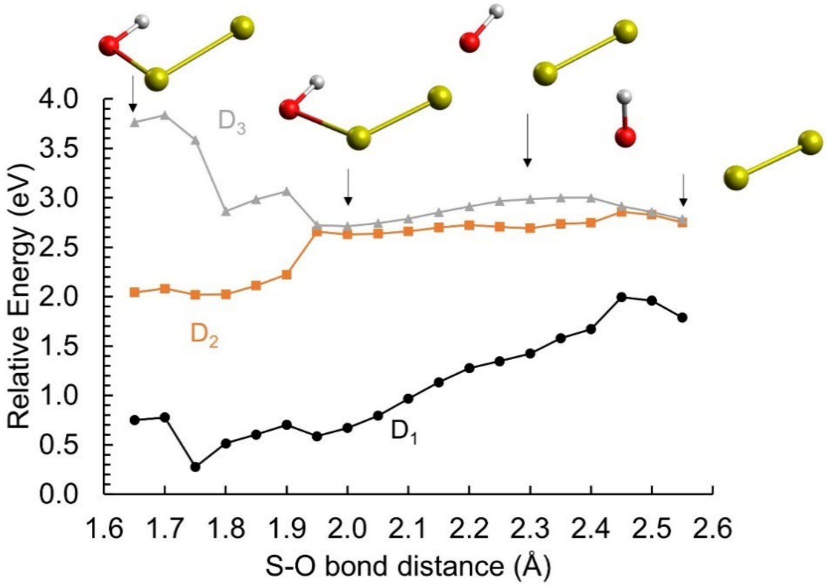

Supplementary Figure 43. ^{2}HOSS → ^{2}[HO + S_2] excited-state reaction along the S-O bond stretch relaxing the D_2 state at the MS-CASPT2 level of theory. Four roots have been computed in the SA-CASSCF method.

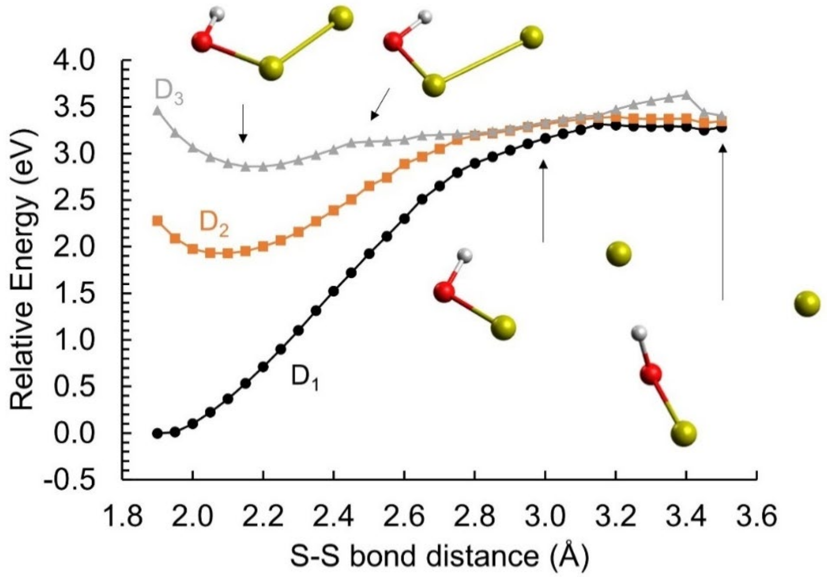

Supplementary Figure 44. ^{2}HOSS → ^{2}HOS + ^{3}S excited-state reaction along the S-S bond stretch relaxing the D_2 state at the MS-CASPT2 level of theory. Four roots have been computed in the SA-CASSCF method.

6. Supplementary Note 6. Ground-state DFT reactivity

6.1 Computational details

DFT optimizations were carried out using the B3LYP/6-311G(d,p) level of theory as implemented in the Gaussian 16 program.[29] The restricted scheme was employed for systems with singlet multiplicity, whereas the unrestricted DFT ansatz was employed for triplet states. The nature of each stationary point was verified by examining the list of frequencies (all positive for minima, one negative and the rest positive for transition states). Intrinsic reaction coordinate (IRC) computations were systematically computed to ensure the connectivity between reactants, transition states, and products. Unconstrained optimizations were performed to the final structures of the IRC calculations in order to ensure the complete relaxation of reactants and products.

6.2 Results

In Supplementary Figure 45, (^{1}*cis*-OSSO + ^{1}*cis*-OSSO → ^{1}*cis*-S_3O_2 + $^{1}SO_2$), the first transition state (**TS1**) implies the formation of the S2-O6 bond and the rotation of the O8 atom around the O6-S-S-O8 dihedral angle. The energetic barrier from the reactants ^{1}*cis*-OSSO + ^{1}*cis*-OSSO (**R1**, right-hand side of the plot) is of 0.22 eV (5.1 kcal/mol). Molecular motions toward the intermediate ^{1}*cyclic*-S_4O_4 **I1** forms the S-S bond barrierless, releasing 0.71 eV (16.4 kcal/mol).

The formation of **TS2** implies the breaking of the S2-S3 and O6-S bonds, giving a structure with a relative energy of -0.03 eV. The energy barrier from **I1** to **TS2** is thus of 0.46 eV (10.6 kcal/mol). The release of the $^{1}SO_2$ molecule and the stabilization of the ^{1}*cis*-S_3O_2 is barrierless, releasing 0.56 eV (12.9 kcal/mol) and yielding the van der Waals complex **P1** ($^{1}SO_2$ + ^{1}*cis*-S_3O_2) with a relative energy of -0.59 eV (13.6 kcal/mol).

The left-hand side of Supplementary Figure 45 shows the reaction ^{1}*cis*-S_3O_2 + $^{1}SO_2$ → $^{1}S_2$ + 2 $^{1}SO_2$. The ^{1}*cis*-S_3O_2 decomposition to yield the products **P2** has a global energy barrier of 0.81 eV (18.7 kcal/mol) mainly associated to the formation of the linear species S-S-O-S-O (**I2**). The chemical channel goes through two planar areas, ***TS3*** and ***TS4***. The bond breaking of the O8-S7 that releases the second SO_2 molecule and S_2 is barrierless. Further reaction profiles with SO are shown in Supplementary Figures 46-47.

On the other hand, Supplementary Figures 48-50 compute the reaction profiles for an alternative reaction of ^{1}*cis*-S_3O_2 with ^{3}SO.

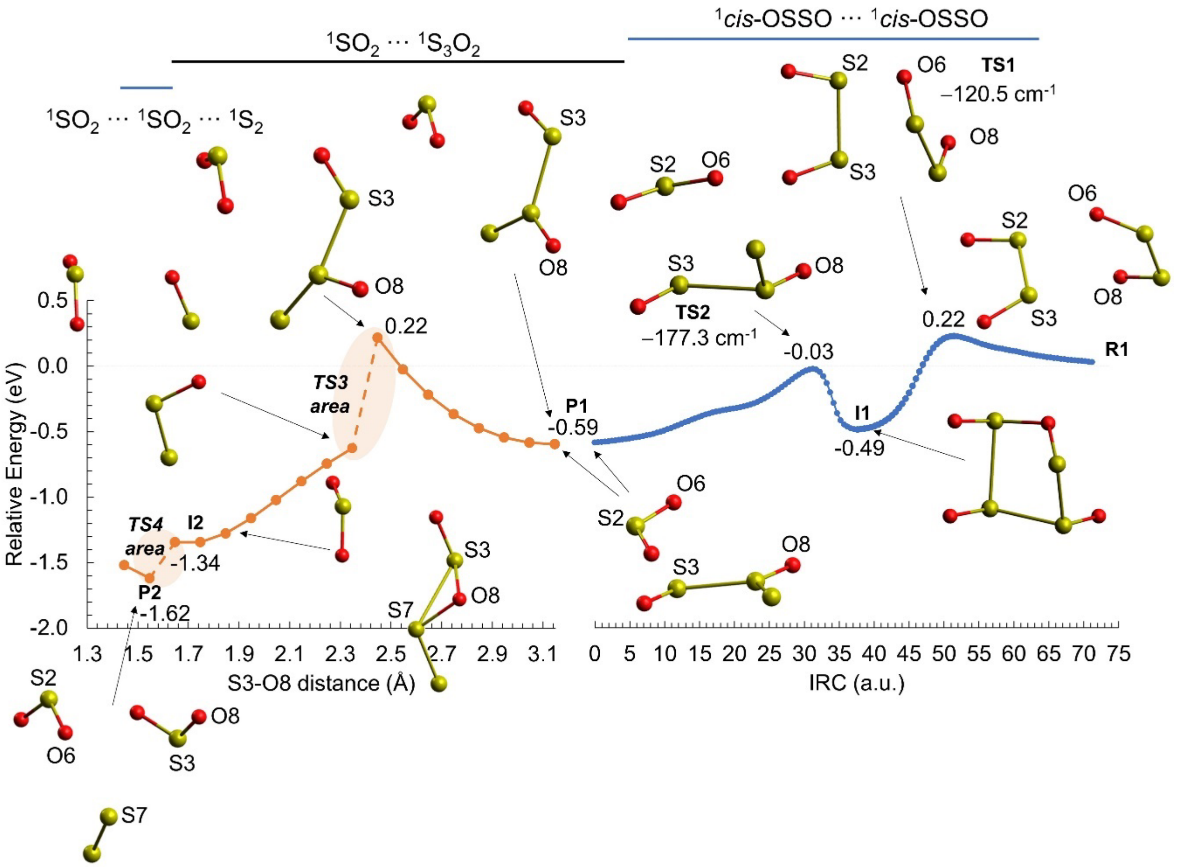

Supplementary Figure 45. Reaction of two ^{1}*cis*-OSSO molecules to yield ^{1}*cis*-S_3O_2 and $^{1}SO_2$ (right), followed by the ^{1}*cis*-S_3O_2 decomposition to yield $^{1}SO_2$ and $^{1}S_2$ (left). Reactants are at the right-hand and products at the left-hand of the plot in coherence to the progression of the S3-O8 bond shortening. The profiles have been obtained by means of TS optimizations followed by IRC calculations to connect reactants, TSs, and products (right) and through relaxed scans of the S3-O3 bond distance (left) shrinkages at the B3LYP/ 6-311G(d,p) level of theory. Dashed lines indicate non-connected points due to molecular reorganizations involving other molecular coordinates that have been studied separately (see below). ***TS3*** and ***TS4 areas*** refer to transition state areas (shadowed in the plot) identified along the PES by visual inspection, the highest-energy structures are not the result of TS optimizations.

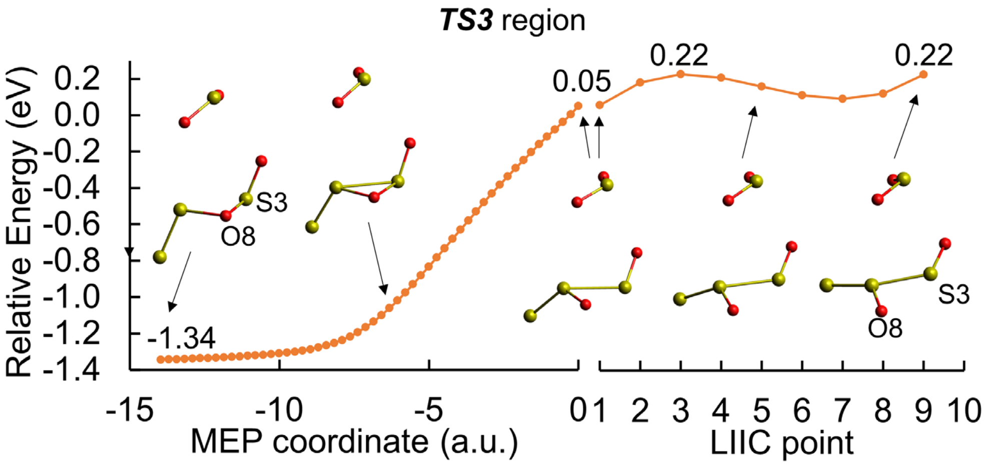

Supplementary Figure 46. Exploration of the ***TS3*** region through a combination of LIIC (right) and MEP (left) techniques. The former maps the pyramidalization of one sulfur atom while the S3-O8 bond distance varies going from 2.397 Å at LIIC point 9 to 2.297 Å at LIIC point 1, although at point 5 the distance is stretched up to 2.489 Å. The MEP shows the barrierless path towards the formation of the linear species S-S-O-S-O (**I2**). The upper bound for the total energy barrier of this process is thus 0.22 – (–0.59) = 0.81 eV (18.9 kcal/mol).

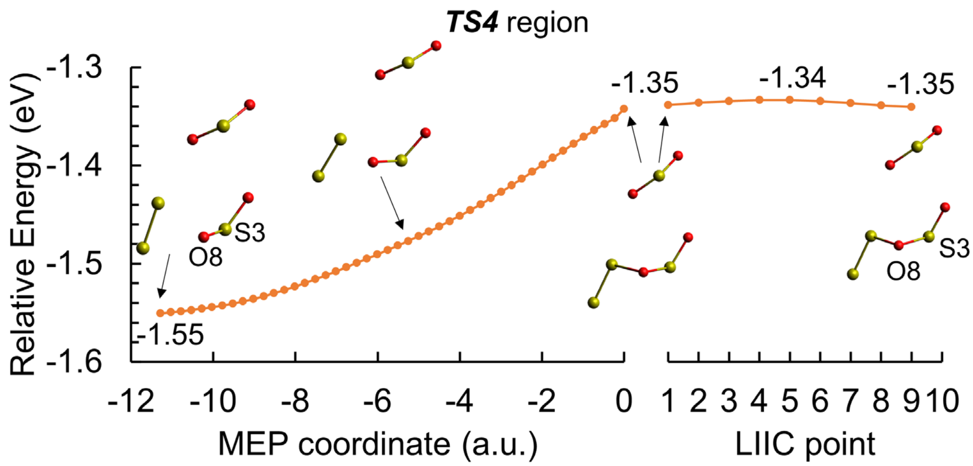

Supplementary Figure 47. Exploration of the ***TS4*** region through a combination of LIIC (right) and MEP (left) techniques. The PES is planar along the S3-O8 bond shortening from 1.644 to 1.544 Å, as shown by the LIIC profile, with a relative energy of -1.34 eV with respect to the energy of the reactants. The MEP indicates that the formation of the second $^1SO_2$ molecule and the final release of singlet diatomic sulfur $^1S_2$ is barrierless. This profile leads to the products **P2** (2 $^1SO_2$ + $^1S_2$).

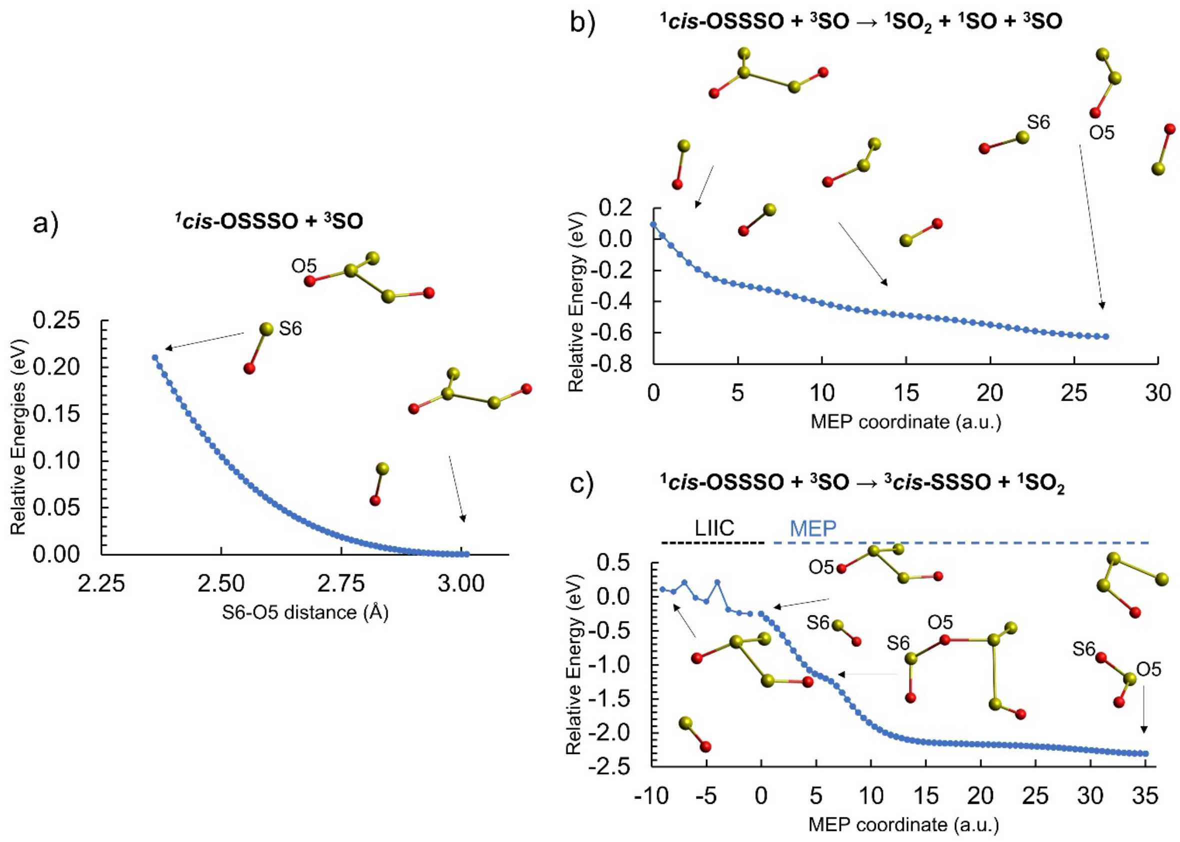

Supplementary Figure 48. ^1cis-S_3O_2 + ^3SO → ^{3}SSOSOSO → $^1SO_2$ + ^{3}SSOS reaction profiles**.** a) Energy barrier corresponding to the approach of a ^3SO molecule to $^1S_3O_2$ from the van der Waals complex to a TS-like area. b) Decomposition of $^1S_3O_2$ into $^1SO_2$, ^1SO and ^3SO computed through a MEP calculation from the TS-like area. The latter two species are expected to form ^{1}OSSO. c) Oxygen abstraction of ^3SO to form $^1SO_2$ and ^{3}SSSO. All energies are relative to the pre-reactive van der Waals complex.

7. Supplementary Note 7. Coupled-cluster results

7.1 Computational details

The ground-state reaction profiles computed in the previous section were recomputed through optimizations at the CCSD/cc-pVDZ level, employing Gaussian 16 [29]. Frequency calculations served to identify the nature of the stationary points. IRC calculations were performed at the same level of theory using the ORCA 4.2 software [30].

Single-point corrections using the CCSD(T) method were computed in combination with two larger basis sets, namely the Pople 6-311+G(3df) and the Dunning aug-cc-pVQZP, only for some selected structures. The T1 diagnostic parameter was also computed to check the possible multiconfigurational character (if T1 > 0.02) of the converged structures, and the expectation value of the total spin operator $\langle S^2 \rangle$ was employed to estimate spin contamination. Most of the structures analyzed in this work showed multiconfigurational character and significant spin contamination, particularly at regions close to the TSs, indicating that only multiconfigurational methods such as MRCI or MS-CASPT2 provide correct wave functions.

7.2 Results

Supplementary Table 9. Electronic activation energies ($\Delta E^{\ddagger}$) and energy differences between reactants and products (ΔE) for the reactivity derived from the interaction between ^{1}OSSO and ^{3}SO. All energies in kcal/mol.

	(U)B3LYP/6-311G(d,p)[a]		(U)CCSD(T)/6-311+G(3df)[a]		(U)CCSD/cc-pVDZ[b]		(U)CCSD(T)/aug-cc-pVQZ[b]		CASPT2(16,12)/ANO-L-VTZP	
**Bimolecular thermal reactions**	**$\Delta E^{\ddagger}$**	**ΔE**	**$\Delta E^{\ddagger}$**	**ΔE**	**$\Delta E^{\ddagger}$**	**ΔE**	**$\Delta E^{\ddagger}$**	**ΔE**	**$\Delta E^{\ddagger}$**	**ΔE**
^{1}OSSO + ^{3}SO → ^{3}OSSOSO	0.5	-8.2	9.1	-3.2	0.3	-13.6	13.9	-2.2	7.1	1.0
^{3}OSSOSO → ^{3}OSS + $^1SO_2$	7.2	-3.9	9.2	-10.5	13.3	-2.1	10.9	-8.3	9.0	-1.9
^{1}OSS + ^{3}SO → $^3S_2$ + $^1SO_2$	4.4	-24.1	15.6	-27.7	11.7	-24.2	15.1	-27.1	0.1	-41.7
^{1}OSSO + ^{1}OSSO → ^{1}cyclic-S_4O_4	5.0	-11.4	9.9	-0.9			9.0	-4.1		-
^{1}cyclic-S_4O_4 → ^{1}cis-S_3O_2 + $^1SO_2$	10.7	-2.3	9.3	-11.1			12.0	-6.3		-

[a] Geometries optimized with the B3LYP/6-311G(d,p) method (analytical gradients and frequencies) as implemented in Gaussian 16. IRC calculations performed with Gaussian 16.

[b] Geometries optimized with the CCSD/cc-pVDZ method (analytical gradients and numerical frequencies) as implemented in Gaussian 16. IRC calculations performed with ORCA 4.2 (numerical gradients and frequencies).

Supplementary Table 10. Gibbs activation energies ($\Delta G^{\ddagger}$) and Gibbs energy difference between reactants and products (ΔG) for the reactivity derived from the interaction between ^{1}OSSO and ^{3}SO. All energies in kcal/mol.

	(U)B3LYP/6-311G(d,p)[a,b]		UCCSD/cc-pVDZ[c,d]		UCCSD(T)/aug-cc-pVQZ[c,d]		UCCSD(T)/aug-cc-pVQZ[c,e]	
**Bimolecular thermal reactions**	**$\Delta G^{\ddagger}$**	**ΔG**	**$\Delta G^{\ddagger}$**	**ΔG**	**$\Delta G^{\ddagger}$**	**ΔG**	**$\Delta G^{\ddagger}$**	**ΔG**
^{1}OSSO + ^{3}SO → ^{3}OSSOSO	1.7	-7. 7	1.2	-13.2	14. 8	-1.7	14.4	-1.9
^{3}OSSOSO → ^{3}OSS + $^1SO_2$	7.9	-2.5	13.6	-2.3	11.2	-8.5	11.0	-8.3
^{1}OSS + ^{3}SO → $^3S_2$ + $^1SO_2$	6.3	-25.5	14.3	-25.9	17.8	-28.8	17.1	-28.4
^{1}OSSO + ^{1}OSSO → ^{1}*cyclic*-S_4O_4	6.7	-7.5	-	-	-	-	-	-
^{1}*cyclic*-S_4O_4 → ^{1}*cis*-S_3O_2 + $^1SO_2$	9.7	-5.0	-	-	-	-	-	-

[a] Geometries optimized with the B3LYP/6-311G(d,p) method (analytical gradients and frequencies) as implemented in Gaussian 16. IRC calculations performed with Gaussian 16.

[b] Gibbs energy computed at 298.15 K and 1 atm.

[c] Geometries optimized with the CCSD/cc-pVDZ method (analytical gradients and numerical frequencies) as implemented in Gaussian 16. IRC calculations performed with ORCA 4.2 (numerical gradients and frequencies).

[d] Gibbs energy computed at 298.15 K and 1 atm with the CCSD/cc-pVDZ method.

[e] Gibbs energy computed at 245 K and 0.1 atm with the CCSD/cc-pVDZ method.

Supplementary Table 11. Expected value of the total spin operator $\langle S^2 \rangle$. The ideal value for a triplet state is 2.

	UB3LYP/6-311G(d,p)[a]			UCCSD/cc-pVDZ[b]			UCCSD(T)/aug-cc-pVQZ[b]		
**Bimolecular thermal reactions**	**Reactant**	**TS**	**Product**	**Reactant**	**TS**	**Product**	**Reactant**	**TS**	**Product**
$^1OSSO + {}^3SO \rightarrow {}^3OSSOSO$	2.564	2.373	2.009	2.037	2.839	2.042	2.057	2.885	2.073
$^3OSSOSO \rightarrow {}^3OSS + {}^1SO_2$	2.009	2.026	2.007	2.042	2.526	2.244	2.073	2.530	2.090
$^1OSS + {}^3SO \rightarrow {}^3S_2 + {}^1SO_2$	2.008	2.075	2.005	2.039	2.695	2.032	2.060	2.726	2.061

[a] Geometries optimized with the B3LYP/6-311G(d,p) method (analytical gradients and frequencies) as implemented in Gaussian 16.

[b] Geometries optimized with the CCSD/cc-pVDZ method (analytical gradients and numerical frequencies) as implemented in Gaussian 16.

Supplementary Table 12. T_1 diagnostic values for coupled-cluster calculations. T_1 values >0.02 suggest the need of multireference electron correlation methods[3] such as CASPT2.

	UCCSD/cc-pVDZ[a]		
**Bimolecular thermal reactions**	**Reactant**	**TS**	**Product**
$^1OSSO + {}^3SO \rightarrow {}^3OSSOSO$	0.027	0.048	0.035
$^3OSSOSO \rightarrow {}^3OSS + {}^1SO_2$	0.035	0.055	0.042
$^1OSS + {}^3SO \rightarrow {}^3S_2 + {}^1SO_2$	0.024	0.051	0.022

[a] Geometries optimized with the CCSD/cc-pVDZ method (analytical gradients and numerical frequencies) as implemented in Gaussian 16.

7.3 Influence of the wave function initial guess and spin density analysis

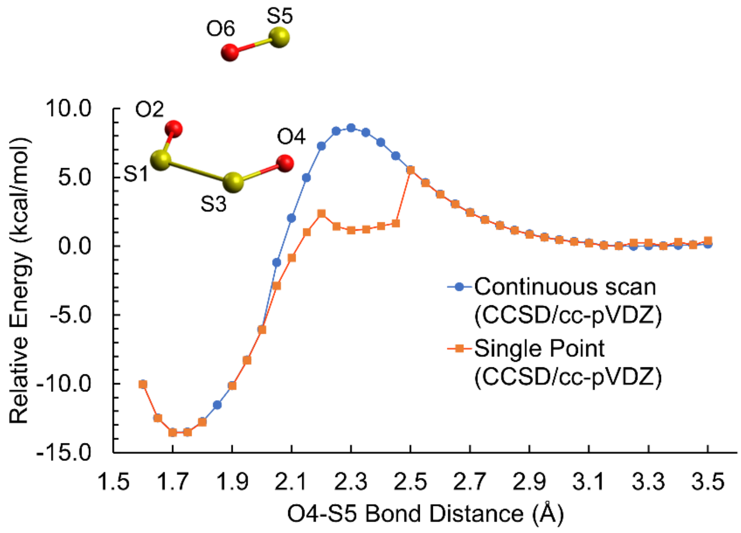

Supplementary Figure 49. CCSD/cc-pVDZ reaction profiles for ^{3}SO + ^{1}SSO → $^{1}SO_2$ + $^{3}S_2$ obtained through a continuous scan, *i.e.* each point is optimized using the wavefunction of the previous point. The "single point" curve is constructed by single points on top of the converged geometries of the continuous scan, exactly at the same level of theory. The wave function for each point is initially guessed by Gaussian 16 using its standard algorithms in each individual job, converging a different solution that the one obtained in the continuous scan in regions close to the TS.

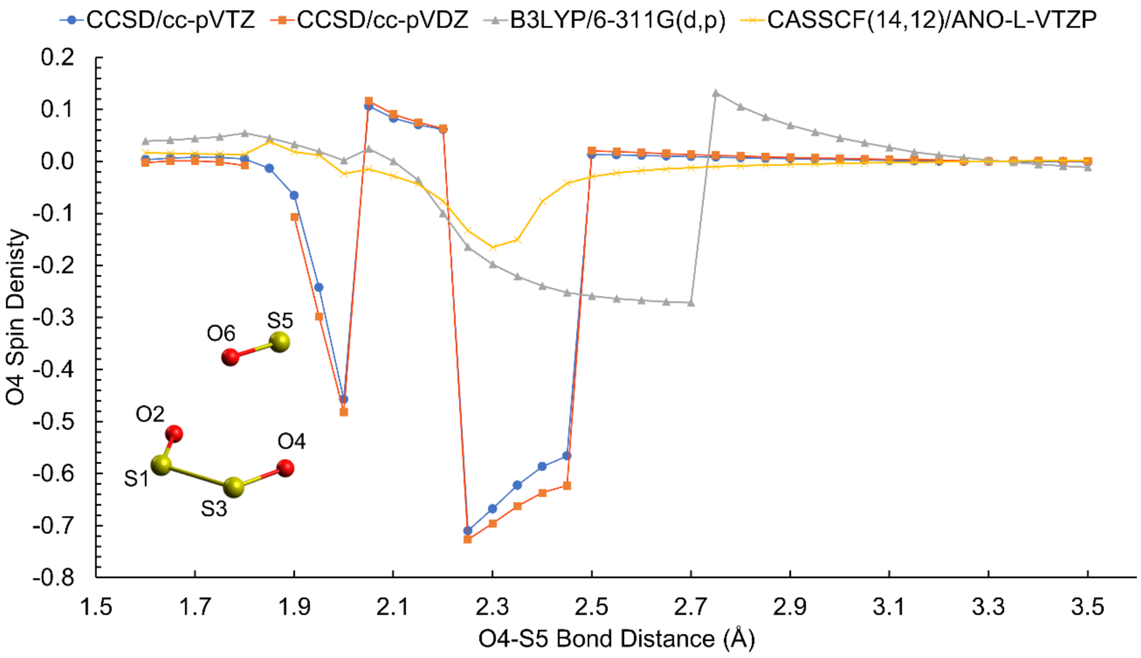

Supplementary Figure 50. Spin densities of the O4 atom through a relaxed scan of the O4-S5 bond distance at different levels of theory. The geometries are optimized at the CCSD/cc-pVDZ level of theory, however, the energies of each point are obtained by means of individual single-point calculations except for the CASSCF method, in which a set of previously converged orbitals is required to start the job. The spin densities with the monodeterminantal methods localize on different atoms than that of the CASSCF (see Supplementary Figure 26).

8. Supplementary Note 8. Estimation of sulfur species profiles in the atmosphere of Venus

8.1 Methodological details and data analyses

We use photochemical steady state calculations to estimate the abundance profiles for several sulfur species of particular importance in the Venus atmosphere. Because the photochemical lifetimes of many of the trace sulfur species are shorter than the eddy transport timescale, this approximation is valid to first order, and allows us a rapid assessment of the implications of the new chemical schemes proposed here based on our *ab initio* results. For non-sulfur species, long-lived sulfur species, and photodissociation rate constants, we use profiles from Pinto et al.[1] and Zhang et al.[27] Mixing ratio profiles for [^{3}O], [$^3O_2$], [^{2}NO], [^{2}H], [$^1SO_2$], [^{3}S], and [^{2}ClS] are digitally read-in from Zhang et al.[27] or Pinto et al.[1] Condensation reactions for sulfur allotropes have not been included here, as appears to be the case for Pinto et al. [1]

Photochemical steady state calculations are carried out from 58 to 112 km, following the temperature and total number density profiles given by Zhang et al.[27] Steady-state number densities are computed for ^{3}SO, $^1(SO)_2$, which we assume in these calculations to be primarily ^{1}*cis*-OSSO,

^1S_2O, and $^3S_2$. A reduced set of 19 reactions involving these species is given in Supplementary Table 13. Setting production rate equal to loss rate for each of the 4 species of interest, and using the reactions in Supplementary Table 13, we arrive at the equations for steady-state number density (S1) – (S4). These equations are solved in the order presented, and the steady-state values are used as applicable. For example, the steady-state number density of $^1(SO)_2$, given as $[(SO)_2]_{ss}$ and found from equation (S2), is used wherever $[(SO)_2]$ is specified in equation (S3). Loss of S_2 to S_4 formation is accounted for, but we are not attempting to accurately account for sulfur allotrope abundances. For this reason, S_2 may be taken as a proxy for total sulfur aerosol production.

Supplementary Table 13. Reactions used in the photochemical steady state model with rate constants obtained from the bibliography or from this work. See Section 8.3 for details on the origin of the rates.

	Reaction	Rate constant	Range of values
R1	$SO_2 + h\nu \rightarrow SO + O$	2.2×10^{-4} s^{-1} at 112 km	
R2	$SO + h\nu \rightarrow S + O$	4.2×10^{-4} s^{-1} at 112 km	
R3a	$(SO)_2 + h\nu \rightarrow SO + SO$	0.19 s^{-1} (*cis*); 0.56 s^{-1} (*trans*)	
R3b	$(SO)_2 + h\nu \rightarrow S_2 + O_2$	1×10^{-2} s^{-1}	0.0 – 10^{-2} s^{-1}
R4	$S_2O + h\nu \rightarrow S + SO$	5.0×10^{-2} s^{-1} at 112 km[a]	
R5	$S_2 + h\nu \rightarrow S + S$	7.7×10^{-3} s^{-1} at 112 km	
R6	$SO + SO \rightarrow SO_2 + S$	3.5×10^{-15} molecule^{-1} cm^3 s^{-1}	
R7	$SO + SO + M \rightarrow (SO)_2 + M$	1.5×10^{-30} molecule^{-2} cm^6 s^{-1}; 1.7 x 10^{-11} molecule^{-1} cm^3 s^{-1}	
R8	$(SO)_2 + M \rightarrow SO + SO + M$	0.0 molecule^{-1} cm^3 s^{-1}	
R9	$SO + (SO)_2 \rightarrow SO_2 + S_2O$	3.3×10^{-14} molecule^{-1} cm^3 s^{-1} [b]	
R10	$O + (SO)_2 \rightarrow O_2 + S_2O$	3.3×10^{-14} molecule^{-1} cm^3 s^{-1} [b]	
R11	$NO + (SO)_2 \rightarrow NO_2 + S_2O$	3.3×10^{-14} molecule^{-1} cm^3 s^{-1} [b]	
R12	$O + S_2O \rightarrow SO + SO$	1.5×10^{-12} molecule^{-1} cm^3 s^{-1}	
R13	$SO + S_2O \rightarrow SO_2 + S_2$	1.0×10^{-14} molecule^{-1} cm^3 s^{-1}	10^{-14}–10^{-10} molecule^{-1} cm^3 s^{-1}
R14	$S + S + M \rightarrow S_2 + M$	Nic: 1.2×10^{-29} molecule^{-2} cm^6 s^{-1}; 9.1 x 10^{-14} $e^{414.91(1/T - 1/298.15)}$ molecule^{-1} cm^3 s^{-1} Du: 2.4×10^{-32} $e^{205.56(1/T - 1/298.15)}$ molecule^{-2} cm^6 s^{-1}; 9.1 x 10^{-14} $e^{414.91(1/T - 1/298.15)}$ molecule^{-1} cm^3 s^{-1}	
R15	$S_2 + O \rightarrow SO + S$	2.2 x 10^{-11} $e^{-84/T}$ molecule^{-1} cm^3 s^{-1}	
R16	$S_2 + S_2 + M \rightarrow S_4 + M$	2.2×10^{-29} molecule^{-2} cm^6 s^{-1}; 1×10^{-10} molecule^{-1} cm^3 s^{-1}	
R17	$S + SO + M \rightarrow S_2O + M$	3.3×10^{-26} T^{-2} molecule^{-2} cm^6 s^{-1}	
R18	$ClS + SO \rightarrow S_2O + Cl$	1×10^{-11} molecule^{-1} cm^3 s^{-1}	
R19	$SO + S_3 \rightarrow S_2O + S_2$	1.0×10^{-12} molecule^{-1} cm^3 s^{-1}	

[a]Multiple scattering increases the rate constant from 5.0 x 10^{-2} s^{-1} at 112 km to 6.2 x 10^{-2} s^{-1} at 68 km.[27]
[b]Updated rates of R9-R11 reactions from this work and analogous reaction of $(SO)_2$ with H, compiled in Supplementary Table 6, do not affect the conclusions (see text in section 8.1).

Equations for computing approximate steady-state number densities of SO, $(SO)_2$, S_2O and S_2 given below. These expressions are approximate because *i*) we are not accounting for vertical transport, and *ii*) we are not including all possible reactions. Our objective is to get an order-of-magnitude estimate of the significance of the new branching ratios and rate coefficients determined by the *ab initio* calculations presented in this work, especially for reactions R3b and R13 (Supplementary Table 13). We performed several calculations to validate our approximate steady-state results versus the more complete models presented in Pinto et al.[1] Supplementary Figure 51 shows several reaction rates computed here ('calculated') compared to plots from Pinto et al.[1] We found good agreement among the rates for SO + $(SO)_2$ and NO + $(SO)_2$, but the reaction rate for SO + SO formation to the dimer is about a factor of 10 higher for Pinto et al.[1] compared to our calculated rate; we do not know the origin of this discrepancy. Supplementary Figure 52 shows the level of agreement for our steady-state SO and $(SO)_2$ versus the profiles in Pinto et al.[1] Our calculated values are similar to or higher than the Pinto et al. values by at most a factor of 10. The exception, shown in Supplementary Figure 53, is for S_2O. Our calculated steady-state values are ~ 100 times lower than those of Pinto et al.[1] Again, we do not know the origin of the deviation. Despite the discrepancies, note that if we use the S_2O concentrations from Pinto et al. [1], it strengthens our conclusion that SO + S_2O is a key reaction to generate S_2 and not *cis*-OSSO photodissociation..

$$[\mathrm{SO}]_{ss} = \frac{-\left(J_2\right) + \left[\left(J_2\right)^2 + 4(k_7[\mathrm{M}] + k_6)J_1[\mathrm{SO_2}]\right]^{\frac{1}{2}}}{2(k_7[\mathrm{M}] + k_6)} \qquad \text{(S1)}$$

$$[(\mathrm{SO})_2]_{ss} = \frac{k_7[\mathrm{SO}]^2[\mathrm{M}]}{J_{3a} + k_8[\mathrm{M}]} \qquad \text{(S2)}$$

$$[\mathrm{S_2O}]_{ss} = \frac{\left\{k_9[\mathrm{SO}][(\mathrm{SO})_2] + k_{10}[\mathrm{O}][(\mathrm{SO})_2] + k_{11}[\mathrm{NO}][(\mathrm{SO})_2] + k_{17}[\mathrm{S}][\mathrm{SO}][\mathrm{M}] + k_{18}[\mathrm{ClS}][\mathrm{SO}]\right\}}{\left\{J_4 + k_{12}[\mathrm{O}] + k_{13}[\mathrm{SO}]\right\}}$$

(S3)

$$[\mathrm{S_2}] = \frac{-\left(J_5 + k_{15}[\mathrm{O}]\right) + \left[\left(J_5 + k_{15}[\mathrm{O}]\right)^2 + 4k_{16}[\mathrm{M}]\left(k_{14}[\mathrm{S}]^2[\mathrm{M}] + J_{3b}[(\mathrm{SO})_2] + k_{13}[\mathrm{SO}][\mathrm{S_2O}]\right)\right]^{\frac{1}{2}}}{2k_{16}[\mathrm{M}]}$$

(S4)

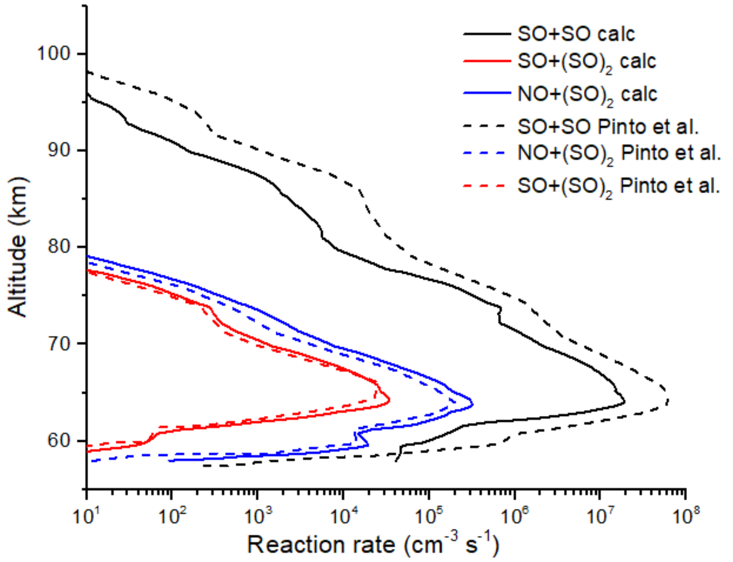

Supplementary Figure 51. Comparison of reaction rates computed using abundance profiles from Pinto et al.[1] to plots from Pinto et al.[1] We were not able to obtain complete consistency for the ^{3}SO + ^{3}SO reaction forming the SO dimer, even with a factor of 3.3 enhancement in the low pressure rate constant to account for a CO_2 atmosphere.[25]

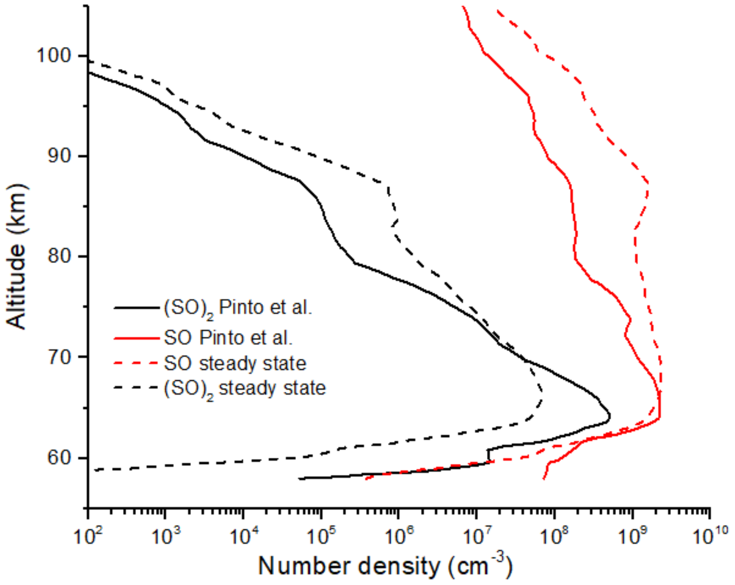

Supplementary Figure 52. Comparison of steady state computed values versus profiles from Pinto et al.[1] for ^{3}SO and the SO dimer. Agreement is acceptable for SO near 65 km, but using the photodissociation rate constants from Frandsen et al.[25] results in a low steady state $(SO)_2$ value compared to Pinto et al.[1]

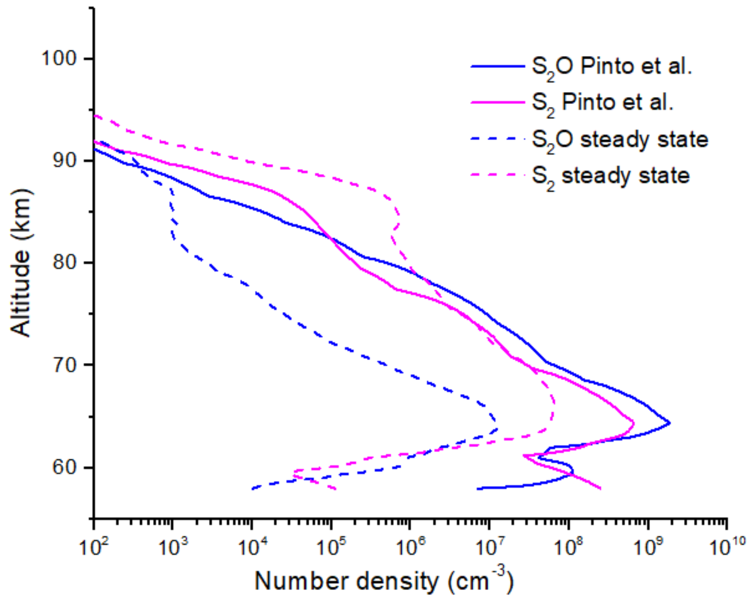

Supplementary Figure 53. Same as Supplementary Figure 52 but for ^1S_2O and $^3S_2$. In this case both our $^3S_2$ and ^1S_2O steady state values are low compared to Pinto et al. We do not know the cause of the discrepancy in ^1S_2O. Using new UV cross sections for S_2, we have increased the photodissociation rate constant for S_2, which is contributing to its lower value versus Pinto et al.[1] We note that for $^3S_2$, and all other sulfur allotropes, condensation reactions have not been included here.

8.2 Further comments on the sensitivity of the photochemical steady state model

To evaluate the effect of updated data obtained in this work for the deoxygenation reactions of $(SO)_2$, we run two steady state models (Supplementary Figures 54 and 55). In the first one (Supplementary Figure 54), we use the approximate values for the NO + OSSO and O + OSSO reactions copied from the experimental value for the SO + OSSO reaction. H profile from Zhang et al.[27] was also added, including H + OSSO reaction with a high-enough rate constant of 1×10^{-10} molecule^{-1} cm^3 s^{-1}. In the second one (Supplementary Figure 55), we used our updated high-level *ab initio* data from Supplementary Table 6. There is very little effect on the results. This is because ClS + SO and SO + OSSO completely dominate production of S_2O in the present model. These results highlight the need for future *ab initio* and laboratory studies of reactions involving S-Cl compounds.

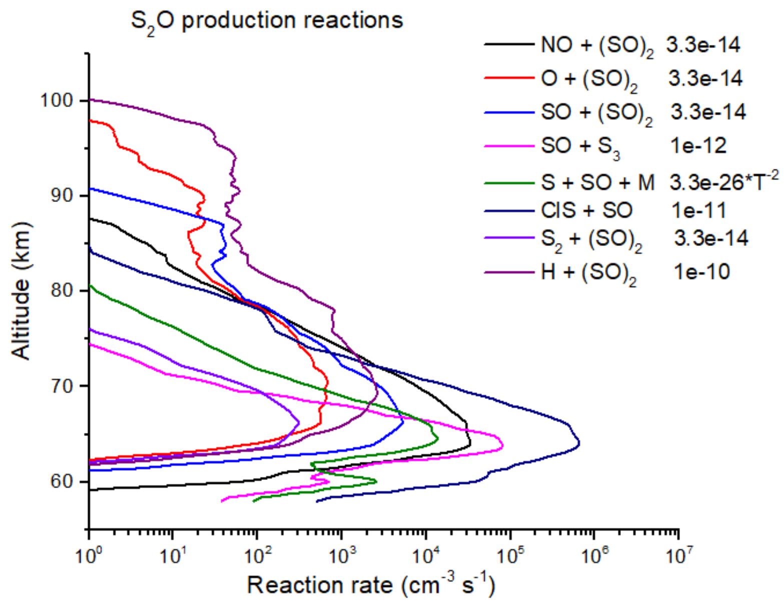

Supplementary Figure 54. Rates computed for S_2O production reactions using abundance profiles from Zhang et al.[27] and rate constants reported in the bibliography and estimated from experiments compiles in Supplementary Table 13. Rate constants for bimolecular reactions are in units of molecule^{-1} cm^3 s^{-1}. For three body reactions, the low pressure limit rate constant, in units of molecule^{-2} cm^6 s^{-1}, is considered.

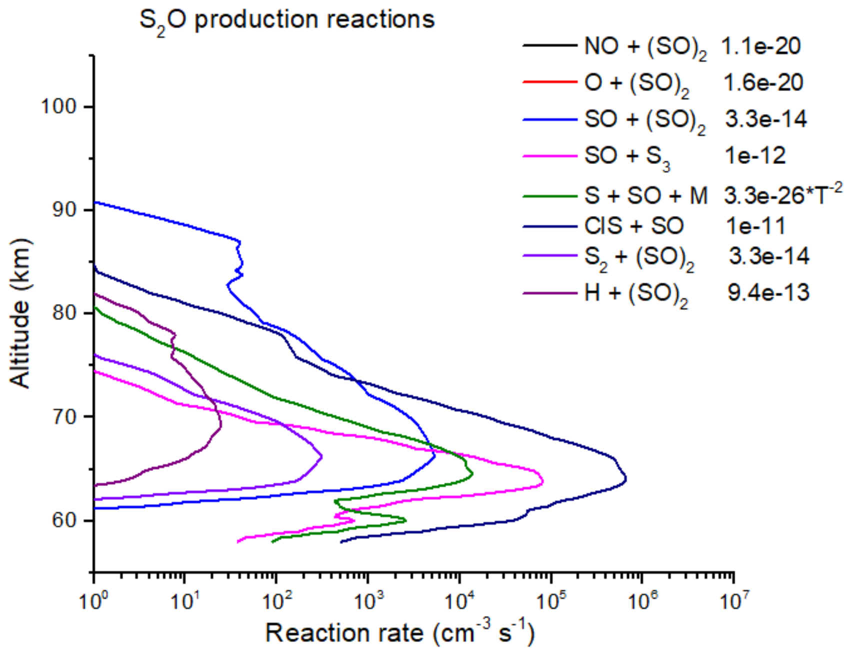

Supplementary Figure 55. Rates computed for S_2O production reactions using abundance profiles from Zhang et al.[27], similarly as in Supplementary Figure 56 but updating the rate constants for $(SO)_2$ and NO/O/SO/H with the high-level multiconfigurational quantum chemistry values computed in this work (Supplementary Table 6). Rate constants for bimolecular reactions are in units of molecule^{-1}

cm^3 s^{-1}. For three body reactions, the low pressure limit rate constant, in units of molecule^{-2} cm^6 s^{-1}, is considered.

8.3 Origin of the rates included in the photochemical steady state model

R1 $SO_2 + hv \rightarrow SO + O$

- The photolysis rate constant for SO_2 is computed at the top of the atmosphere using the solar photon flux at 1 AU from Gueymard[31] and the SO_2 dissociation cross sections from the Leiden photochemical database.[32] Wavelengths shortward of 170 nm contribute a value of 2.41×10^{-5} s^{-1}. Because we will compare our value to that of Zhang et al.[27] at 112 km, we will neglect the contribution from shortward of 170 km due to screening by the overhead column of CO_2, which we estimate to be about 2×10^{19} cm^{-2}. The B-X band from 170 to 219 nm contributes a value of 2.29×10^{-4} s^{-1}. Scaling to Venus (a factor of 1.93) and taking the diurnal average (a factor of ½) yields the total SO_2 dissociation rate constant of 2.21×10^{-4} s^{-1}. Our value is roughly 10% larger than the value of 2.0×10^{-4} s^{-1} obtained in Zhang et al.[27] at 112 km. We have scaled the J_{SO2} vertical profile from Zhang et al.[27] by the same factor at all altitudes, but this change has a minor effect.

R2 $SO + hv \rightarrow S + O$

- We have computed the photodissociation rate constant for SO using the dissociation cross sections from the Leiden database.[32] The band from 116 to 133 nm contributes 7.33×10^{-5} s^{-1}, and from 190 to 235 nm contributes 4.32×10^{-4}, both at 1 AU. Scaling to Venus and taking the diurnal average yields a total J_{SO} of 4.88×10^{-4} s^{-1} at the top of the atmosphere. Neglecting the short wavelength contribution due to overhead CO_2 absorption at 112 km, we obtain 4.17×10^{-4} s^{-1}, which is about 13% higher than the Zhang et al.[27] value of 3.7×10^{-4} s^{-1} at 112 km.

R3a $(SO)_2 + hv \rightarrow SO + SO$

- The photodissociation rates for *cis*- and *trans*-OSSO were extracted from Frandsen et al.[2]. In this work, the authors calculated these photolysis rates based on theoretically predicted UV-Vis cross sections using a nuclear ensemble approach, assuming a photolysis quantum yield of 1.0, and considering a Venusian altitude of 64 km and latitude of 0º, obtaining values of 0.20 s^{-1} and 0.62 s^{-1} for *cis*- and *trans*-OSSO, respectively. In the present study, these rates were updated to 0.19 s^{-1} and 0.56 s^{-1} by considering our computed photolysis quantum yields (0.95 and 0.9 for each system, respectively).

R3b $(SO)_2 + hv \rightarrow S_2 + O_2$

- The 1 x 10^{-2} s^{-1} value was assumed in Pinto et al.[1] based on the assignments from the experiment of Wu et al.[33] and considering 10% of S_2 production from *cis*-OSSO photolysis. The exact value considered in that work was 9.30 x 10^{-3} s^{-1}. In the present study, we have considered in our analyses both the assumption from Pinto et al. (10^{-2} s^{-1}) and zero S_2 photoproduction, being the latter based on our *ab-initio* results.

R4 $S_2O + hv \rightarrow S + SO$

- The values of the rate are extracted from Zhang et al.,[27] which refers to the Ph.D. thesis of Mills.[34] In Table 5A.1 on page 5.3 of said thesis, it is stated that the rate was estimated. In page 5-14 it is specified that the cross sections for the 260-340 nm range was estimated as 30 times the SO_2 cross section, based on earlier work by Jones,[35] while the selected quantum yield was based on measurements by Zhang et al.[36] In Frandsen et al.,[2] the authors computed the cross sections of S_2O following the same approach used for *cis/trans*-OSSO. They also compared their spectrum with the one obtained using the 30 x SO_2 cross sections estimation from F.P. Mills, being both in surprisingly good agreement. Thus, we decided to keep the values reported in Zhang et al.[27]

R5 $S_2 + h\nu \rightarrow S + S$

- Using the S_2 cross sections from about 230 to 290 nm in the Leiden database,[32] which are derived from Stark et al.,[37] we obtained a rate constant of 7.69×10^{-3} at 112 km in Venus. This is nearly a factor of 2 larger than the Zhang et al. value of 4.0×10^{-3} s^{-1}, and results from the substantial improvement in S_2 cross sections presented in Stark et al.

R6 $SO + SO \rightarrow SO_2 + S$

- The value of 3.5 x 10^{-15} cm^3 s^{-1} at 298K was taken from Martinez and Herron,[38] study in which this rate was determined experimentally.

R7 $SO + SO + M \rightarrow (SO)_2 + M$

- Regarding the low-pressure limit of 4.4 x 10^{-31} cm^6 s^{-1}, this value was taken from Zhang et al.,[27] which refers back to the Ph.D. thesis of Mills.[34] In Table 5C.2 and page 5-34 of said thesis it is stated that the rate was taken directly from Herron and Huie.[26] In Krasnopolsky,[39] the low pressure limit rate of Herron and Huie[26] was scaled by 2.5 to account for the difference in the bath gas in their experiment (N_2) compared to the Venusian atmosphere, which is mainly CO_2. In Frandsen et al.,[40] a factor of 3.3 was used as well to account for the different bath gas. Thus, we have adopted the latter factor of 3.3 in our simulations.
- In the case of the high-pressure limit rate constant, the value of 1.7 x 10^{-11} cm^3 s^{-1} determined using computational methods in Frandsen et al.[2] was used.

R8 $(SO)_2 + M \rightarrow SO + SO + M$

- For the present calculations, we neglect thermal decomposition of the SO dimer. The rate coefficient for thermal decomposition given in Zhang et al.[27] becomes smaller at higher temperature, which we consider to be problematic. We therefore set this rate constant to zero.

R9 $SO + (SO)_2 \rightarrow SO_2 + S_2O$

- The value of 3.3 x 10^{-14} cm^3 s^{-1} was taken from Herron and Huie,[26] study in which experimental and modelling work was carried out to obtain the rate. In Pinto et al.,[1] this rate is also included but the referenced source was Zhang et al.[27] In the latter work, Moses et al.[41] is cited as the original source, which again refers to Herron and Huie.[26] In addition, the value of the rate, depending on the study, is 3.3 x 10^{-14} cm^3 s^{-1} or 3.0 x 10^{-14} cm^3 s^{-1}, although 3.3 x 10^{-14} cm^3 s^{-1} seems to be the original value in Herron and Huie.[26] In our work, an updated

value for this rate was obtained according to our *ab initio* results and using conventional transition state theory.

R10 $O + (SO)_2 \rightarrow O_2 + S_2O$

- The value of 3.3 x 10^{-14} cm^3 s^{-1} was taken from Yung and Demore,[42] study in which the authors postulated that the rate of this reaction was the same as the rate for R9 from Herron and Huie.[26] In our work, an updated value for this rate was obtained according to our *ab initio* results and using conventional transition state theory.

R11 $NO + (SO)_2 \rightarrow NO_2 + S_2O$

- The value of 3.3 x 10^{-14} cm^3 s^{-1} was taken from Pinto et al.[1] in which it was postulated that the rate for this reaction was the same as for R9. In the Supporting Information of said work, it is stated that this estimation is based on Zhang et al.[27] In our study, an updated value for this rate was obtained according to our *ab initio* results and using conventional transition state theory.

R12 $O + S_2O \rightarrow SO + SO$

- The value of 1.5 x 10^{-12} cm^3 s^{-1} was taken from Zhang et al.,[27] although the specific value that appears in Table 2 of said study is 1.7 x 10^{-12} cm^3 s^{-1}. In this work, the Ph.D. thesis of Mills [34] is cited as the original source, which contains the same value for the rate. In Moses et al.,[41] the value of the rate is 1.5 x 10^{-12} cm^3 s^{-1}. However, for both the latter study and the Ph.D thesis, the cited original source is Singleton and Cvetanović,[43] which cites an earlier work: Stedman et al.,[44] which is an experimental work on the chemiluminescent reactions of S_2O. In this study, the estimate of the rate constant is 1.5 ± 0.2 x 10^{-12} cm^3 s^{-1}, suggesting that the differences in the reported rates are within the errors of the original source.

R13 $SO + S_2O \rightarrow SO_2 + S_2$

- The value of 1.0 x 10^{-14} cm^3 s^{-1} is taken in the present study as a lower limit assuming a similar reaction rate as R9. We have considered a range of values for the rate in our modelling analyses, considering the above-mentioned assumption for the lower limit (10^{-14} cm^3 s^{-1}) and our *ab initio* results for the upper one (10^{-10} cm^3 s^{-1}).

R14 $S + S + M \rightarrow S_2 + M$

- For the low-pressure limit rate constant of this reaction there are two experimental values reported in the literature. In the model of Zhang et al.,[27] the rate used is 1.18 x 10^{-29} cm^6 s^{-1} and the source is the Ph.D. thesis of Mills.[34] As stated in page 5-25 of said thesis, this value was taken from Nicholas et al.,[45] in which the authors determined the rate experimentally at 295 K and using H_2S as the bath gas. On the other hand, a value for the rate of 2.8 x 10^{-33} cm^6 s^{-1} is reported on Fair and Thrush,[46] obtained at 298 K with Ar as the bath gas. As can be seen, both results differ by 4 orders of magnitude.

  In Du et al.,[28] the low-pressure limit rate constant of this reaction was calculated using quasiclassical trajectory calculations, considering Ar as the bath gas, to clarify this discrepancy and obtained a value of 4.19 x 10^{-33} cm^6 s^{-1} (3.94 x 10^{-33} $e^{205.56(1/T - 1/298.15)}$ cm^6 s^{-1}) at 298.15K which is in excellent agreement with the result of Fair and Thrush [46]. Thus, we

considered the rate reported in Du et al.[28] assuming a rate enhancement of 6.0 for CO_2 compared to Ar as the 3rd-body collision partner, estimating in this way that the bath gas in the Venusian atmosphere is mainly CO_2.

We arrived at this value of the bath gas efficiency based on a comparison of Singleton and Cvetanović,[43] which reported an enhancement of 8.2 for the reaction $O + SO \xrightarrow{M} SO_2$ in CO_2 versus Ar, and Choudhary et al.[47] which found an enhancement of 4.3 to 6.7 for the reaction $H + O_2 \xrightarrow{M} HO_2$ at temperatures of 1000 – 2000 K for the same two bath gases. From the wide range of experimental values from these two papers, and from the wide range of values assumed in previous work on the Venus atmosphere, it is clear that the uncertainties in the photochemistry due to uncertainties in the bath gas (i.e., CO_2) kinetics are substantial, and is a topic that needs to be addressed in future work.

- Regarding the high-pressure limit rate constant, the reported value from Du et al.[28] was considered (1.03 x 10^{-13} cm^3 s^{-1} at 298.15 K, 9.07 x 10^{-14} $e^{414.91(1/T - 1/298.15)}$ cm^3 s^{-1}).

R15 $S_2 + O \rightarrow SO + S$

- The temperature dependent rate constant of 2.20 x 10^{-11} $e^{-84/T}$ $molecule^{-1}$ cm^3 s^{-1} was taken from Zhang et al.[27] The original source cited in Zhang et al.[27] is the work of Moses et al.,[41] which refers back to Craven and Murrell,[48] study in which this rate was determined by means of quasi-classical trajectory calculations.

R16 $S_2 + S_2 + M \rightarrow S_4 + M$

- Both low- and high-pressure limit rate constants were taken from Zhang et al.,[27] which refers back to the Ph.D thesis of Mills.[34] In Table 5C.2 of said thesis, it is stated that the low pressure limit rate constant, with a value of 2.2 x 10^{-29} cm^6 s^{-1}, was taken from Nicholas et al.,[45] while the value of 1.0 x 10^{-10} cm^3 s^{-1} for the high-pressure limit was taken from Fowles et al.[49]

R17 $S + SO + M \rightarrow S_2O + M$

- The value of 3.3 x 10^{-26} T^{-2} cm^6 s^{-1} was taken from Zhang et al.,[27] which refers to Moses et al.[41] as the original source. In this work, the rate was estimated (Table A3). In addition, this rate is already corrected by a factor of 3.3 for the higher efficiency of the third body CO_2 than N_2, as stated in the footnote of Table 2 in Zhang et al.[27]

R18 $ClS + SO \rightarrow S_2O + Cl$

- The value of 1.0 x 10^{-11} cm^3 s^{-1} was taken from Zhang et al.,[27] which refers to Moses et al.[41] as the original source. In this work, the rate was estimated (Table A3).

R19 $SO + S_3 \rightarrow S_2O + S_2$

- The value of 1.0 x 10^{-12} cm^3 s^{-1} was taken from Zhang et al.[27] which refers to Moses et al.[41] as the original source. In this work, the rate was estimated (Table A3).

In addition, for reaction $S + O_2 \rightarrow SO + O$, which is only included in Supplementary Table 6, the value of 2.3 x 10^{-12} cm^3 s^{-1} was taken from Zhang et al.,[27] which refers to JPL-15[50] as the original source.

We have also considered the reaction $H + (SO)_2 \rightarrow S_2O + OH$. A value of 3.0 x 10^{-14} cm^3 s^{-1} can be found on Pinto et al.[1] where it was postulated that the rate for this reaction was the same as for R9. In our work, we considered both a high-enough rate constant of 1×10^{-10} $molecules^{-1}$ cm^3 s^{-1} and our updated high-level *ab initio* data.

Finally, we have considered the reaction $S_2 + (SO)_2 \rightarrow 2S_2O$. The value of 3.3 x 10^{-14} cm^3 s^{-1} for the rate constant was taken from Zhang et al.,[27] which refers to the Ph.D. thesis of Mills[34] in which is stated that the rate was estimated.

9. Supplementary References

1. Pinto, J. P. *et al.* Sulfur monoxide dimer chemistry as a possible source of polysulfur in the upper atmosphere of Venus. *Nat. Commun.* **12**, 175 (2021).
2. Frandsen, B. N., Farahani, S., Vogt, E., Lane, J. R. & Kjaergaard, H. G. Spectroscopy of OSSO and Other Sulfur Compounds Thought to be Present in the Venus Atmosphere. *J. Phys. Chem. A* **124**, 7047–7059 (2020).
3. Lee, T. J. & Taylor, P. R. A Diagnostic for Determining the Quality of Single-Reference Electron Correlation Methods. *Int. J. Quantum Chem.* **36**, 199–207 (1989).
4. Werner, H. J. & Knowles, P. J. An efficient internally contracted multiconfiguration-reference configuration interaction method. *J. Chem. Phys.* **89**, 5803–5814 (1988).
5. Knowles, P. J. & Werner, H. J. An efficient method for the evaluation of coupling coefficients in configuration interaction calculations. *Chem. Phys. Lett.* **145**, 514–522 (1988).
6. Werner, H. J. *et al.* The Molpro quantum chemistry package. *J. Chem. Phys.* **152**, 144107 (2020).
7. Mai, S. *et al.* SHARC2.1: Surface Hopping Including Arbitrary Couplings – Program Package for Non-Adiabatic Dynamics. (2019).
8. Mai, S., Marquetand, P. & González, L. Nonadiabatic dynamics: The SHARC approach. *Wiley Interdiscip. Rev. Comput. Mol. Sci.* **8**, e1370 (2018).
9. Andersson, K., Malmqvist, P. & Roos, B. O. Second-order perturbation theory with a complete active space self-consistent field reference function. *J. Chem. Phys.* **96**, 1218–1226 (1992).
10. Finley, J., Malmqvist, P. Å., Roos, B. O. & Serrano-Andrés, L. The multi-state CASPT2 method. *Chem. Phys. Lett.* **288**, 299–306 (1998).
11. Roca-Sanjuán, D., Aquilante, F. & Lindh, R. Multiconfiguration second-order perturbation theory approach to strong electron correlation in chemistry and photochemistry. *Wiley Interdiscip. Rev. Comput. Mol. Sci.* **2**, 585–603 (2012).

12. Fdez. Galván, I. *et al.* OpenMolcas: From Source Code to Insight. *J. Chem. Theory Comput.* **15**, 5925–5964 (2019).

13. Granucci, G., Persico, M. & Zoccante, A. Including quantum decoherence in surface hopping. *J. Chem. Phys.* **133**, 134111 (2010).

14. Plasser, F. *et al.* Strong Influence of Decoherence Corrections and Momentum Rescaling in Surface Hopping Dynamics of Transition Metal Complexes. *J. Chem. Theory Comput.* **15**, 5031–5045 (2019).

15. Ghigo, G., Roos, B. O. & Malmqvist, P. Å. A modified definition of the zeroth-order Hamiltonian in multiconfigurational perturbation theory (CASPT2). *Chem. Phys. Lett.* **396**, 142–149 (2004).

16. Forsberg, N. & Malmqvist, P. Å. Multiconfiguration perturbation theory with imaginary level shift. *Chem. Phys. Lett.* **274**, 196–204 (1997).

17. Francés-Monerris, A. *et al.* Photodissociation Mechanisms of Major Mercury(II) Species in the Atmospheric Chemical Cycle of Mercury. *Angew. Chemie Int. Ed.* **59**, 7605–7610 (2020).

18. Carmona-García, J. *et al.* Photochemistry and Non-adiabatic Photodynamics of the HOSO Radical. *J. Am. Chem. Soc.* **143**, 10836–10841 (2021).

19. Runge, E. & Gross, E. K. U. Density-functional theory for time-dependent systems. *Phys. Rev. Lett.* **52**, 997–1000 (1984).

20. CASIDA, M. E. Time-Dependent Density Functional Response Theory for Molecules. in *Recent Advances in Density Functional Methods (Part I)* (ed. Chong, D. P.) 155–192 (World Scientific, 1995). doi:10.1142/9789812830586_0005.

21. Becke, A. D. Density-functional thermochemistry. III. The role of exact exchange. *J. Chem. Phys.* **98**, 5648–5652 (1993).

22. Stephens, P. J., Devlin, F. J., Chabalowski, C. F. & Frisch, M. J. Ab Initio calculation of vibrational absorption and circular dichroism spectra using density functional force fields. *J. Phys. Chem.* **98**, 11623–11627 (1994).

23. Frisch, M. J. *et al.* Gaussian 09, Revision C.01, Gaussian, Inc., Wallingford, CT. (2016).

24. Barbatti, M. *et al.* NEWTON-X: a package for Newtonian dynamics close to the crossing seam, version 1.2. *Max-Planck-Institut für Kohlenforsch. Mülheim an der Ruhr, Ger.* (2011).

25. Frandsen, B. N., Wennberg, P. O. & Kjaergaard, H. G. Identification of OSSO as a near-UV absorber in the Venusian atmosphere. *Geophys. Res. Lett.* **43**, 11146–11155 (2016).

26. Herron, J. T. & Huie, R. E. Rate constants at 298 k for the reactions SO+SO+M→(SO)2+M AND SO+(SO)2→SO2+S2O. *Chem. Phys. Lett.* **76**, 322–324 (1980).

27. Zhang, X., Liang, M. C., Mills, F. P., Belyaev, D. A. & Yung, Y. L. Sulfur chemistry in the middle atmosphere of Venus. *Icarus* **217**, 714–739 (2012).

28. Du, S., Francisco, J. S., Shepler, B. C. & Peterson, K. A. Determination of the rate constant for sulfur recombination by quasiclassical trajectory calculations. *J. Chem. Phys.* **128**, 204306 (2008).

29. Frisch, M. J. *et al.* G16_C01. Gaussian 16, Revision C.01, Gaussian, Inc., Wallin (2016).

30. Neese, F. Software update: the ORCA program system, version 4.0. *Wiley Interdiscip. Rev. Comput. Mol. Sci.* **8**, e1327 (2018).

31. Gueymard, C. A. The sun's total and spectral irradiance for solar energy applications and solar radiation models. *Sol. Energy* **76**, 423–453 (2004).

32. Heays, A. N., Bosman, A. D. & Van Dishoeck, E. F. Photodissociation and photoionisation of atoms and molecules of astrophysical interest. *Astron. Astrophys.* **602**, A105 (2017).

33. Wu, Z. *et al.* The near-UV absorber OSSO and its isomers. *Chem. Commun.* **54**, 4517–4520 (2018).

34. Mills, F. P. I. Observations and Photochemical Modeling of the Venus Middle Atmosphere. II. Thermal Infrared Spectroscopy of Europa and Callisto. **1998**, (1998).

35. Jones, A. V. Infra-red and ultraviolet spectra of sulphur monoxide. *J. Chem. Phys.* **18**, 1263–1268 (1950).

36. Zhang, Q., Dupré, P., Grzybowski, B. & Vaccaro, P. H. Laser-induced fluorescence studies of jet-cooled S2O: Axis-switching and predissociation effects. *J. Chem. Phys.* **103**, 67–79 (1995).

37. Stark, G. *et al.* Fourier-transform-spectroscopic photoabsorption cross sections and oscillator strengths for the S2 B Σ u - 3 - X Σ g - 3 system. *J. Chem. Phys.* **148**, 244302 (2018).

38. Martinez, R. I. & Herron, J. T. Methyl thiirane: Kinetic gas-phase titration of sulfur atoms in SX OY systems. *Int. J. Chem. Kinet.* **15**, 1127–1132 (1983).

39. Krasnopolsky, V. A. A photochemical model for the Venus atmosphere at 47–112km. *Icarus* **218**, 230–246 (2012).

40. Frandsen, B. N., Wennberg, P. O. & Kjaergaard, H. G. Identification of OSSO as a near-UV absorber in the Venusian atmosphere. *Geophys. Res. Lett.* **43**, 11,146-11,155 (2016).

41. Moses, J. I., Zolotov, M. Y. & Fegley, B. Photochemistry of a volcanically driven atmosphere on Io: Sulfur and oxygen species from a pele-type eruption. *Icarus* **156**, 76–106 (2002).

42. Yung, Y. L. & Demore, W. B. Photochemistry of the stratosphere of Venus: Implications for atmospheric evolution. *Icarus* **51**, 199–247 (1982).

43. Singleton, D. L. & Cvetanović, R. J. Evaluated Chemical Kinetic Data for the Reactions of Atomic Oxygen O(3 P) with Sulfur Containing Compounds. *J. Phys. Chem. Ref. Data* **17**, 1377–1437 (1988).

44. Stedman, D. H., Alvord, H. & Baker-Blocker, A. Chemiluminescent reactions of disulfur monoxide. *J. Phys. Chem.* **78**, 1248–1250 (1974).

45. Nicholas, J. E., Amodio, C. A. & Baker, M. J. Kinetics and mechanism of the decomposition of H2S, CH3SH and (CH3)2S in a radio-frequency pulse discharge. *J. Chem. Soc. Faraday Trans. 1 Phys. Chem. Condens. Phases* **75**, 1868–1875 (1979).

46. Fair, R. W. & Thrush, B. A. Mechanism of S2 chemiluminescence in the reaction of hydrogen atoms with hydrogen sulphide. *Trans. Faraday Soc.* **65**, 1208–1218 (1969).

47. Choudhary, R. *et al.* Measurement of the reaction rate of H + O2 + M → HO2 + M, for M= Ar, N2, CO2, at high temperature with a sensitive OH absorption diagnostic. *Combust.*

Flame **203**, 265–278 (2019).

48. Craven, W. & Murrell, J. N. Trajectory studies of S + O2 and O + S2 collisions. *J. Chem. Soc. Faraday Trans. 2 Mol. Chem. Phys.* **83**, 1733–1741 (1987).

49. Fowles, P., DeSorgo, M., Yarwood, A. J., Strausz, O. P. & Gunning, H. E. The Reactions of sulfur Atoms. IX. The Flash Photolysis of Carbonyl Sulfide and the Reactions of S(1 D) Atoms with Hydrogen and Methane. *J. Am. Chem. Soc.* **89**, 1352–1362 (1967).

50. Sander, S. P. *et al.* Chemical Kinetics and Photochemical Data for Use in Atmospheric Studies Evaluation Number 15. *Cross Sect.* **California**, 1–153 (2006).
